# Development and Characterization of Potent Succinate Receptor Fluorescent Tracers

**DOI:** 10.1021/acs.jmedchem.3c00552

**Published:** 2023-06-15

**Authors:** Marija Ciba, Bethany Dibnah, Brian D. Hudson, Elisabeth Rexen Ulven

**Affiliations:** † Department of Drug Design and Pharmacology, University of Copenhagen, Universitetsparken 2, DK-2100 Copenhagen, Denmark; § Centre for Translational Pharmacology, School of Molecular Biosciences, College of Medical, Veterinary and Life Sciences, University of Glasgow, Glasgow G12 8QQ, Scotland United Kingdom

## Abstract

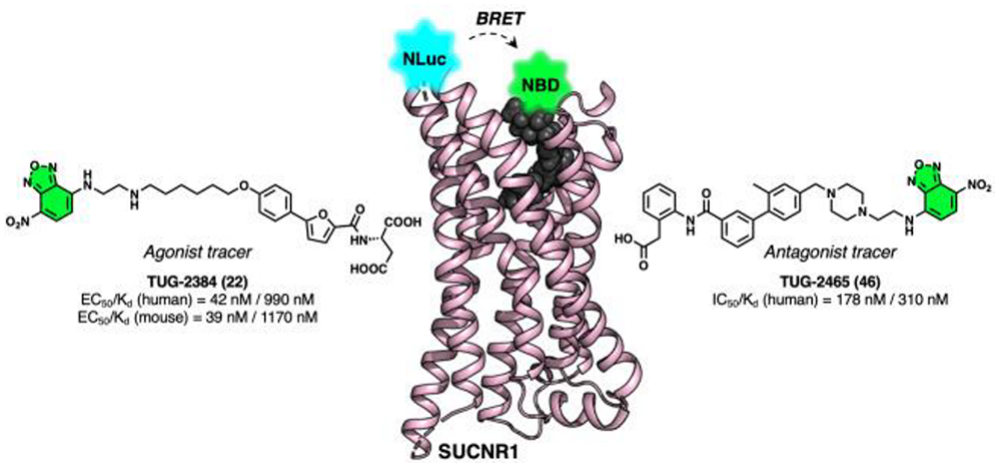

The succinate receptor (SUCNR1) has emerged as a potential target for the treatment of various metabolic and inflammatory diseases, including hypertension, inflammatory bowel disease, and rheumatoid arthritis. While several ligands for this receptor have been reported, species differences in pharmacology between human and rodent orthologs have limited the validation of SUCNR1’s therapeutic potential. Here, we describe the development of the first potent fluorescent tool compounds for SUCNR1 and use these to define key differences in ligand binding to human and mouse SUCNR1. Starting from known agonist scaffolds, we developed a potent agonist tracer, TUG-2384 (**22**), with affinity for both human and mouse SUCNR1. In addition, we developed a novel antagonist tracer, TUG-2465 (**46**), which displayed high affinity for human SUCNR1. Using **46** we demonstrate that three humanizing mutations on mouse SUCNR1, N18^1.31^E, K269^7.32^N, and G84^EL1^W, are sufficient to restore high-affinity binding of SUCNR1 antagonists to the mouse receptor ortholog.

## Introduction

Long viewed primarily as a metabolic intermediate of the tricarboxylic acid cycle, succinate has recently been attributed novel physiological roles beyond cellular energy production. In 2004, succinate was reported as the endogenous ligand for GPR91, subsequently renamed the succinate receptor (SUCNR1),^1,2^ a G protein-coupled receptor mainly signaling through a Gα_i_-mediated pathway.^3−7^ The receptor is expressed in a wide range of cells and tissues, including kidney,^1^ liver,^8^ adipose,^9^ heart,^10^ retina,^11^ and immune cells.^12,13^ SUCNR1 has been implicated in a range of pathological conditions such as hypertension,^14−16^ liver fibrosis,^17,18^ rheumatoid arthritis,^19^ age-related macular degeneration,^20^ cancer,^21,22^ and periodontitis.^23^ Many studies suggest a pro-inflammatory role of the succinate-induced stimulation of SUCNR1. For example, the succinate-SUCNR1 axis drives the Toll-like receptor-induced inflammatory cytokine production in dendritic cells,^12^ while SUCNR1 activation in pro-inflammatory macrophages results in increased production of IL-1β cytokines.^19^ In contrast, several other studies indicate an anti-inflammatory role of SUCNR1 stimulation in myeloid cells^24^ and neural stem cells.^25^ Although there is a growing body of evidence suggesting that inhibition of SUCNR1 is of therapeutic interest, the conflicting roles of the receptor in inflammation leave the space open for therapeutic development of both agonists and antagonists.

A number of synthetic SUCNR1 modulators have been reported, including a series of naphthyridines,^26^ non-metabolite partial agonists with nanomolar potency at mouse (m) and human (h) SUCNR1,^2^ and a nanomolar potency human-specific antagonist NF-56-EJ40 (**1**).^27^ The lack of high-quality tool compounds and in particular antagonists with activity on rodent receptor orthologs is the major obstacle for *in vivo* studies to validate the therapeutic potential of SUCNR1.

**Figure 1:**
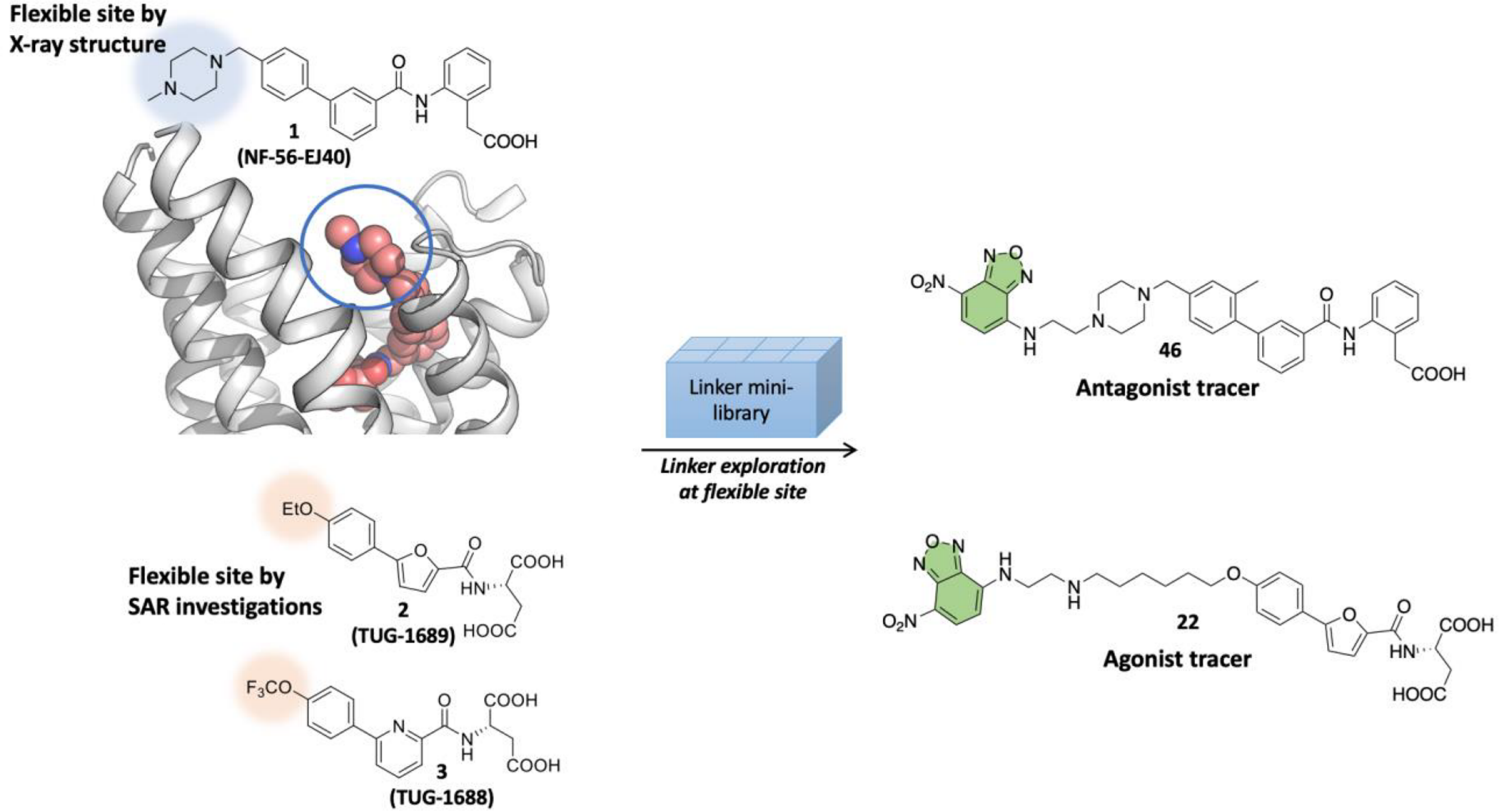
**Design strategy of antagonist and agonist tracers.** The flexible sites identified either by the published X-ray complex of the antagonist **1** or by SAR investigations of the agonists **2** and **3** were explored using a small linker library to identify the optimal linker for attachment of the NBD-fluorophore.

Fluorescently labeled ligands have proven to be valuable pharmacological tools for investigations of ligand binding to GPCRs.^28^ The fluorescent tracers possess many advantages over conventional radiolabeled tracers, including practical convenience concerning safety, waste disposal, and cost. Moreover, fluorescence-based binding assays enable studies of real-time binding and visualization of ligand–receptor complexes in intact cells and allow for non-wash homogeneous HTS-compatible assay formats when employed with resonance energy transfer (RET)-based techniques.^28−31^ Nano bioluminescence resonance energy transfer (NanoBRET) assays have been successfully used to investigate binding of ligands targeting other carboxylate-sensing receptors, including the free fatty acid receptors, FFA1 and FFA2. The solvatochromic dye 4-amino-7-nitrobenzoxadiazole (NBD) has been employed in these FFA1 and FFA2 tracers,^32,33^ and although it has low brightness and fluorophores emitting in the green/yellow sometimes present challenges as tracers due to cellular autofluorescence, NBD has an absorption band that overlaps almost perfectly with the Nanoluciferase (Nluc) emission band, is relative easy to introduce at primary amines, and has a good chemical stability. Therefore, we aimed to develop potent NBD-based fluorescent agonist and antagonist tracers for SUCNR1 to be used in a NanoBRET assay^34^ based on current SAR insight for SUCNR1 agonists TUG-1689 (**2**) and TUG-1688 (**3**)^2^ and the recent antagonist **1**(27) (Figure 1). We then aimed to use these tracers to define the basis for the lack of binding affinity for **1** and other SUCNR1 antagonists at the mouse ortholog of SUCNR1.

## Results and Discussion

**Scheme 1:**
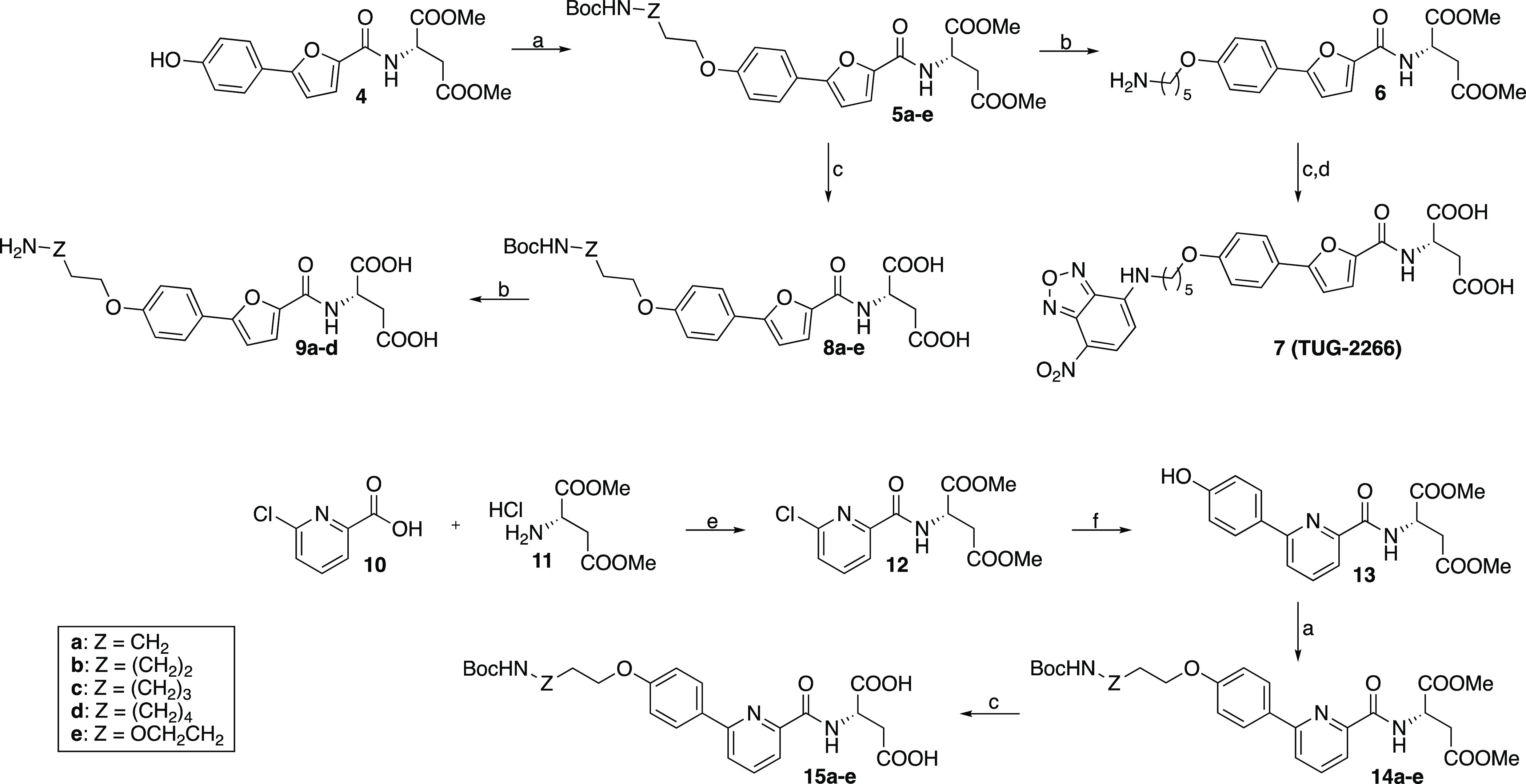
Synthesis of Agonist Tracer Precursors and the First Agonist Tracer **7** Reagents and conditions: (a) BocNH(CH_2_CH_2_O)_2_Ts or BocNH(CH_2_)_2–6_X or -OTs, K_2_CO_3_, MeCN, 80 °C, 21–48 h, 23–79%; (b) 4 M HCl in 1,4-dioxane, DCM, rt, 20–48 h, 15–94%; (c) 0.6 M LiOH (aq), THF, rt, 12–39 h, 53–100%; (d) NBD-Cl, NEt_3_, MeOH, rt, 18 h, 40%; (e) BTFFH, DIPEA, DCM, 80 °C, 12 h, 88%; (f) 4-hydroxyphenylboronic acid, XPhos-Pd-G4, 0.5 M K_3_PO_4_ (aq), THF, rt, 22 h, 62%.

The agonist tracer precursors were synthesized from intermediate **4**(2) or by a fluoro-*N,N,N′,N′-*bis(tetramethylene)formamidinium hexafluorophosphate (BTFFH)-mediated amide coupling between 6-chloropicolinic acid (**10**) and the dimethyl ester of l-aspartic acid (**11**) (Scheme 1).^35^ The amide intermediate **12** underwent Suzuki coupling with *para*-hydroxyphenylboronic acid using the fourth-generation XPhos precatalyst, and the phenol was alkylated with various Boc-protected aminoalkyl halides and tosylates. The esters were hydrolyzed under basic conditions to give the Boc-protected tracer precursors, while the free amine tracer precursors underwent HCl-mediated Boc-deprotection before hydrolysis. The first NBD tracer **7** was synthesized from the Boc-deprotected methyl ester intermediate **6** by substitution with NBD-Cl followed by ester hydrolysis.

**Scheme 2:**
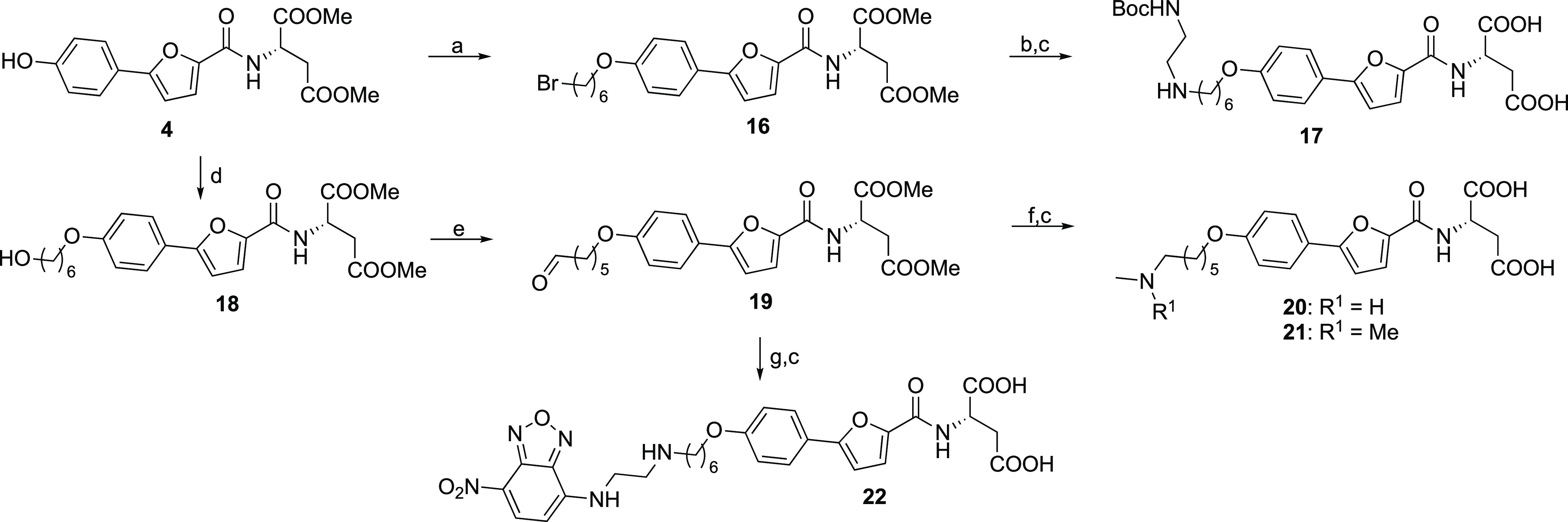
Synthesis of Optimized Agonist Tracer Precursors and Agonist Tracer **22** Reagents and conditions: (a) Br(CH_2_)_6_Br, K_2_CO_3_, KI, MeCN, 80 °C, 3.5 h, 71%; (b) BocNH(CH_2_)_2_NH_2_, K_2_CO_3_, KI, MeCN, 80 °C, 52 h, 41%; (c) 0.6 M LiOH (aq), THF, rt, 2–53 h, 43–96%; (d) Br(CH_2_)_6_OH, K_2_CO_3_, KI, MeCN, 80 °C, 22 h, 43%; (e) DMP, NaHCO_3_, DCM, 0 °C, 2 h, 82%; (f) CH_3_NH_2_·HCl or (CH_3_)_2_NH·HCl, NaBH(OAc)_3_, NEt_3_, DCE, rt, 2–3 h, 11–17%; (g) NBD-NH(CH_2_)_2_NH_2_, NaBH(OAc)_3_, THF, rt, 23 h, 8%.

The extended amine linker analogue **17** was synthesized from the phenol intermediate **4** by first an alkylation with 1,6-dibromohexane followed by substitution with *tert*-butyl (2-aminoethyl)carbamate and finally hydrolysis of the esters (Scheme 2). **4** was alkylated with 6-bromohexan-1-ol followed by oxidation of the hydroxy intermediate (**18**) to the corresponding aldehyde **19** that was subjected to reductive aminations with methyl- and dimethylamine hydrochlorides followed by base-promoted ester hydrolyses to give *N*-methylated and *N,N*-dimethylated analogues **20** and **21**.

To get NBD attached selectively at the primary amine, the tracer was synthesized from aldehyde **19** that underwent reductive amination with NBD coupled to diaminoethyl, and hydrolysis of the esters gave the tracer **22**.

**Scheme 3:**
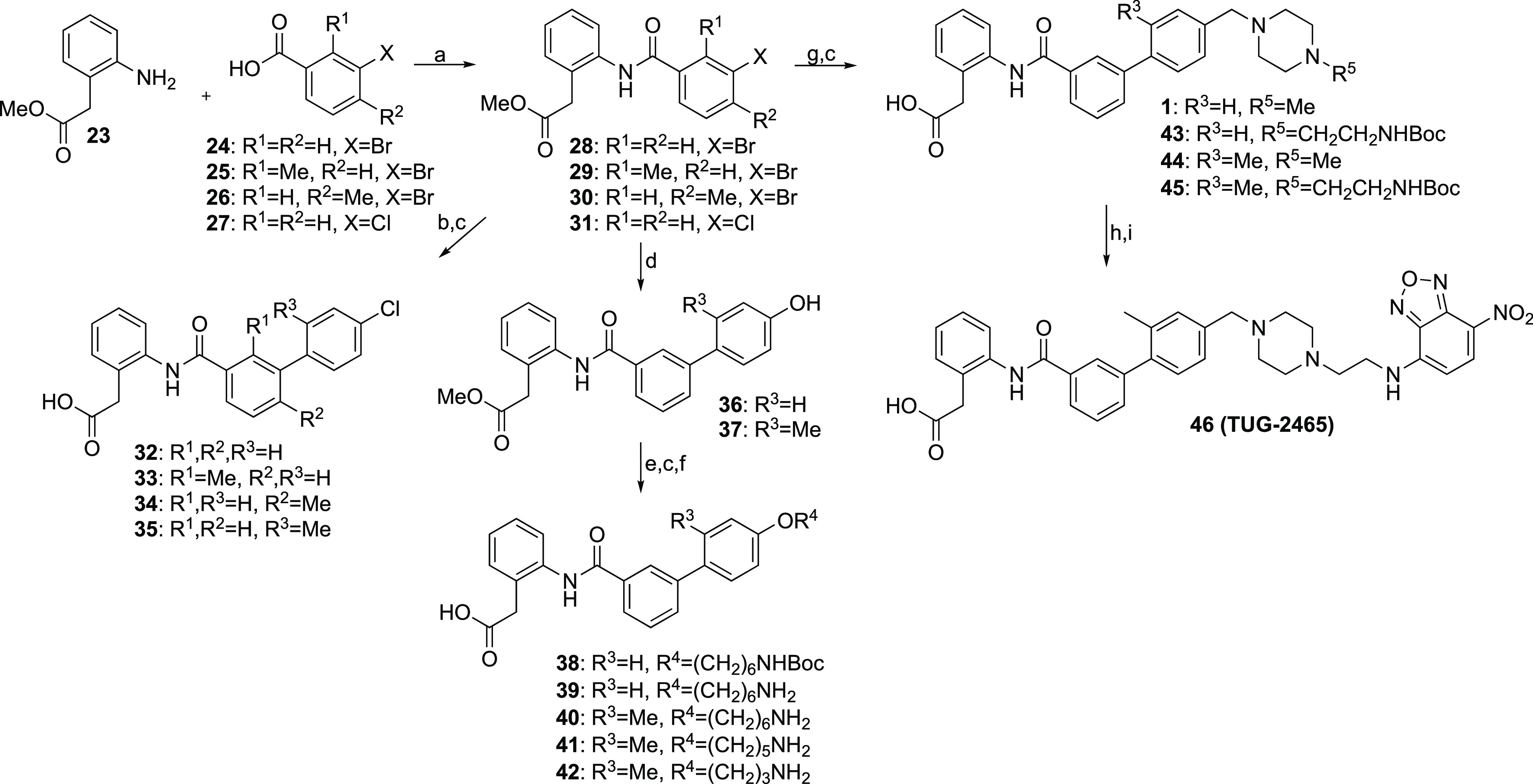
Synthesis of Antagonist Tracer Precursors and the Antagonist Tracer **46** Reagents and conditions: (a) HATU, DIPEA, DMF, rt, 3–41 h, 40–97%; (b) 4-chlorophenylboronic acid or (4-chloro-2-methylphenyl)boronic acid, PdCl_2_(PPh_3_)_2_, 1 M Na_2_CO_3_ (aq), EtOH or MeOH, toluene, 70–80 °C, 17–20 h, 27–70%; (c) 0.6 M LiOH (aq), THF, rt, 2–14 h, 45–100%; (d) 4-hydroxyphenylboronic acid or (4-hydroxy-2-methylphenyl)boronic acid, Pd-XPhos-G4, 0.5 M K_3_PO_4_ (aq), THF, rt–50 °C, 19–22 h, 70–75%; (e) alkyl halide/tosylate, K_2_CO_3_, (KI), MeCN or DMF, 50–90 °C, 22–43 h, 43–57%; (f) 4 M HCl (aq), THF, 55 °C, 15 h, 67% or 4 M HCl in dioxane, rt, 17–23 h, 71–100%; (g) 1) BBA, XPhos-Pd-G2, XPhos, KOAc, EtOH, 80 °C, 1–2.5 h; 2) aryl halide, 1.8 M K_2_CO_3_ (aq), 80 °C, 18–22 h, 24–75%; (h) TFA, DCM, rt, 3 h, 100%; (i) NBD-Cl, MeOH, Et_3_N, rt, 29 h, 30%.

The antagonists were synthesized following the same overall synthetic strategy starting from the methyl ester of 2-(2-aminophenyl)acetic acid (**23**) that underwent HATU-mediated amide coupling with halogen-substituted benzoic acid derivatives (Scheme 3). These amide intermediates were converted to various biphenyl intermediates by Suzuki coupling either directly using boronic acids or by a two-step, one-pot protocol forming first the boronic ester *in situ* from the first aryl halide followed by Suzuki coupling to a second aryl halide. The *para*-chloro analogues were directly hydrolyzed to give the final antagonists. The alkoxy analogues were prepared from the phenol intermediates **36** and **37**, first by alkylation with various Boc-protected aminoalkyl halide and tosylates, followed by basic ester hydrolysis, and finally Boc-deprotection using TFA. The piperazine analogues were hydrolyzed after the Suzuki coupling, and **46** was prepared from **45** by a Boc-deprotection followed by a substitution reaction with NBD-Cl.

First, we set out to investigate five different linkers on the mouse agonist scaffold **2** (Table 1). Simple aliphatic linkers were chosen to investigate the optimal linker length, including one PEG-based linker to examine effects of polarity, all containing a Boc-protected amine as a stable surrogate for the fluorophore that to some degree mimics the steric bulk but at the same time is a handle for easy introduction of the NBD-fluorophore after deprotection. Unfortunately, all linkers seemed to reduce the potency on human and mouse SUCNR1, with the C5-linker analogue **8c** being slightly better tolerated, showing only a 2.5-fold decrease in potency on mSUCNR1. Exploring the same five linkers on the agonist with selectivity for hSUCNR1 (**3**) led to a dramatic decrease in potency for all compounds (**15a**–**e**) on hSUCNR1 while only affecting the potency slightly on mSUCNR1. Based on this, we selected **8c** as the best tracer precursor and converted the compound to the corresponding NBD-tracer **7**, which fully sustained the potency on mSUCNR1 (pEC_50_ = 6.70 ± 0.16) but showed poor potency on hSUCNR1 (pEC_50_ = 5.84 ± 0.31).

**Table 1:**
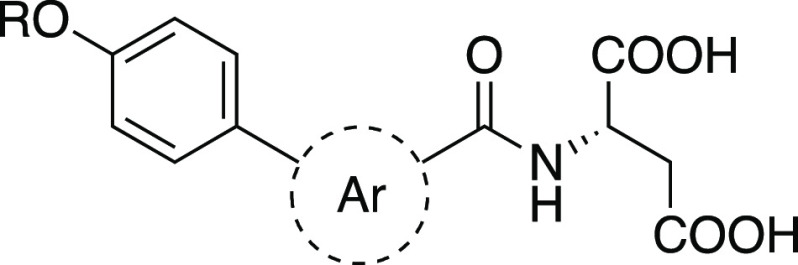

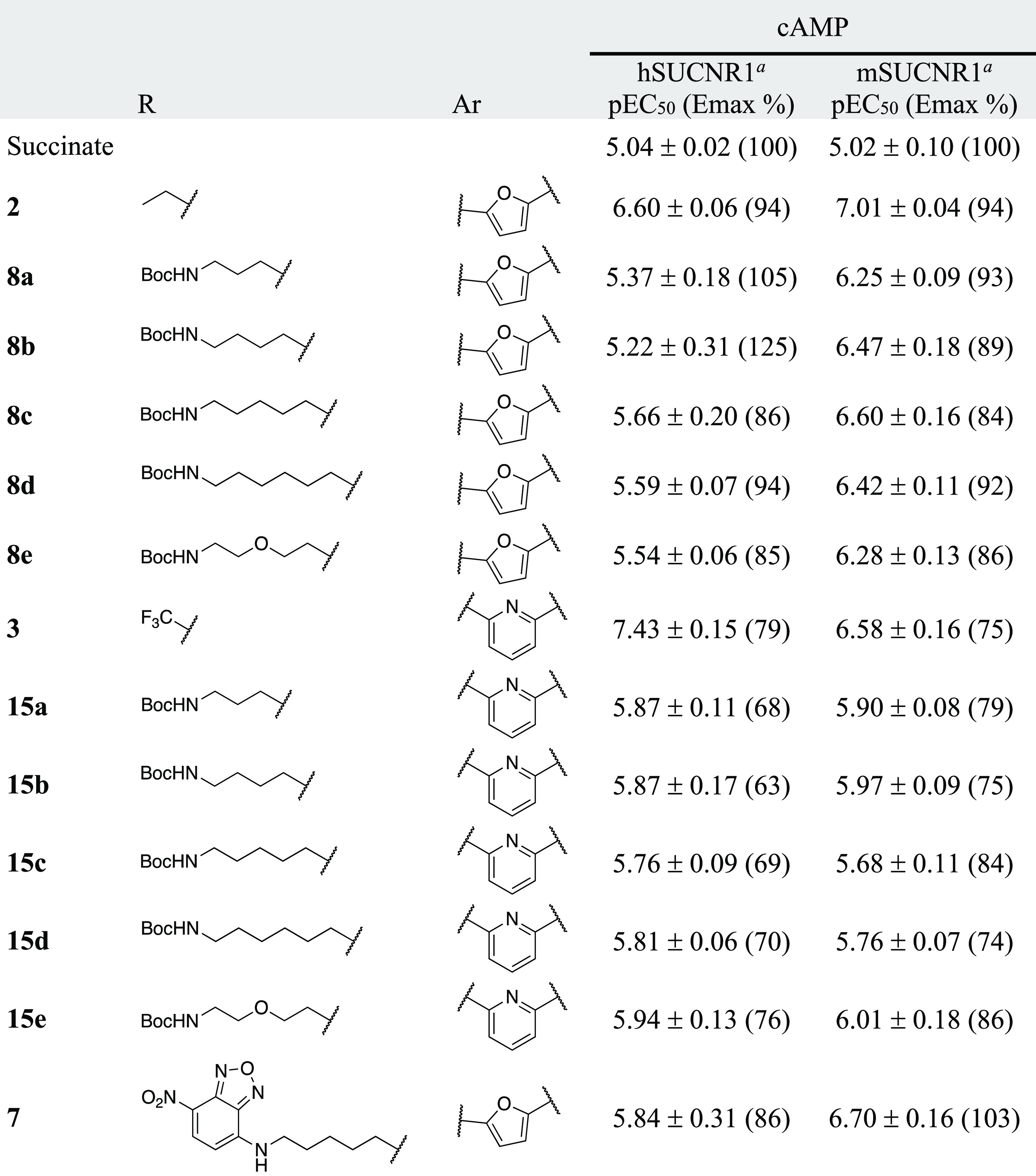
Potency of the First Agonist Tracer Precursors and Agonist Tracer **7**

a pEC_50_ data are from cAMP assays showing mean ± SEM from a minimum of three independent experiments.

**Figure 2:**
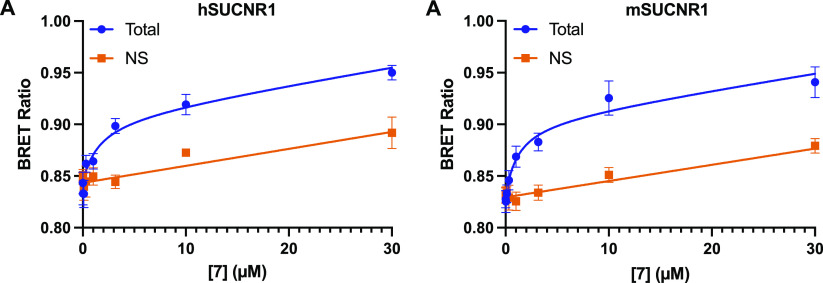
**7** is a modest affinity agonist tracer for hSUCNR1 and mSUCNR1. BRET saturation binding for **7** using N-terminally Nluc-tagged hSUCNR1 (A) and mSUCNR1 (B). Non-specific (NS) binding was obtained by treating cells with 100 μM **17**. Data are shown as mean ± SEM from three independent experiments carried out in duplicate. Data were globally fit to a total and non-specific binding equation and yielded *K*_d_ values of 1.58 μM (95% CI = 0.47–4.98 μM) for hSUCNR1 and 1.16 μM (95% CI = 0.47–4.97 μM) for mSUCNR1.

Despite its relatively low potency for hSUCNR1, we next tested whether **7** may still be a useful agonist fluorescent tracer for either human or mouse SUCNR1 in BRET-based binding assays. For this assay, versions of both human and mouse SUCNR1 were tagged at their extracellular N termini with Nluc and expressed in Flp-In T-REx 293 cells. To confirm that these Nluc-modified receptors did not have altered pharmacology, we first compared the expression level of the Nluc-hSUCNR1 and Nluc-mSUCNR1 constructs using their Nluc emission, finding no statistical difference in expression levels between the two orthologs (mSUCNR1 expression was 85 ± 10% of hSUCNR1 expression). We next demonstrated that succinate activated both Nluc-mSUCNR1 (pEC_50_ = 5.69 ± 0.11) and Nluc-hSUCNR1 (pEC_50_ = 4.80 ± 0.01) in a cAMP assay with comparable potency to what we observed previously using the unmodified receptors. Likewise, **7** showed high potency at Nluc-mSUCNR1 (pEC_50_ = 7.07 ± 0.07) but significantly reduced potency at Nluc-hSUCNR1 (pEC_50_ = 5.94 ± 0.18), in excellent alignment with our initial results for **7** using unmodified SUCNR1 constructs. When tested in saturation BRET binding assays, **7** demonstrated clear specific binding to both hSUCNR1 (Figure 2A, Figure S1A) and mSUCNR1 (Figure 2B, Figure S1B). Surprisingly, although the affinity of **7** for mSUCNR1 (*K*_d_ = 1.16 μM) was higher than it was for hSUCNR1 (*K*_d_ = 1.58 μM), this difference was not significant and was less pronounced than might have been expected based on the 7–13-fold higher potency this compound displays for mSUCNR1 over hSUCNR1 in cAMP assays. Interestingly, the measured affinity of **7** for hSUCNR1 matches very well with the potency of this compound in the cAMP assay at the human receptor, suggesting that for hSUCNR1 there is no receptor reserve operating in **7** cAMP signaling. Together, these results suggest that, despite having similar affinities at hSUCNR1 and mSUCNR1, the greater potency of this compound for mSUCNR1 is likely derived from differences in coupling efficiency of human and mouse SUCNR1 for the cAMP pathway.

**Figure 3:**
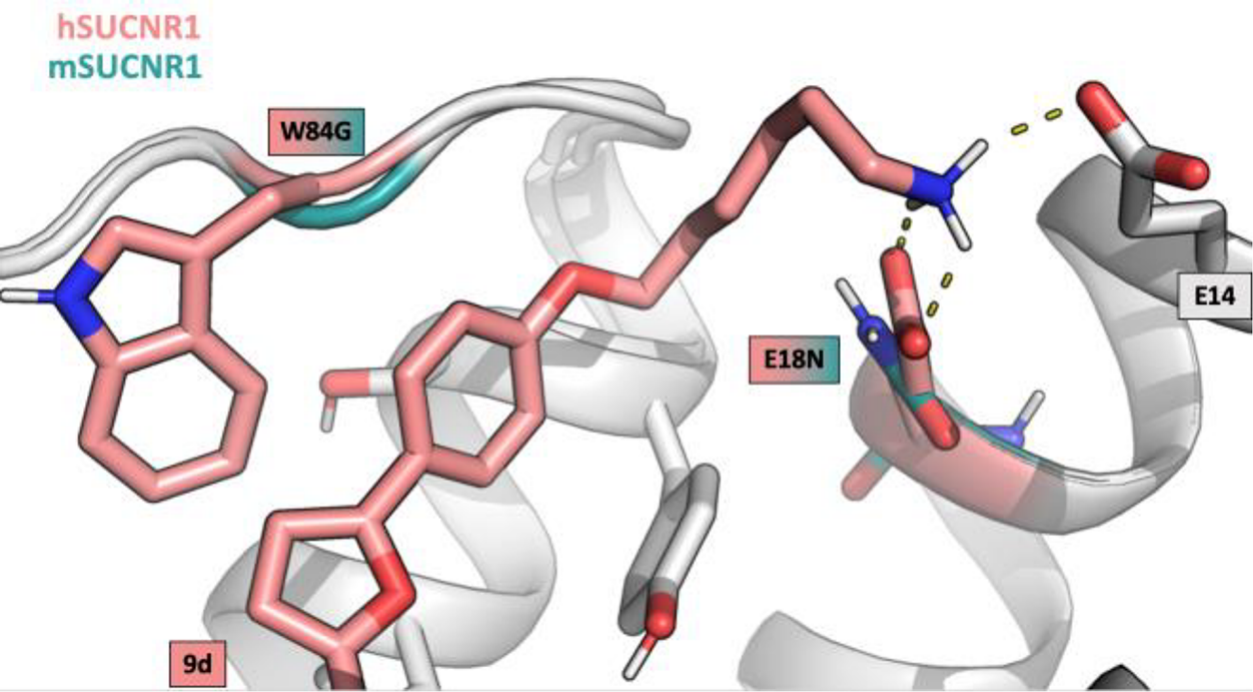
**9d** in complex with h- (salmon) and overlaid with mSUCNR1 (petrol) receptor model. Helixes and conserved residues are in gray.

Considering the relatively poor potency for **7** at hSUCNR1, we next used computational modeling to examine the potential binding mode of the agonist tracer precursors. Knowing that the antagonist **1** can displace the agonist tracer **7** and that the compounds share many common structural features, including an acidic headgroup connected by an amide to a biphenyl core, we used the X-ray structure of **1** in complex with the humanized rSUCNR1^27^ as template and used induced-fit docking to better compensate for structural differences between the active and inactive states of the receptor. Based on these results, we hypothesized that a positive charge in the linker could gain additional ionic interactions with Glu18 in hSUCNR1 and Glu14 conserved in human and mouse SUCNR1 and thereby increase affinity (Figure 3). To investigate this, we Boc-deprotected all the furane-based tracer precursors with alkylene linkers and tested the tracer precursors with free amines (**9a**–**d**, Table 2). To our satisfaction, all compounds increased in potency on both receptor orthologs, with a significant preference for the longer **9d** for hSUCNR1 and marginally increased potency on mSUCNR1.

**Table 2:**
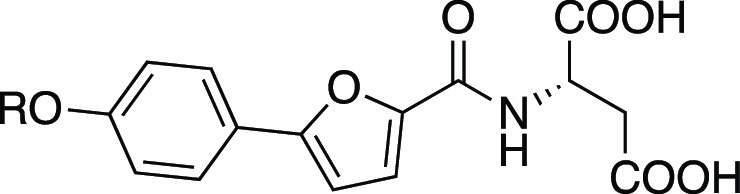

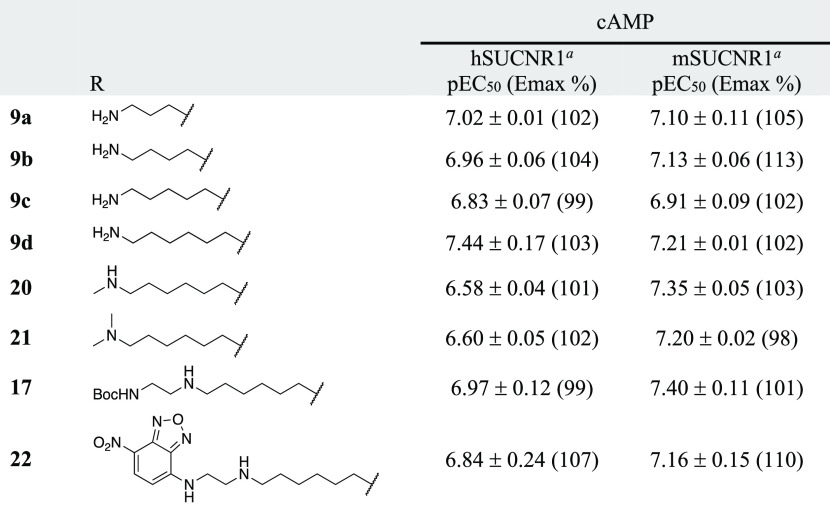
Potency of the Optimized Agonist Tracer Precursors and Agonist Tracer **22**

a pEC_50_ data presented are from cAMP assays and show the mean ± SEM from a minimum of three independent experiments.

To avoid introduction of a second stereocenter in the ligands, we aimed at extending the linker at the terminal amine to thereby have a positive charge as either a secondary or tertiary amine rather than incorporating the primary amine on the linker. To make sure this was a viable strategy, we first made the corresponding *N*-methyl (**20**) and *N*,*N*-dimethyl (**21**) analogues. Both compounds showed preserved potency on mSUCNR1 and a small but acceptable decrease on hSUNCR1. We therefore decided to extend the secondary amine further to accommodate the NHBoc handle for introduction of the NBD-fluorophore. The tracer precursor **17** nicely regained some of the lost potency on hSUCNR1 and showed nanomolar potency on both receptor orthologs (pEC_50_ = 6.97–7.40). Thus, the corresponding NBD-tracer **22** was synthesized, and although the potency decreased slightly on both orthologs (pEC_50_ = 6.84–7.16), the compound represents a SUCNR1 tracer with improved potency for both the hSUCNR1 and mSUCNR1.

**Figure 4:**
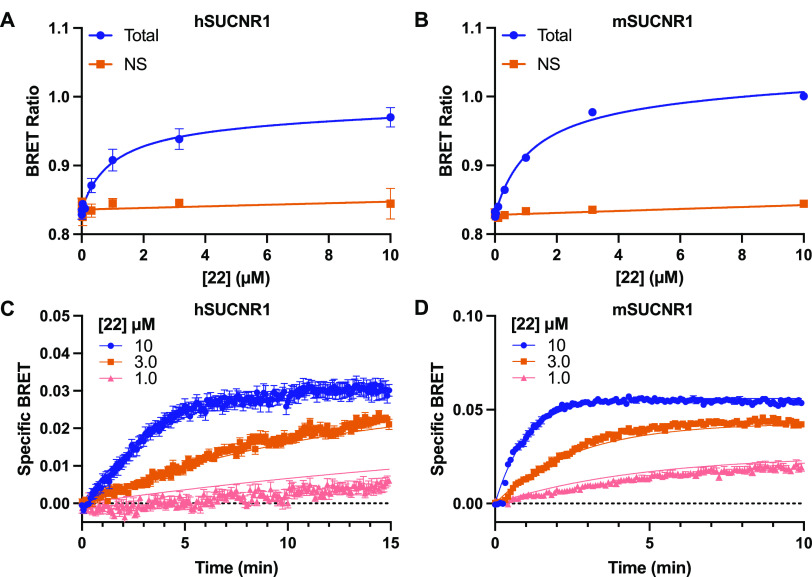
**22** is a fluorescent agonist tracer for both human and mouse SUCNR1. BRET saturation binding for **22** using N-terminally Nluc-tagged hSUCNR1 (A) and mSUCNR1 (B). Non-specific (NS) binding was obtained by treating cells with 100 μM **17**. Saturation binding data are shown as mean ± SEM from three independent experiments carried out in duplicate. Saturation binding data were globally fit to a total and non-specific binding equation, yielding a *K*_d_ value of 0.99 μM (95% CI = 0.45–2.15 μM) for hSUCNR1 and 1.17 μM (95% CI = 0.90–1.51 μM) for mSUCNR1. Kinetic BRET binding experiments are shown for Nluc-hSUCNR1 (C) and Nluc-mSUCNR1 (D). The kinetic data are shown as specific BRET, subtracting signal obtained when treating with equivalent concentrations of **22** in the presence of 100 μM **17**. Kinetic binding data are shown as mean ± SEM from three independent experiments carried out in triplicate and were globally fit to an equation for binding of multiple concentrations of labeled ligand.

Before testing **22** in BRET binding assays, we first confirmed that its activity was retained at Nluc-tagged hSUCNR1 (pEC_50_ = 7.38 ± 0.08) and mSUCNR1 (pEC_50_ = 7.41 ± 0.06). Testing **22** in saturation BRET binding assays demonstrated that this compound displays specific binding to both hSUCNR1 (Figure 4A, Figure S2A) and mSUCNR1 (Figure 4B, Figure S2B), with *K*_d_ values of 990 and 1170 nM, respectively. These results show that **22** has comparable affinity to our first tracer, **7**, at mSUCNR1, but with a modest improvement in affinity for hSUCNR1. However, improvement in affinity when comparing **22** to **7** was much less pronounced (1.6-fold) than the improvement in cAMP potency (10–14-fold) between the compounds, perhaps suggesting that the amine in **22** has a greater effect on signal transduction efficiency than it has on binding affinity.

We next aimed to assess whether **22** could be used in real-time binding kinetic assays. Experiments were conducted by measuring the association binding of multiple concentrations of **22** and globally fitting these data to a single equation in order to provide estimates of both on (*k*_on_) and off (*k*_off_) rates, as well as the dissociation constant (*K*_d_). Assessment of the binding kinetics of **22** at hSUCNR1 resulted in a *k*_on_ of 23100 M^–1^ min^–1^ (95% CI = 22000–24000 M^–1^ min^–1^) and *k*_off_ of 0.014 min^–1^ (95% CI = 0.008–0.021 min^–1^) (Figure 4C). Deriving *K*_d_ from these kinetic studies yields a value of 620 nM, in good alignment with the affinity measured for **22** at hSUCNR1 in the saturation binding assay (Figure 4A). Kinetic analysis of **22** binding to mSUCNR1 yielded a *k*_on_ of 85800 M^–1^ min^–1^ (95% CI = 84000–88000 M^–1^ min^–1^) and *k*_off_ of 0.13 min^–1^ (95% CI = 0.12–0.14 min^–1^), suggesting substantially increased on and off rates for the ligand at mSUCNR1 compared to hSUCNR1. Importantly, when deriving the *K*_d_ from the on and off rates for mSUCNR1, a value was obtained (1500 nM) that was still in close alignment with the *K*_d_ measured in the saturation binding assays (Figure 4B). To further explore the differences in binding kinetics of **22** between hSUCNR1 and mSUCNR1, residence times were calculated as 71 and 7.7 min for the two orthologs, respectively. As it was surprising to see such a large difference in residence times between the two species orthologs, we independently confirmed these results in more traditional dissociation rate experiments (Figure S2C,D), which yielded *k*_off_ rates of 0.057 min^–1^ and 0.32 min^–1^ for hSUCNR1 and mSUCNR1, respectively, both in good agreement with the values derived from our multiple concentration association kinetic studies.

Having now identified a fluorescent SUCNR1 agonist tracer, we next set out to develop a comparable antagonist tracer compound. Besides being complementary tools, antagonist tracers have a number of advantages over agonist tracers; e.g., they do not cause receptor desensitization or internalization. Since all current SUCNR1 antagonists are human specific, we focused our optimization only on hSUCNR1. First, we investigated if pre-arranging the biphenyl core of PB-20-OV24 (**32**) in a twisted conformation would improve the potency. We explored addition of methyl groups to three different *ortho*-positions of **32** (Table 3). Having a methyl in the R^1^-position (**33**) led to more than a 2-fold decrease in potency, indicating space limitations in this direction in the binding pocket, while moving the methyl to the R^2^-position (**34**) increased the potency 3-fold. Adding the methyl to the second ring in the R^3^-position (**35**) proved the most successful and increased the potency almost 15-fold, thereby confirming our hypothesis. Next, alkoxyamines were explored, similar to the agonist series; however, surprisingly, compounds with NHBoc, represented here by **38**, showed low-potency agonist activity rather than antagonism, and we therefore decided to explore the unprotected alkoxyamines instead. On these compounds, the R^3^-methyl surprisingly had the opposite effect on the longer C6-chain, leaving the non-methylated analogue **39** slightly more potent than **40**. However, decreasing the linker length to C5 (**41**) and C3 (**42**) increased the potency of the R^3^-methylated scaffold, almost regaining the potency of the chlorinated analogue **35**. Since having a free amine seemed to be essential for having antagonistic activity on these extended compounds, we turned our attention back to the published antagonist **1** to investigate if the piperazine could be extended with a small linker for attachment of the fluorophore and in that way keep the piperazine amines to maintain antagonistic behavior. Indeed, this was possible, and extension with *tert*-butyl methylcarbamate (**43**) preserved the activity. We also wanted to see if addition of the R^3^-methyl to **1** would affect the potency and found a 4-fold increase in potency for **44**, while the extended analogue **45** was only slightly more potent than the corresponding non-methylated analogue **43**. Finally, **45** was selected as the best tracer precursor, and introduction of NBD (**46**) yielded a tracer with potency comparable to that of the precursor.

**Table 3:**
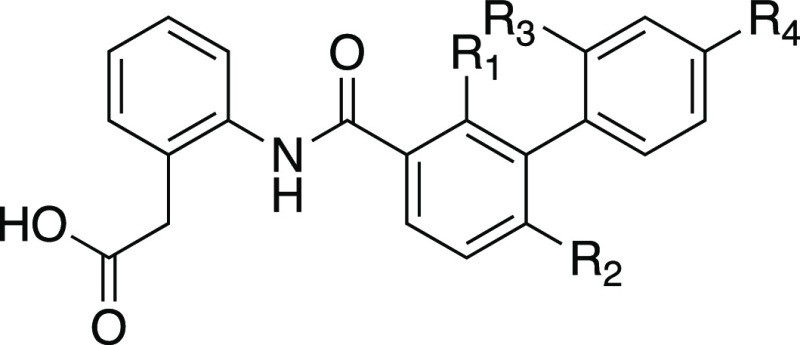

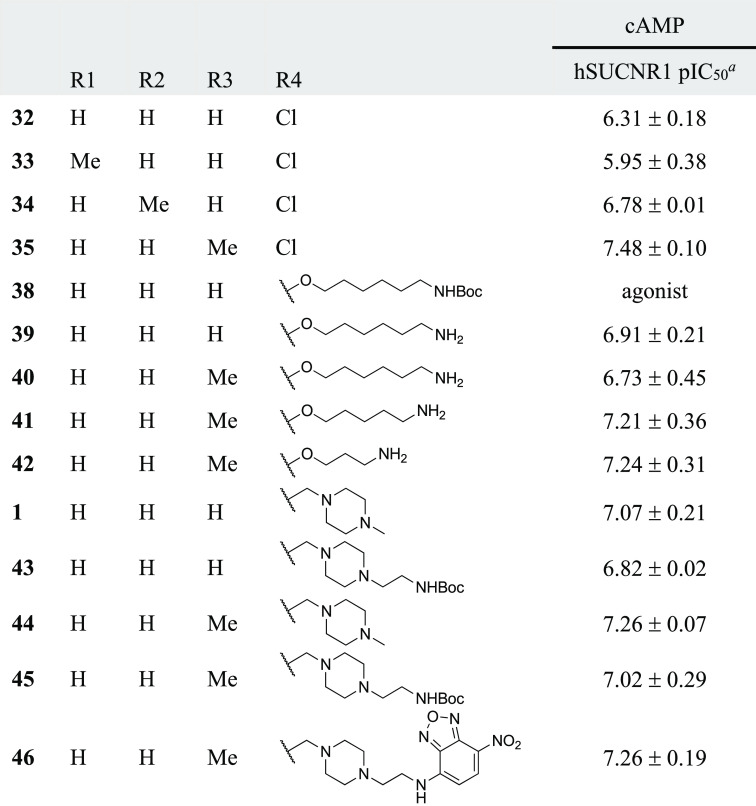
Potency of the Antagonist Tracer Precursors and Tracer **46**

a pIC_50_ data presented are from cAMP assays measuring inhibition of 31.6 μM succinate response and are the mean ± SEM from a minimum of three independent experiments.

**Figure 5:**
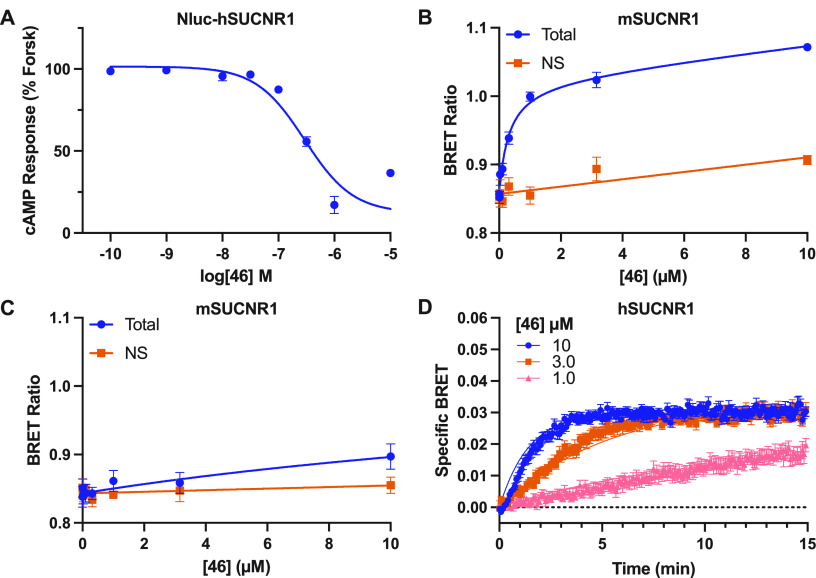
**46** is a fluorescent antagonist tracer for human but not mouse SUCNR1. **46** retains high-potency antagonism in the cAMP assay for the Nluc-hSUCNR1 construct (A). BRET saturation binding for **46** using N-terminally Nluc-tagged hSUCNR1 (B) and mSUCNR1 (C). Non-specific (NS) binding was obtained by treating cells with 100 μM **17**. Saturation binding data are shown as mean ± SEM from three independent experiments in duplicate and globally fit to a total and non-specific binding equation, yielding a *K*_d_ value of 300 nM (95% CI = 178–528 nM) at hSUCNR1. Kinetic BRET binding experiments are shown for Nluc-hSUCNR1 (D). Kinetic data are shown as specific BRET after subtracting the signal obtained when treating with the equivalent concentrations of **46** in the presence of 100 μM **17**. Kinetic binding data are mean ± SEM from three independent experiments in triplicate and were globally fit to an equation for binding of multiple concentrations of labeled ligand.

We next aimed to confirm that **46** retained activity at the Nluc-hSUCNR1 receptor that would be used for the BRET binding assay, observing a pIC_50_ of 6.75 ± 0.04 (Figure 5A). In these experiments it was noted that, at high (10 μM) concentrations, **46** tended to not fully inhibit the cAMP response; however, **46** is a highly colored fluorescent compound, and it is likely that this is an artifact resulting from these properties. Saturation BRET binding experiments with **46** demonstrated clear specific binding to hSUCNR1, with a *K*_d_ of 310 nM (Figure 5B, Figure S3A). Not surprisingly, given the lack of ability of **46** as well as all other SUCNR1 antagonists we have tested to antagonize mSUCNR1 in cAMP assays, when tested in a saturation binding assay at mSUCNR1, **46** showed almost no specific binding (Figure 5C). Kinetic analysis of **46** binding using hSUCNR1 produced *k*_on_ = 61800 M^–1^ min^–1^ and *k*_off_ = 0.035 min^–1^, yielding an estimated *K*_d_ value of 560 nM (Figure 5D), in nice alignment with the saturation binding data (Figure 5A). This off rate was independently verified using a traditional dissociation kinetic experiment, yielding a highly comparable *K*_off_ value of 0.034 min^–1^ (Figure S3B). Together, these data suggest that **46** is a useful antagonist fluorescent tracer for hSUCNR1 but not for mSUCNR1.

Next, we aimed to use **46** to understand why the antagonists are potent at hSUCNR1 but inactive at mSUCNR1. Previous work has identified two humanizing mutations in the rat (r) ortholog of SUCNR1, K18^1.31^E and K269^7.32^N, that are required to gain antagonist binding to rSUCNR1.^27^ In mSUCNR1, these residues are N18^1.31^ and K269^7.32^; we therefore generated an N18^1.31^E/K269^7.32^N mSUCNR1 construct to establish if these mutations restore antagonist function. To avoid the need to make a stable cell line expressing this mutant receptor, we used a BRET-based assay that measures direct activation of Gα_i2_^36^ in transiently transfected HEK-293T cells. Importantly, we first demonstrated that the mSUCNR1-N18^1.31^E/K269^7.32^N mutant retains the ability to respond to succinate in our G protein activation assay (Figure S4A). Interestingly, unlike in our cAMP assay, in the G protein activation assay we observe somewhat higher potency for succinate at mSUCNR1 (pEC_50_ = 5.29 ± 0.12) than hSUCNR1 (pEC_50_ = 4.45 ± 0.23), consistent with previous reports that succinate is more potent at mSUCNR1 than hSUCNR1.^13^ The pEC_50_ of succinate at mSUCNR1-N18^1.31^E/K269^7.32^N in this assay was 4.76 ± 0.11, suggesting that these mutations may in part account for differences in succinate potency between human and mouse SUCNR1. However, when tested for antagonism against an EC_80_ concentration of succinate, **1** still showed no measurable antagonism against mSUCNR1-N18^1.31^E/K269^7.32^N (Figure S4B).

**Figure 6:**
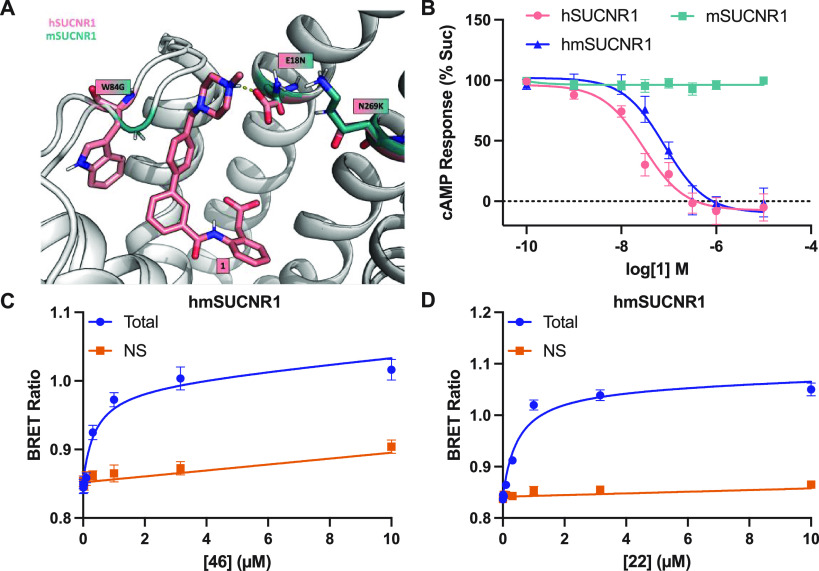
Humanizing mutations N18^1.31^E, K269^7.32^N, and G84^EL1^W allow for antagonist binding to mSUCNR1. **1** in complex hSUCNR1 (salmon) and overlay with mSUCNR1 (petrol) highlighting the three central amino acids for species selectivity (A). Recovery of antagonist function at hmSUCNR1 was confirmed using a cAMP assay measuring inhibition of an EC_80_ concentration of succinate with **1** at hSUCNR1, mSUCNR1, and hmSUCNR1 (N18^1.31^E, K269^7.32^N, and G84^EL1^W) (B). Data in cAMP experiments are mean ± SEM of three independent experiments in triplicate. Saturation binding results are shown for hmSUCNR1 using **46** (C) or **22** (D). Non-specific binding (NS) was measured using 100 μM **17**. Saturation binding data are shown as mean ± SEM from three independent experiments completed in triplicate. Saturation binding data were globally fit to a total and non-specific binding equation yielding a *K*_d_ value for **22** of 490 nM (95% CI = 370–640 nM) and for **46** of 390 nM (95% CI = 230–650 nM).

This led us to further explore differences between rat, mouse, and human SUCNR1 to determine why mutation of these sites is sufficient for antagonist activity at rSUCNR1 but not mSUCNR1. Critically, when examining the residues shown to be involved in antagonist binding in the SUCNR1 crystal structure,^27^ only one residue was identified that is conserved in human (W88^EL1^) and rat (W84^EL1^) but not mouse (G84^EL1^) SUCNR1 (Figure 6A). We therefore generated an additional mutant mSUCNR1 construct containing N18^1.31^E, K269^7.32^N, and G84^EL1^W and first demonstrated in our G protein activation assay that this mutant is activated by succinate with a pEC_50_ of 4.28 ± 0.09 (Figure S4A). Satisfyingly, when we tested **1** as an antagonist against an EC_80_ concentration of succinate at this mutant, a level of antagonism similar to that with hSUCNR1 was observed (Figure S4B). Together, this suggests that these three residues are critical for the lack of activity of antagonists at mSUCNR1 and that incorporation of N18^1.31^E, K269^7.32^N, and G84^EL1^W mutations is sufficient to produce a humanized (hm) version of mSUCNR1. We therefore established a hmSUCNR1 cell line and used this to confirm that **1** potently inhibits hmSUCNR1 in our cAMP assay (Figure 6B). Indeed, in line with what was observed in the G protein activation assay, **1** inhibited succinate response at hmSUCNR1 to a similar degree to how it inhibits hSUCNR1, with a pIC_50_ of 7.10 ± 0.10. Having demonstrated functional antagonism at hmSUCNR1, we used our fluorescent tracer compound to assess antagonist binding to hmSUCNR1. In line with our cAMP results, clear specific binding was observed for the tracer antagonist, **46**, with a *K*_d_ of 390 nM (Figure 6C, Figure S5A). This affinity is very similar to the *K*_d_ obtained for **46** at hSUCNR1 (310 nM), suggesting that these three residues fully account for the lack of antagonist binding to mSUCNR1. In addition to binding the tracer antagonist, hmSUCNR1 also broadly maintains agonist binding, as both agonist tracers, **22** (*K*_d_ = 490 nM; Figure 6D, Figure S5B) and **7** (*K*_d_ = 2650 nM; Figure S5C) bind effectively to hmSUCNR1.

**Figure 7:**
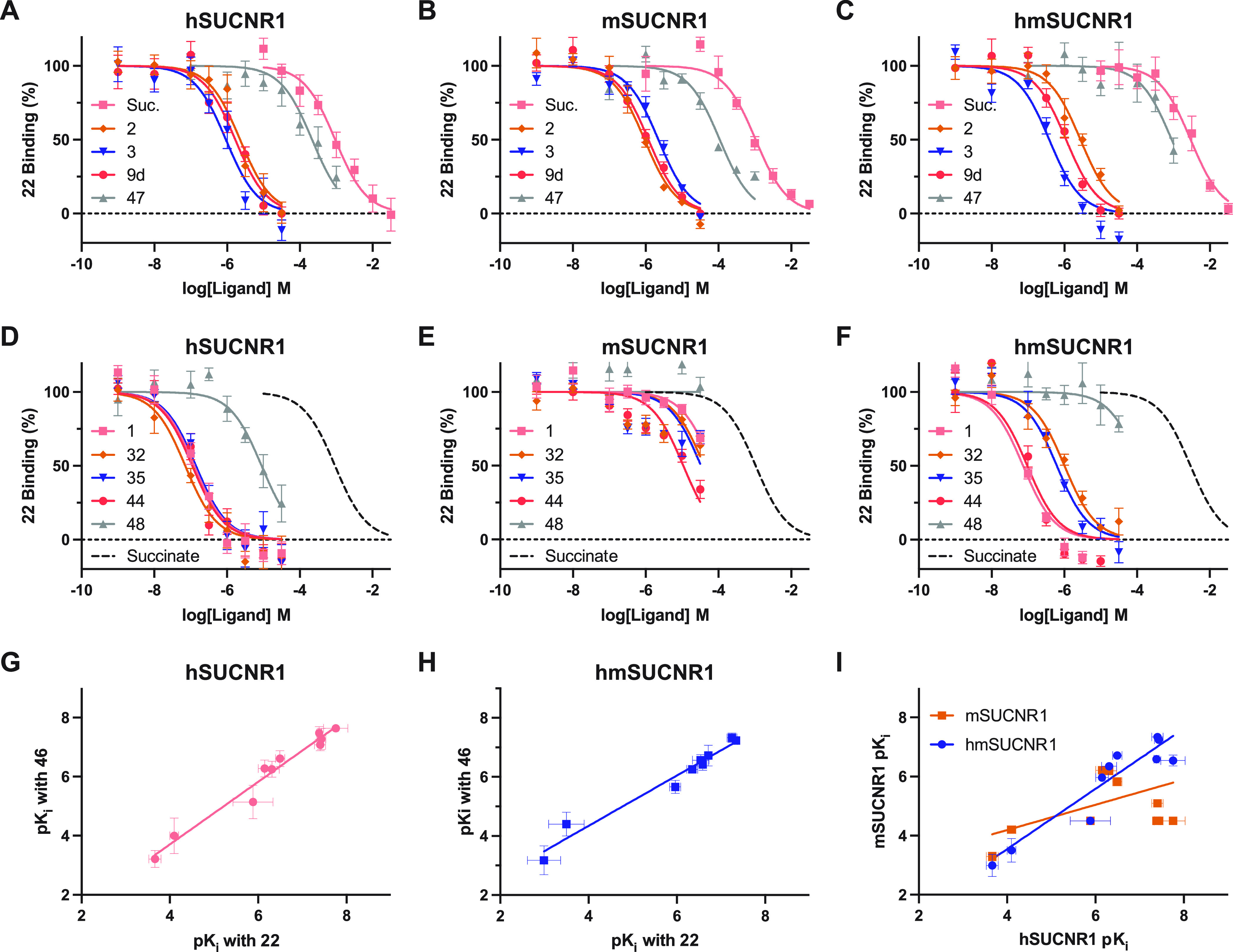
Humanizing mutations N18^1.31^E, K269^7.32^N, and G84^EL1^W transform mSUCNR1 pharmacology to match that of hSUCNR1. Competition binding experiments were conducted for various SUCNR1 agonists using **22** as the tracer ligand at hSUCNR1 (3 μM **22**) (A), mSUCNR1 (1 μM **22**) (B), and hmSUCNR1 (1 μM **22**) (C). Comparable experiments were conducted using **22** as the tracer for various SUCNR1 antagonists at the same three receptor constructs with their succinate competition curve shown for reference (D–F). All competition binding data are shown as mean ± SEM from three independent experiments conducted in duplicate. The p*K*_i_ affinity values obtained at hSUCNR1 (G) or hmSUCNR1 (H) in competition binding experiments using **22** as the tracer ligand are correlated with the values obtained using **46** as the tracer for the same set of competing ligands. A correlation for p*K*_i_ values obtained with **22** at hSUCNR1 against the values obtained with the same ligands at either mSUCNR1 or hmSUCNR1 (I).

Having identified a tracer agonist for human and mouse SUCNR1 and a tracer antagonist for human and humanized mouse SUCNR1, we set out to use these compounds to assess binding affinity of various unlabeled SUCNR1 ligands. Ten SUCNR1 ligands were chosen and tested in competition binding assays using concentrations of **22** tracer comparable to its calculated *K*_d_ at hSUCNR1 (3 μM), mSUCNR1 (1 μM), or hmSUCNR1 (1 μM) (Table 4), including previously published *cis*-epoxysuccinic acid (**47**) and an example antagonist from the patent literature^37^ (**48**). Across the testing we consistently observed that all agonists displayed comparable binding to all three SUCNR1 receptors (Figure 7A–C). However, as predicted, most antagonists tested bound with high affinity only to hSUCNR1 and hmSUCNR1 (Figure 7D–F). Next, to compare the competition binding data obtained using our agonist vs antagonist tracer, we tested the same set of compounds in competition binding assays using **46** as the tracer (562 nM) at hSUCNR1 and hmSUCNR1 (Table 4). An excellent correlation was observed between the p*K*_i_ values obtained using the two different fluorescent ligands at both hSUCNR1 (Figure 7G; *r* = 0.98) and hmSUCNR1 (Figure 7H; *r* = 0.98), confirming that both the agonist and antagonist tracer ligands are binding competitively to the same site of SUCNR1. In addition, the affinities across all compounds tested at hmSUCNR1 correlated much better with the affinities for these compounds at hSUCNR1 (r = 0.94) than the affinities obtained for these compounds at mSUCNR1 (Figure 7I; *r* = 0.39)

**Table 4:**
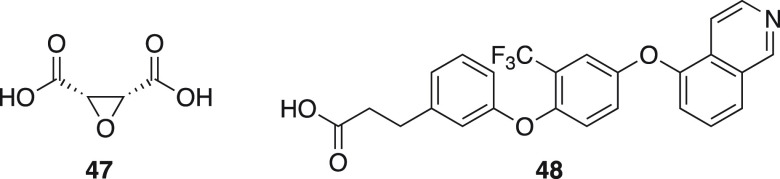
p*K*_i_ Values at hSUCNR1, mSUCNR1, and hmSUCNR1 Obtained in Competition Binding Studies

	Competion binding using **22**^a^^,^^b^			Competion binding using **46**^a^^,^^c^	
	hSUCNR1 p*K*_i_	mSUCNR1 p*K*_i_	hmSUCNR1 p*K*_i_	hSUCNR1 p*K*_i_	hmSUCNR1 p*K*_i_
Succinate	3.7 ± 0.1	3.3 ± 0.1	3.0 ± 0.4	3.2 ± 0.1	3.2 ± 0.5
**1**	7.4 ± 0.1	5.1 ± 0.0	7.3 ± 0.0	7.1 ± 0.1	7.2 ± 0.1
**2**	6.1 ± 0.1	6.2 ± 0.1	6.0 ± 0.1	6.3 ± 0.1	5.7 ± 0.2
**3**	6.5 ± 0.1	5.8 ± 0.1	6.7 ± 0.0	6.6 ± 0.1	6.7 ± 0.3
**9d**	6.3 ± 0.2	6.2 ± 0.1	6.3 ± 0.1	6.3 ± 0.1	6.3 ± 0.1
**32**	7.8 ± 0.3	<4.5^d^	6.5 ± 0.2	7.6 ± 0.1	6.6 ± 0.2
**35**	7.4 ± 0.1	<4.5^d^	6.6 ± 0.0	7.5 ± 0.1	6.4 ± 0.2
**44**	7.4 ± 0.1	<4.5^d^	7.2 ± 0.1	7.3 ± 0.2	7.3 ± 0.2
**47**	4.1 ± 0.1	4.4 ± 0.1	3.5 ± 0.4	4.1 ± 0.3	3.5 ± 0.4
**48**	5.9 ± 0.5	<4.5^d^	<4.5^d^	5.1 + 0.3	<4.5^d^

a p*K*_i_ values are the mean ± SEM from three independent competition binding experiments.

b From competition binding experiments using **22** as the tracer at hSUCNR1, 3 μM **22**, *K*_d_ = 990 nM; mSUCNR1, 1 μM **22**, *K*_d_ = 1170 nM; and hmSUCNR1, 1 μM **22**, *K*_d_ = 486 nM.

c From competition binding experiments using **46** as the tracer at hSUCNR1, 0.562 μM **46**, *K*_d_ = 310 nM; and hmSUCNR1, 562 nM **46**, *K*_d_ = 390 nM.

d Competitive binding was not observed up to the highest concentration of unlabeled ligand tested (30 μΜ).

These competition binding studies established p*K*_i_ affinity values for succinate ranging from 3.0 to 3.7 across the three different receptor constructs. These data suggest a relatively low affinity of succinate for SUCNR1, suggesting that functional succinate responses rely on strong signal amplification and a receptor reserve to produce the higher pEC_50_ potencies (∼5.0) observed at both hSUCNR1 and mSUCNR1 in the cAMP assay. The same was true for the other agonists tested, for example **9d**, where the measured affinity (p*K*_i_ = 6.2–6.3) was notably lower than the potency for this compound in cAMP assays (pEC_50_ = 7.21–7.44). In contrast, antagonist affinities were comparable to, or slightly higher than, the IC_50_ values obtained in cAMP assays. For example, **1** yielded p*K*_i_ values between 7.10 and 7.4 for hSUCNR1, compared with an pIC_50_ value of 7.07 in the cAMP assay.

One interesting observation from our competition studies using hmSUCNR1 is that these humanizing mutations have not had the same effect across all antagonists. Of particular note, while antagonists related to **1** have activity completely restored in hmSUCNR1, **32** and **35** that lack the piperazine group show only a partial recovery of function, with the affinity remaining ∼10-fold lower at hmSUCNR1 compared to hSUCNR1. This cannot be explained by docking of **32** in homology models of h- or hmSUCNR1 where all residues in the binding pocket are conserved and the binding poses are nearly identical. Thus, differences outside the binding pocket must be responsible for the observed difference in affinity for the smaller antagonists at human and humanized mouse SUCNR1, and the ionic interaction between the piperazine and Glu18 on the larger antagonists fully compensates for this difference.

The patent derived antagonist **48**, which represents a different chemical scaffold, was found to be human specific and did not regain any activity on hmSUCNR1, but it could displace both the agonist and antagonist tracer, demonstrating that the compound binds to the same site as the others.

## Conclusions

We have developed the first potent fluorescent tracer agonist for SUCNR1, **22**. We have demonstrated that this tracer ligand can be used to measure both binding affinity and binding kinetics to SUCNR1 in living cells using a BRET-based assay. Interestingly, although our best tracer agonist, **22**, binds with comparable affinity to the human and mouse receptor orthologs, it does so with significantly different kinetic properties. Specifically, **22** has much faster association and dissociation rates, as well as a shorter residence time at mSUCNR1 compared to hSUCNR1. In addition, we have also developed the first fluorescent SUCNR1 antagonist, **46**, and used this ligand to demonstrate that three residues that differ between human and mouse SUCNR1 account for the lack of binding of currently known SUCNR1 antagonists to the mouse receptor. We further use both **22** and **46** in a range of competition binding studies to demonstrate that both ligands bind to the same site on SUCNR1. Together, these fluorescent tracers provide a new way to measure binding to SUCNR1 in intact living cells and are expected to be invaluable tools in drug discovery efforts targeting this receptor.

## Experimental Section

### General Remarks

All commercially available starting materials and solvents were used without further purification unless otherwise stated. DCM, THF, and DMF were dried using a Glass Contour Solvent System built by SG Water USA. MeCN and NEt_3_ were distilled over CaH_2_ and stored under activated 4 Å molecular sieves; MeOH and *N,N*-diisopropylethylamine were dried over 3 Å molecular sieves. TLC was performed on TLC silica gel 60 F254 plates and visualized at 254 and/or 365 nm. Purification by flash chromatography was carried out using silica gel 60 (0.040–0.063 mm, Merck). Purification by automated flash chromatography was performed on a RevelerisX2 Flash Chromatography System, Büchi. ^1^H and ^13^C spectra were recorded at 400 and 600 MHz, 101 and 151 MHz, respectively, on Bruker Avance III (400 MHz) or Bruker Avance III HD (600 MHz) instruments at 300 K and calibrated relative to residual solvent peaks. HPLC analysis was performed on an UltiMate HPLC system (Thermo Scientific) using a Gemini-NX C18 column (3 μm, 4.6 mm × 250 mm, 110 Å); gradient elution method A: 0 to 100% mobile phase B (MeCN–H_2_O–TFA 90:10:0.1) in mobile phase A (H_2_O–TFA 100:0.1) or method B: 50 to 100% mobile phase B (MeCN–H_2_O–TFA 90:10:0.1) in mobile phase A (H_2_O–TFA 100:0.1) over 10 min, flow rate 1 mL/min, UV–vis detection at 254 or 450 nm. Preparative HPLC purification was carried out on an UltiMate HPLC system (Thermo Scientific), mobile phase A (H_2_O–TFA 100:0.1), mobile phase B (MeCN–H_2_O–TFA 90:10:0.1) with individually optimized gradients with UV–vis detection at 254 nm and/or 450 nm. Optical rotations were measured on an Anton Paar MCP polarimeter (Anton Paar Cell 100 mm, CL. 0.01, Ø 5 mm). Mass spectrometry (MS) was performed on an Aquity UPLC instrument connected to an Aquity TUV detector and an Aquity Qdadetector; gradient elution: 100% mobile phase A (MeCN–H_2_O–HCOOH 5:95:0.1) to 100% mobile phase B (MeCN–HCOOH 100:0.1) over 5 min, flow rate 0.5 mL/min; or on an Agilent 6130 mass spectrometer using electron spray ionization (ESI) coupled to an Agilent 1200 HPLC system (ESI-LCMS); gradient elution: 100% mobile phase A (MeCN–H_2_O–HCOOH 5:95:0.1) to 100% mobile phase B (MeCN–HCOOH 100:0.1) over 5 min, flow rate 1 mL/min. High-resolution mass spectra (HRMS) were recorded on a QExactive Orbitrap mass spectrometer equipped with a SMALDI5 ion source. The sample was analyzed in the positive ion mode using a peak from the DHB matrix for internal mass calibration whereby a mass accuracy of 2 ppm or better was achieved. Purity was determined by HPLC and confirmed by inspection of NMR spectra. The purity of all test compounds was >95%.

### (5-(4-((5-((7-Nitrobenzo[*c*][1,2,5]oxadiazol-4-yl)amino)pentyl)oxy)phenyl)furan-2-carbonyl)-l-aspartic acid (7)

*tert*-Butyl (5-hydroxypentyl)carbamate: To a round-bottom flask containing a solution of (Boc)_2_O (1143 mg, 5.24 mmol) dissolved in DCM (7.4 mL) were added 5-aminopentan-1-ol (433 mg, 4.20 mmol) and triethylamine (1.6 mL, 11.50 mmol) at 0 °C. The solution was allowed to reach rt and stirred overnight. After completion, the reaction mixture was quenched by addition of 10% aqueous NH_4_Cl and extracted with EtOAc (×3). The organic phases were combined, washed with brine, dried over Na_2_SO_4_, filtered, and concentrated *in vacuo* to give the product as an amber oil that was used directly in the next step (*R*_f_ = 0.66 (10% MeOH in DCM)).

5-((*tert*-Butoxycarbonyl)amino)pentyl 4-methylbenzenesulfonate: *tert*-Butyl (5-hydroxypentyl)carbamate was dissolved in DCM (11 mL) in a flask under an argon atmosphere. The mixture was cooled on an ice–water bath before addition of tosyl chloride (1217 mg, 6.38 mmol) followed by pyridine (0.85 mL, 10.49 mmol). The reaction mixture was stirred at rt for 21 h. After completion, the reaction mixture was washed with aqueous 1 M HCl (×2). The aqueous phases were combined and re-extracted with DCM (×2). The organic phases were combined, washed with brine, dried over Na_2_SO_4_, filtered, and concentrated *in vacuo*. The residue was purified by flash column chromatography (SiO_2_, 0–30% EtOAc in *n*-heptane) to give 1072 mg (71% over two steps) of the product as a white gel-like solid: *R*_f_ = 0.15 (EtOAc:*n*-heptane, 1:3); ^1^H NMR (400 MHz, CDCl_3_) δ 7.82–7.75 (m, 2H), 7.35 (d, *J* = 8.0 Hz, 2H), 4.47 (br s, 1H), 4.02 (t, *J* = 6.4 Hz, 2H), 3.08–3.04 (m, 2H), 2.45 (s, 3H), 1.72–1.62 (m, 2H), 1.47–1.39 (m, 11H), 1.38–1.30 (m, 2H); ^13^C NMR (101 MHz, CDCl_3_) δ 156.1, 144.9, 133.3, 130.0, 128.0, 77.4, 70.5, 29.6, 28.7, 28.6, 22.8, 21.8; ESI-MS (method A) *m*/*z* 258.3 (M+H^+^-Boc). Spectra in accordance with reported data.^38^

5-((*tert*-Butoxycarbonyl)amino)pentyl 4-methylbenzenesulfonate (32 mg, 0.09 mmol) was dissolved in MeCN (0.6 mL) in a dry flask under an argon atmosphere. Then, **4**^2^ (20 mg, 0.06 mmol), K_2_CO_3_ (17 mg, 0.12 mmol), and MeCN (0.6 mL) were added to the flask. The reaction mixture was stirred at 80 °C for 2 days. After completion, water was added to the reaction mixture. The aqueous phase was extracted with EtOAc (×3). The organic phases were combined, washed with brine, dried over MgSO_4_, filtered, and concentrated *in vacuo*. The residue was purified by flash column chromatography (SiO_2_, 0–1% MeOH in DCM) to give 14 mg (44%) of **5c** as a yellow foam: *R*_f_ = 0.24 (5% MeOH in DCM); ^1^H NMR (400 MHz, CDCl_3_) δ 7.68–7.61 (m, 2H), 7.34 (d, *J* = 8.2 Hz, 1H), 7.19 (d, *J* = 3.6 Hz, 1H), 6.97–6.89 (m, 2H), 6.60 (d, *J* = 3.6 Hz, 1H), 5.10–5.01 (m, 1H), 4.54 (br s, 1H), 4.00 (t, *J* = 6.4 Hz, 2H), 3.81 (s, 3H), 3.73 (s, 3H), 3.20–3.10 (m, 3H), 3.03–2.93 (m, 1H), 1.82 (p, *J* = 6.6 Hz, 2H), 1.63–1.48 (m, 4H), 1.45 (s, 9H); ^13^C NMR (101 MHz, CDCl_3_) δ 171.8, 171.3, 159.8, 158.2, 156.3, 156.1, 145.8, 126.3, 122.5, 117.5, 115.0, 105.9, 68.0, 53.1, 52.2, 48.4, 36.4, 30.0, 29.0, 28.6, 23.5; ESI-MS *m*/*z* 433.4 (M-Boc+H^+^).

**5c** (33 mg, 0.06 mmol) was dissolved in 1,4-dioxane (0.26 mL), and 4 M HCl in 1,4-dioxane (0.13 mL) was added dropwise. The reaction mixture was stirred at rt for 17 h. Then, additional 4 M HCl in 1,4-dioxane (0.13 mL) was added dropwise and stirred at rt for 10 h. The solvent was concentrated *in vacuo* to give **6** as an off-white solid (27 mg, 94%) that was used directly in the next step.

A dry flask was charged with **6** (11 mg, 0.02 mmol), NBD-Cl (6 mg, 0.03 mmol), NEt_3_ (15 μL, 0.10 mmol), and MeOH (0.23 mL) under an argon atmosphere. The reaction mixture was stirred for 18 h at rt. After completion, water was added to the reaction mixture. The aqueous phase was extracted with EtOAc (×3). The organic phases were combined, washed with brine, dried over MgSO_4_, filtered, and concentrated *in vacuo*. The residue was purified by flash column chromatography (SiO_2_, EtOAc:*n*-heptane, 1:1 to 2:1) to give 6 mg (40%) of dimethyl (5-(4-((5-((7-nitrobenzo[*c*][1,2,5]oxadiazol-4-yl)amino)pentyl)oxy)phenyl)furan-2-carbonyl)-l-aspartate as a red solid: *R*_f_ = 0.30 (EtOAc:*n*-heptane, 2:1); ^1^H NMR (600 MHz, CDCl_3_) δ 8.49 (d, *J* = 8.6 Hz, 1H), 7.67–7.61 (m, 2H), 7.37–7.33 (m, 1H), 7.20 (d, *J* = 3.6 Hz, 1H), 6.95–6.90 (m, 2H), 6.61 (d, *J* = 3.6 Hz, 1H), 6.30–6.25 (m, 1H), 6.18 (d, *J* = 8.6 Hz, 1H), 4.05 (t, *J* = 6.0 Hz, 2H), 3.81 (s, 3H), 3.73 (s, 3H), 3.55 (q, *J* = 6.7 Hz, 2H), 3.20–3.11 (m, 1H), 3.04–2.96 (m, 1H), 1.97–1.87 (m, 4H), 1.75–1.67 (m, 2H); ^13^C NMR (151 MHz, CDCl_3_) δ 171.8, 171.4, 159.5, 158.1, 156.1, 145.8, 144.4, 144.0, 143.9, 136.5, 126.3, 124.4, 122.8, 117.5, 114.9, 106.0, 98.7, 67.6, 53.1, 52.3, 48.4, 44.0, 36.4, 28.9, 28.4, 23.9; ESI-MS *m*/*z* 596.2 (M+H^+^).

Dimethyl (5-(4-((5-((7-nitrobenzo[*c*][1,2,5]oxadiazol-4-yl)amino)pentyl)oxy)phenyl)furan-2-carbonyl)-l-aspartate (6 mg, 0.01 mmol) was dissolved in THF (0.09 mL), and aqueous 0.6 M LiOH (54 μL, 0.03 mmol) was added. The reaction mixture was stirred at rt for 17 h. After completion, the reaction was diluted with water, acidified with aqueous 1 M HCl, and extracted with EtOAc (×3). The organic phases were combined, washed with brine, dried over MgSO_4_, and filtered. The residue was concentrated *in vacuo* and purified by preparative HPLC (30 to 100% mobile phase B (MeCN–H_2_O–TFA 90:10:0.1) in mobile phase A (H_2_O–TFA 100:0.1) over 20 min, flow rate 20 mL/min). HPLC fractions were combined and concentrated *in vacuo*, and the remaining aqueous phase extracted with EtOAc (×2). The organic phases were combined, washed with brine, and concentrated *in vacuo* to give 2.7 mg (53%) of **7** as an orange solid (*t*_R_ = 9.77 min, purity >99% (254 and 450 nm) by HPLC, method A); ^1^H NMR (600 MHz, DMSO*-d*_*6*_) δ 9.58–9.53 (m, 1H), 8.62 (d, *J* = 8.2 Hz, 1H), 8.51 (d, *J* = 8.9 Hz, 1H), 7.84–7.79 (m, 2H), 7.17 (d, *J* = 3.6 Hz, 1H), 7.04–6.99 (m, 2H), 6.94 (d, *J* = 3.6 Hz, 1H), 6.44 (d, *J* = 9.0 Hz, 1H), 4.75 (q, *J* = 7.3 Hz, 1H), 4.04 (t, *J* = 6.4 Hz, 2H), 3.57–3.44 (m, 2H), 2.90–2.83 (m, 1H), 2.75–2.68 (m, 1H), 1.83–1.73 (m, 4H), 1.59–1.51 (m, 2H); HRMS (MALDI) calcd for C_26_H_25_N_5_O_10_ (M+H)^+^ 568.1674, found 568.1678.

### (5-(4-(3-((*tert*-Butoxycarbonyl)amino)propoxy)phenyl)furan-2-carbonyl)-l-aspartic acid (8a)

*tert*-Butyl (3-chloropropyl)carbamate: Triethylamine (2.3 mL, 16.50 mmol) was added dropwise to a stirred mixture of 3-chloropropylamine hydrochloride (726 mg, 5.58 mmol) dissolved in DCM (11 mL) at 0 °C, and stirring was continued for 30 min at 0 °C under an argon atmosphere. (Boc)_2_O (1344 mg, 6.16 mmol) was added to the mixture at 0 °C. After 5 min, the reaction was allowed to reach rt and stirred for 24 h. After completion, the reaction mixture was washed with aqueous 1 M HCl (×2) and brine. The aqueous phases were combined and re-extracted with DCM (×2). The organic phases were combined, dried over Na_2_SO_4_, filtered, and concentrated *in vacuo* to give 1038 mg (quant.) of the product as a yellow oil: *R*_f_ = 0.68 (EtOAc:*n*-heptane, 1:1); ^1^H NMR (400 MHz, CDCl_3_) δ 7.78 (d, *J* = 8.3 Hz, 2H), 7.34 (d, *J* = 8.1 Hz, 2H), 4.49 (br s, 1H), 4.03 (t, *J* = 6.3 Hz, 2H), 3.11–3.03 (m, 2H), 2.45 (s, 3H), 1.73–1.62 (m, 2H), 1.56–1.43 (m, 2H), 1.42 (s, 9H); ^13^C NMR (101 MHz, CDCl_3_) δ 156.1, 79.6, 42.5, 38.1, 32.7, 28.5; Spectra in accordance with reported data.^39^

**4** (29 mg, 0.08 mmol) was reacted with K_2_CO_3_ (27 mg, 0.20 mmol), KI (16 mg, 0.10 mmol), and *tert*-butyl (3-chloropropyl)carbamate (40 μL, 0.22 mmol) in MeCN (0.45 mL) as described for **5c**. Purification by flash column chromatography (SiO_2_, 0–1% MeOH in DCM) gave 23 mg (55%) of **5a** as a colorless oil: *R*_f_ = 0.34 (MeOH:DCM; 1:20); ^1^H NMR (400 MHz, CDCl_3_) δ 7.69–7.61 (m, 2H), 7.34 (d, *J* = 8.2 Hz, 1H), 7.19 (d, *J* = 3.6 Hz, 1H), 6.98–6.90 (m, 2H), 6.61 (d, *J* = 3.7 Hz, 1H), 5.10–5.01 (m, 1H), 4.76 (br s, 1H), 4.06 (t, *J* = 6.0 Hz, 2H), 3.81 (s, 3H), 3.72 (s, 3H), 3.39–3.30 (m, 2H), 3.20–3.10 (m, 1H), 3.03–2.93 (m, 1H), 2.00 (p, *J* = 6.3 Hz, 2H), 1.45 (s, 9H); ^13^C NMR (101 MHz, CDCl_3_) δ 171.7, 171.3, 159.5, 158.1, 156.20, 156.15, 145.8, 126.3, 122.7, 117.5, 115.0, 106.0, 77.4, 66.0, 53.1, 52.2, 48.4, 38.1, 36.4, 29.7, 28.6; ESI-MS *m*/*z* 449.4 (M-Boc+HCOO^–^+H^+^).

**5a** (23 mg, 0.05 mmol) was hydrolyzed using aqueous 0.6 M LiOH (0.25 mL, 0.15 mmol) as described for **7** to give 22 mg (quant.) of **8a** as a yellow oil (*t*_R_ = 4.59 min, purity 96.3% by HPLC, method B); ^1^H NMR (600 MHz, CD_3_OD) δ 7.80–7.72 (m, 2H), 7.20 (d, *J* = 3.6 Hz, 1H), 7.02–6.96 (m, 2H), 6.77 (d, *J* = 3.6 Hz, 1H), 4.99–4.94 (m, 1H), 4.06 (t, *J* = 6.2 Hz, 2H), 3.24 (t, *J* = 6.8 Hz, 2H), 3.05–2.93 (m, 2H), 1.95 (p, *J* = 6.5 Hz, 2H), 1.43 (s, 9H); ^13^C NMR (151 MHz, CD_3_OD) δ 174.3, 174.0, 161.1, 160.5, 158.6, 158.0, 146.8, 127.3, 123.8, 118.2, 115.9, 106.6, 80.0, 66.8, 50.1, 38.4, 36.9, 30.7, 28.8; HRMS (MALDI) calcd for C_23_H_28_N_2_O_9_ (M+Na)^+^ 499.1686, found 499.1689; [α]^20^_D_ +17.8° (*c* 0.24, MeOH).

### (5-(4-(4-((*tert*-Butoxycarbonyl)amino)butoxy)phenyl)furan-2-carbonyl)-l-aspartic acid (8b)

*tert*-Butyl (4-hydroxybutyl)carbamate: 4-Aminobutan-1-ol (0.41 mL, 4.45 mmol) was Boc-protected as described for *tert*-butyl (5-hydroxypentyl)carbamate to give the product as an amber oil that was used directly in the next step (*R*_f_ = 0.66 (10% MeOH in DCM)).

4-((*tert*-Butoxycarbonyl)amino)butyl 4-methylbenzenesulfonate: *tert*-Butyl (4-hydroxybutyl)carbamate was dissolved in DCM (11 mL) in a flask under an argon atmosphere. The mixture was cooled on an ice–water bath before addition of tosyl chloride (1284 mg, 6.73 mmol) followed by pyridine (0.9 mL, 11.13 mmol). The reaction mixture was stirred at rt for 14 h. After completion, the reaction mixture was washed with aqueous 1 M HCl (×2). The aqueous phases were combined and re-extracted with DCM (×2). The organic phases were combined, washed with brine, dried over Na_2_SO_4_, filtered, and concentrated *in vacuo*. The residue was purified by flash column chromatography (SiO_2_, 0–50% EtOAc in *n*-heptane) to give 1295 mg (85% over two steps) of the product as a white gel-like solid: *R*_f_ = 0.54 (EtOAc:*n*-heptane, 1:2); ^1^H NMR (400 MHz, CDCl_3_) δ 7.78 (d, *J* = 8.3 Hz, 2H), 7.34 (d, *J* = 8.1 Hz, 2H), 4.49 (s, 1H), 4.03 (t, *J* = 6.3 Hz, 2H), 3.07 (t, *J* = 7.0 Hz, 2H), 2.45 (s, 3H), 1.73–1.62 (m, 2H), 1.56–1.43 (m, 2H), 1.42 (s, 9H); ^13^C NMR (101 MHz, CDCl_3_) δ 156.1, 144.9, 133.3, 130.0, 128.0, 70.2, 38.2, 28.5, 26.4, 21.8; ESI-MS (method B) *m*/*z* 244.3 (M+H^+^-Boc). Spectra in accordance with reported data.^40^

**4** (20 mg, 0.06 mmol) was reacted with K_2_CO_3_ (17 mg, 0.12 mmol) and 4-((*tert*-butoxycarbonyl)amino)butyl 4-methylbenzenesulfonate (50 mg, 0.15 mmol) in MeCN (0.50 mL) as described for **5c**. Purification by flash column chromatography (SiO_2_, 0–1% MeOH in DCM) gave 11 mg (37%) of **5b** as a white foam: *R*_f_ = 0.39 (5% MeOH in DCM); ^1^H NMR (400 MHz, CDCl_3_) δ 7.68–7.60 (m, 2H), 7.34 (d, *J* = 8.1 Hz, 1H), 7.19 (d, *J* = 3.6 Hz, 1H), 6.97–6.89 (m, 2H), 6.60 (d, *J* = 3.6 Hz, 1H), 5.10–5.01 (m, 1H), 4.62 (br s, 1H), 4.02 (t, *J* = 6.2 Hz, 2H), 3.81 (s, 3H), 3.72 (s, 3H), 3.23–3.11 (m, 3H), 3.03–2.93 (m, 1H), 1.90–1.78 (m, 2H), 1.75–1.63 (m, 3H), 1.45 (s, 9H); ^13^C NMR (101 MHz, CDCl_3_) δ 171.8, 171.3, 159.6, 158.2, 156.3, 156.2, 145.8, 126.3, 122.6, 117.5, 115.0, 105.9, 79.3, 67.7, 53.1, 52.2, 48.4, 40.4, 36.4, 28.6, 27.0, 26.7; ESI-MS *m*/*z* 519.2 (M+H^+^).

**5b** (10 mg, 0.02 mmol) was hydrolyzed using aqueous 0.6 M LiOH (100 μL, 0.06 mmol) as described for **7** to give 10 mg (97%) of **8b** as a colorless oil (*t*_R_ = 4.88 min, purity 97.6% by HPLC, method B); ^1^H NMR (600 MHz, CD_3_OD) δ 7.83–7.76 (m, 2H), 7.23 (d, *J* = 3.62 Hz, 1H), 7.04–6.98 (m, 2H), 6.79 (d, *J* = 3.62 Hz, 1H), 5.00–4.95 (m, 1H), 4.07 (t, *J* = 6.32 Hz, 2H), 3.14 (t, *J* = 6.97 Hz, 2H), 3.07–2.95 (m, 2H), 1.88–1.80 (m, 2H), 1.72–1.65 (m, 2H), 1.46 (s, 9H); ^13^C NMR (151 MHz, CD_3_OD) δ 174.5, 174.2, 161.2, 160.5, 158.6, 158.1, 146.8, 127.3, 123.8, 118.2, 115.9, 106.6, 79.9, 68.8, 50.2, 41.1, 37.0, 28.8, 27.6; HRMS (MALDI) calcd for C_24_H_30_N_2_O_9_ (M+Na)^+^ 513.1843, found 513.1848; [α]^25^_D_ +4.1° (*c* 0.12, MeOH).

### (5-(4-((5-((*tert*-Butoxycarbonyl)amino)pentyl)oxy)phenyl)furan-2-carbonyl)-l-aspartic acid (8c)

**5c** (13 mg, 0.02 mmol) was hydrolyzed using aqueous 0.6 M LiOH (150 μL, 0.07 mmol) as described for **7** to give 10 mg (82%) of **8c** as a colorless oil (*t*_R_ = 5.27 min, purity 95.8% by HPLC, method B); ^1^H NMR (600 MHz, CD_3_OD) δ 7.79–7.74 (m, 2H), 7.20 (d, *J* = 3.6 Hz, 1H), 7.01–6.94 (m, 2H), 6.77 (d, *J* = 3.6 Hz, 1H), 4.98–4.93 (m, 1H), 4.03 (t, *J* = 6.4 Hz, 2H), 3.07 (t, *J* = 6.6 Hz, 2H), 3.05–2.93 (m, 2H), 1.85–1.77 (m, 2H), 1.60–1.47 (m, 4H), 1.43 (s, 9H); ^13^C NMR (151 MHz, CD_3_OD) δ 174.4, 174.1, 161.2, 160.5, 158.6, 158.1, 146.8, 127.3, 123.7, 118.2, 115.9, 106.6, 79.8, 69.0, 50.2, 41.2, 37.0, 30.7, 30.0, 28.8, 24.4; HRMS (MALDI) calcd for C_25_H_32_N_2_O_9_ (M+Na)^+^ 527.1999, found 527.2005; [α]^25^_D_ +27.2° (*c* 0.14, MeOH).

### (5-(4-((6-((*tert*-Butoxycarbonyl)amino)hexyl)oxy)phenyl)furan-2-carbonyl)-l-aspartic acid (8d)

*tert*-Butyl (6-hydroxyhexyl)carbamate: 6-Aminohexan-1-ol (395 mg, 3.37 mmol) was Boc-protected as described for *tert*-butyl (5-hydroxypentyl)carbamate and purified by flash column chromatography (SiO_2_, 0–20% EtOAc in *n*-heptane) to give 363 mg (50%) of the product as a clear oil: *R*_f_ = 0.31 (EtOAc:*n*-heptane; 1:1); ^1^H NMR (400 MHz, CDCl_3_) δ 3.63 (t, *J* = 6.5 Hz, 2H), 3.10 (t, *J* = 7.0 Hz, 2H), 1.62–1.45 (m, 4H), 1.43 (s, 9H), 1.41–1.24 (m, 4H); ^13^C NMR (101 MHz, CDCl_3_) δ 156.3, 62.9, 40.7, 32.7, 30.2, 28.6, 26.5, 25.4. Spectra in accordance with reported data.^41^

6-((*tert*-Butoxycarbonyl)amino)hexyl 4-methylbenzenesulfonate: *tert*-Butyl (6-hydroxyhexyl)carbamate (180 mg, 0.83 mmol) was tosylated as described for 5-((*tert*-butoxycarbonyl)amino)pentyl 4-methylbenzenesulfonate and purified by flash column chromatography (SiO_2_, 0–30% EtOAc in *n*-heptane) to give 179 mg (58%) of the product as a clear oil: *R*_f_ = 0.36 (EtOAc:*n*-heptane, 1:2); ^1^H NMR (600 MHz, CDCl_3_) δ 7.80–7.76 (m, 2H), 7.36–7.32 (m, 2H), 4.47 (br s, 1H), 4.01 (t, *J* = 6.5 Hz, 2H), 3.08–3.03 (m, 2H), 2.45 (s, 3H), 1.67–1.60 (m, 2H), 1.46–1.38 (m, 11H), 1.36–1.21 (m, 4H); ^13^C NMR (151 MHz, CDCl_3_) δ 156.1, 144.8, 133.4, 130.0, 128.0, 79.3, 70.6, 40.5, 30.0, 28.9, 28.6, 26.3, 25.2, 21.8; ESI-MS (method B) *m*/*z* 372.2 (M+H^+^). Spectra in accordance with reported data.^42^

**4** (49 mg, 0.14 mmol) was reacted with K_2_CO_3_ (40 mg, 0.29 mmol) and 6-((*tert*-butoxycarbonyl)amino)hexyl 4-methylbenzenesulfonate (76 mg, 0.20 mmol) in MeCN (1.0 mL) as described for **5c**. Purification by flash column chromatography (SiO_2_, 0–50% EtOAc in *n*-heptane) gave 60 mg (78%) of **5d** as a white foam: *R*_f_ = 0.24 (EtOAc:*n*-heptane, 1:1); ^1^H NMR (400 MHz, CDCl_3_) δ 7.65 (d, *J* = 8.5 Hz, 2H), 7.34 (d, *J* = 8.1 Hz, 1H), 7.20 (d, *J* = 3.6 Hz, 1H), 6.93 (d, *J* = 8.5 Hz, 2H), 6.60 (d, *J* = 3.6 Hz, 1H), 5.10–5.02 (m, 1H), 4.50 (br s, 1H), 3.99 (t, *J* = 6.4 Hz, 2H), 3.81 (s, 3H), 3.73 (s, 3H), 3.21–3.08 (m, 3H), 3.03–2.93 (m, 1H), 1.81 (p, *J* = 6.8 Hz, 2H), 1.52 (p, *J* = 7.4 Hz, 4H), 1.46 (s, 9H), 1.39 (t, *J* = 7.6 Hz, 2H); ^13^C NMR (101 MHz, CDCl_3_) δ 171.8, 171.3, 159.8, 158.2, 156.3, 145.8, 126.3, 122.5, 117.5, 115.0, 105.9, 68.1, 53.1, 52.2, 48.4, 36.4, 30.2, 29.3, 28.6, 26.7, 25.9; ESI-MS *m*/*z* 547.3 (M+H^+^).

**5d** (10 mg, 0.02 mmol) was hydrolyzed using aqueous 0.6 M LiOH (0.1 mL, 0.06 mmol) as described for **7** to give 6 mg (64%) of **8d** as a white solid (*t*_R_ = 5.77 min, purity 95.0% by HPLC, method B); ^1^H NMR (600 MHz, DMSO-*d*_*6*_) δ 12.66 (br s, 2H), 8.64 (d, *J* = 8.3 Hz, 1H), 7.85–7.79 (m, 2H), 7.17 (d, *J* = 3.6 Hz, 1H), 7.05–6.98 (m, 2H), 6.94 (d, *J* = 3.6 Hz, 1H), 6.78–6.73 (m, 1H), 4.79–4.73 (m, 1H), 4.01 (t, *J* = 6.5 Hz, 2H), 2.94–2.83 (m, 3H), 2.76–2.69 (m, 1H), 1.71 (p, *J* = 6.7 Hz, 2H), 1.44–1.34 (m, 13H), 1.34–1.27 (m, 2H); ^13^C NMR (151 MHz, DMSO-*d*_*6*_) δ 172.4, 171.9, 159.1, 157.4, 155.6, 154.9, 145.9, 126.0, 122.0, 116.3, 114.8, 105.9, 77.3, 67.5, 48.5, 39.9, 35.9, 29.4, 28.6, 28.3, 26.0, 25.2; HRMS (MALDI) calcd for C_26_H_34_N_2_O_9_ (M+Na)^+^ 541.2156, found 541.2158; [α]^20^_D_ +7.0° (*c* 0.12, MeOH).

### (5-(4-(2-(2-((*tert*-Butoxycarbonyl)amino)ethoxy)ethoxy)phenyl)furan-2-carbonyl)-l-aspartic acid (8e)

*tert*-Butyl (2-(2-hydroxyethoxy)ethyl)carbamate: 2-(2-aminoethoxy)ethan-1-ol (0.53 mL, 5.28 mmol) was Boc-protected as described for *tert*-butyl (5-hydroxypentyl)carbamate to give the product as a colorless oil (813 mg, 75%) that was used directly in the next step (*R*_f_ = 0.40 (10% MeOH in DCM)).

2-(2-((*tert*-Butoxycarbonyl)amino)ethoxy)ethyl 4-methylbenzenesulfonate: *tert*-Butyl (2-(2-hydroxyethoxy)ethyl)carbamate (813 mg, 3.96 mmol) was tosylated as described for 5-((*tert*-butoxycarbonyl)amino)pentyl 4-methylbenzenesulfonate and purified by flash column chromatography (SiO_2_, 0–50% EtOAc in *n*-heptane) to give 1101 mg (77%) of the product as a colorless oil: *R*_f_ = 0.51 (EtOAc:*n*-heptane, 1:1); ^1^H NMR (400 MHz, CDCl_3_) δ 7.84–7.77 (m, 1H), 7.35 (d, *J* = 8.1 Hz, 2H), 4.79 (br s, 1H), 4.19–4.14 (m, 2H), 3.66–3.59 (m, 2H), 3.45 (t, *J* = 5.1 Hz, 2H), 3.24 (q, *J* = 5.4 Hz, 2H), 2.45 (s, 3H), 1.45 (s, 9H); ^13^C NMR (CDCl_3_, 101 MHz): 156.8, 145.0, 133.2, 130.0, 128.1, 79.8, 70.5, 69.2, 68.5, 40.3, 28.6, 21.8; ESI-MS (method A) *m*/*z* 260.3 (M+H^+^-Boc). Spectra in accordance with reported data.^42^

**4** (30 mg, 0.09 mmol) was reacted with K_2_CO_3_ (24 mg, 0.174 mmol) and 2-(2-((*tert*-butoxycarbonyl)amino)ethoxy)ethyl 4-methylbenzenesulfonate (47 mg, 0.130 mmol) in MeCN (0.5 mL) as described for **5c**. Purification by flash column chromatography (SiO_2_, 0–75% EtOAc in *n*-heptane) gave 15 mg (33%) of **5e** as a colorless oil: *R*_f_ = 0.15 (EtOAc:*n*-heptane, 1:1); ^1^H NMR (400 MHz, CDCl_3_) δ 7.70–7.62 (m, 2H), 7.34 (d, *J* = 8.1 Hz, 1H), 7.20 (d, *J* = 3.6 Hz, 1H), 7.04–6.94 (m, 2H), 6.61 (d, *J* = 3.6 Hz, 1H), 5.10–5.01 (m, 1H), 4.18–4.14 (m, 2H), 3.86–3.83 (m, 2H), 3.81 (s, 3H), 3.72 (s, 3H), 3.62 (t, *J* = 5.2 Hz, 2H), 3.35 (t, *J* = 5.2 Hz, 2H), 3.20–3.10 (m, 1H), 3.03–2.93 (m, 1H), 1.44 (s, 9H); ^13^C NMR (101 MHz, CDCl_3_) δ 171.8, 171.3, 159.4, 158.1, 156.2, 156.1, 145.8, 126.3, 122.9, 117.5, 115.1, 106.1, 79.9, 70.6, 69.5, 67.6, 53.1, 52.2, 48.4, 36.4, 28.6; ESI-MS *m*/*z* 535.2 (M+H^+^); [α]^20^_D_ +0.8° (*c* 0.13, MeOH).

**5e** (8 mg, 0.01 mmol) was hydrolyzed using aqueous 0.6 M LiOH (75 μL, 0.05 mmol) as described for **7** to give 8 mg (quant.) of **8e** as a pale yellow oil (*t*_R_ = 9.15 min, purity 95.2% by HPLC, method A); ^1^H NMR (600 MHz, CD_3_OD) δ 7.81–7.74 (m, 2H), 7.21 (d, *J* = 3.6 Hz, 1H), 7.04–7.00 (m, 2H), 6.78 (d, *J* = 3.6 Hz, 1H), 4.99–4.94 (m, 1H), 4.20–4.15 (m, 2H), 3.85–3.81 (m, 2H), 3.58 (t, *J* = 5.7 Hz, 2H), 3.25 (t, *J* = 5.7 Hz, 2H), 3.05–2.92 (m, 2H), 1.42 (s, 9H); ^13^C NMR (151 MHz, CD_3_OD) δ 174.4, 174.0, 160.9, 160.5, 158.5, 157.9, 146.9, 127.3, 124.0, 118.2, 116.1, 106.7, 80.1, 71.2, 70.5, 68.8, 50.1, 41.3, 36.9, 28.7; HRMS (MALDI) calcd for C_24_H_30_N_2_O_10_ (M+Na)^+^ 529.1793, found 529.1798; [α]^20^_D_ +0.9° (*c* 0.23, MeOH).

### (*S*)-3-(4-(5-((1,2-Dicarboxyethyl)carbamoyl)furan-2-yl)phenoxy)propan-1-aminium 2,2,2-trifluoroacetate (9a)

**8a** (12 mg, 0.03 mmol) was dissolved in 1,4-dioxane (0.21 mL), and 1 M HCl in 1,4-dioxane (53 μL, 0.21 mmol) was added. The reaction mixture was stirred at rt for 20 h. After completion, the reaction mixture was concentrated *in vacuo*, and the residue was washed with diethyl ether and purified by preparative HPLC (0 to 100% mobile phase B (MeCN–H_2_O–TFA 90:10:0.1) in mobile phase A (H_2_O–TFA 100:0.1) over 20 min, flow rate 20 mL/min). HPLC fractions were combined and concentrated *in vacuo* to give 2.0 mg (15%) of **9a** as an off-white solid (*t*_R_ = 6.46 min, purity >99% by HPLC, method A); ^1^H NMR (600 MHz, CD_3_OD) δ 7.83–7.78 (m, 2H), 7.22 (d, *J* = 3.6 Hz, 1H), 7.06–7.01 (m, 2H), 6.80 (d, *J* = 3.6 Hz, 1H), 4.99–4.94 (m, 1H), 4.18 (t, *J* = 5.7 Hz, 2H), 3.20–3.15 (m, 2H), 3.04–2.94 (m, 2H), 2.21–2.13 (m, 2H); ^13^C NMR (151 MHz, CD_3_OD) δ 174.3, 173.9, 160.5, 157.8, 146.9, 127.3, 124.4, 118.1, 116.0, 106.8, 66.3, 50.1, 38.6, 36.8, 28.4; HRMS (MALDI) calcd for C_18_H_20_N_2_O_7_ (M+H)^+^ 377.1343, found 377.1344.

### (5-(4-(4-Aminobutoxy)phenyl)furan-2-carbonyl)-l-aspartic acid hydrochloride (9b)

**8b** (6 mg, 0.01 mmol) was Boc-deprotected using 4 M HCl in 1,4-dioxane (108 μL, 0.11 mmol) as described for **9a** without preparative HPLC purification to give 4 mg (73%) of **9b** as a yellow solid (*t*_R_ = 6.33 min, purity 96.2% by HPLC, method A); ^1^H NMR (400 MHz, CD_3_OD) δ 7.83–7.75 (m, 2H), 7.21 (d, *J* = 3.6 Hz, 1H), 7.04–6.96 (m, 2H), 6.78 (d, *J* = 3.7 Hz, 1H), 5.01–4.93 (m, 1H), 4.10 (t, *J* = 5.5 Hz, 2H), 3.66 (s, 2H), 3.07–2.89 (m, 4H), 1.97–1.81 (m, 4H); ^13^C NMR (151 MHz, CD_3_OD) δ 174.4, 174.1, 160.9, 160.5, 157.9, 146.9, 127.3, 124.0, 118.1, 115.9, 106.7, 68.4, 50.2, 40.6, 36.9, 27.2, 25.7; HRMS (MALDI) calcd for C_19_H_22_N_2_O_7_ (M+H)^+^ 391.1499, found 391.1498.

### (*S*)-5-(4-(5-((1,2-Dicarboxyethyl)carbamoyl)furan-2-yl)phenoxy)pentan-1-aminium 2,2,2-trifluoroacetate (9c)

**8c** (5 mg, 0.01 mmol) was Boc-deprotected using 4 M HCl in 1,4-dioxane (23 μL, 0.09 mmol) as described for **9a** to give 2.0 mg (39%) of **9c** as an off-white solid (*t*_R_ = 6.92 min, purity >99% by HPLC, method A); ^1^H NMR (600 MHz, CD_3_OD) δ 7.81–7.75 (m, 2H), 7.21 (d, *J* = 3.6 Hz, 1H), 7.02–6.96 (m, 2H), 6.78 (d, *J* = 3.6 Hz, 1H), 4.98–4.93 (m, 1H), 4.07 (t, *J* = 6.1 Hz, 2H), 3.04–2.93 (m, 4H), 1.90–1.83 (m, 2H), 1.79–1.71 (m, 2H), 1.65–1.57 (m, 2H); ^13^C NMR (151 MHz, CD_3_OD) δ 174.4, 174.1, 161.1, 160.5, 158.0, 146.8, 127.3, 123.9, 118.1, 115.9, 106.6, 68.6, 50.2, 40.7, 37.0, 29.8, 28.4, 24.2; HRMS (MALDI) calcd for C_20_H_24_N_2_O_7_ (M+H)^+^ 405.1656, found 405.1656.

### (*S*)-6-(4-(5-((1,2-Dicarboxyethyl)carbamoyl)furan-2-yl)phenoxy)hexan-1-aminium 2,2,2-trifluoroacetate (9d)

**8d** (19 mg, 0.04 mmol) was Boc-deprotected using 4 M HCl in 1,4-dioxane (75 μL, 0.30 mmol) as described for **9a** to give 7 mg (35%) of **9d** as a white solid of the TFA salt (*t*_R_ = 7.20 min, purity 96.6% by HPLC, method A): ^1^H NMR (600 MHz, CD_3_OD) δ 7.79–7.76 (m, 2H), 7.21 (d, *J* = 3.6 Hz, 1H), 7.01–6.95 (m, 2H), 6.77 (d, *J* = 3.6 Hz, 1H), 5.00–4.94 (m, 1H), 4.04 (t, *J* = 6.3 Hz, 2H), 3.05–2.91 (m, 4H), 1.87–1.79 (m, 2H), 1.70 (p, *J* = 7.8 Hz, 2H), 1.61–1.53 (m, 2H), 1.52–1.46 (m, 2H); ^13^C NMR (151 MHz, CD_3_OD) δ 174.3, 173.9, 161.2, 160.5, 158.1, 146.8, 127.3, 123.8, 118.2, 115.9, 106.6, 68.9, 50.1, 40.7, 36.8, 30.1, 28.5, 27.2, 26.7; HRMS (MALDI) calcd for C_21_H_26_N_2_O_7_ (M+H)^+^ 419.1812, found 419.1814.

### (6-(4-(3-((*tert*-Butoxycarbonyl)amino)propoxy)phenyl)picolinoyl)-l-aspartic acid (15a)

A flame-dried microwave vial under an argon atmosphere was charged with **10** (207 mg, 1.31 mmol), DCM (2 mL), *N,N*-diisopropylethylamine (0.97 mL, 5.57 mmol), and BTFFH (480 mg, 1.52 mmol). The reaction mixture was stirred at rt for 30 min before **11** (200 mg, 1.01 mmol) was added. After addition, the vial was sealed and heated to 80 °C for 12 h. The reaction was cooled to rt, diluted with water, and extracted with EtOAc (×3). The organic phases were combined, washed with brine, dried over Na_2_SO_4_, filtered, and concentrated *in vacuo*. The residue was purified by flash column chromatography (SiO_2_, 0–50% EtOAc in *n-*heptane) to give 267 mg (88%) of **12** as a yellow oil: *R*_f_ = 0.72 (EtOAc:*n*-heptane, 1:1); ^1^H NMR (400 MHz, CDCl_3_) δ 8.61–8.54 (m, 1H), 8.08 (d, *J* = 7.6 Hz, 1H), 7.80 (t, *J* = 7.8 Hz, 1H), 7.46 (d, *J* = 8.0 Hz, 1H), 5.09–5.00 (m, 1H), 3.78 (s, 3H), 3.70 (s, 3H), 3.15–3.05 (m, 1H), 3.00–2.90 (m, 1H); ^13^C NMR (101 MHz, CDCl_3_) δ 171.1, 170.9, 162.9, 150.4, 149.8, 140.0, 127.5, 121.2, 53.0, 52.2, 48.9, 36.3; ESI-MS *m*/*z* 301.2 (M+H^+^).

To a 25 mL round-bottom flask charged with **12** (535 mg, 1.78 mmol) were added (4-hydroxyphenyl)boronic acid (270 mg, 1.96 mmol) and XPhos-Pd-G4 (32 mg, 2 mol%) under an argon atmosphere. The flask was evacuated and backfilled with argon (×3). Afterward, THF (9 mL) and degassed aqueous 0.5 M K_3_PO_4_ (7.1 mL, 3.55 mmol) were added. The mixture was stirred for 21 h at rt. After completion, the reaction mixture was diluted with water and extracted with EtOAc (×3). The organic phases were combined, washed with brine, dried over Na_2_SO_4_, filtered, and concentrated *in vacuo*. The residue was purified by flash column chromatography (SiO_2_, 0–50% EtOAc in *n*-heptane) to give 394 mg (62%) of **13** as a dark brown solid: *R*_f_ = 0.17 (EtOAc:*n*-heptane, 1:1); ^1^H NMR (400 MHz, CDCl_3_) δ 9.19 (d, *J* = 8.5 Hz, 1H), 8.06–8.02 (m, 1H), 7.95–7.90 (m, 2H), 7.89–7.81 (m, 1H), 7.82–7.76 (m, 1H), 7.08 (br s, 1H), 7.01–6.93 (m, 2H), 5.16–5.07 (m, 1H), 3.81 (s, 3H), 3.72 (s, 3H), 3.23–3.13 (m, 1H), 3.07–2.95 (m, 1H); ^13^C NMR (101 MHz, CDCl_3_) δ 171.5, 171.4, 165.0, 158.0, 156.0, 148.5, 138.3, 130.2, 128.5, 122.5, 120.0, 116.0, 53.1, 52.3, 48.9, 36.4; ESI-MS *m*/*z* 359.4 (M+H^+^); [α]^20^_D_ +6.0° (*c* 0.29, MeOH).

*tert*-Butyl (3-chloropropyl)carbamate (17 mg, 0.09 mmol) was dissolved in MeCN (0.2 mL) in a dry flask under an argon atmosphere. Then, **13** (20 mg, 0.06 mmol), K_2_CO_3_ (17 mg, 0.12 mmol), KI (3 mg, 0.02 mmol), and MeCN (0.25 mL) were added to the flask. The reaction mixture was refluxed for 2 days. After completion, water was added to the reaction mixture. The aqueous phase was extracted with EtOAc (×3). The organic phases were combined, washed with brine, dried over MgSO_4_, filtered, and concentrated *in vacuo*. The residue was purified by flash column chromatography (SiO_2_, EtOAc:*n*-heptane, 1:1) to give 7 mg (23%) of **14a** as a brown oil: *R*_f_ = 0.43 (EtOAc:*n*-heptane, 2:1); ^1^H NMR (400 MHz, CDCl_3_) δ 9.10 (d, *J* = 8.5 Hz, 1H), 8.09–7.98 (m, 3H), 7.91–7.81 (m, 2H), 7.05–7.00 (m, 2H), 5.14–5.05 (m, 1H), 4.76 (br s, 1H), 4.10 (t, *J* = 6.0 Hz, 2H), 3.81 (s, 3H), 3.73 (s, 3H), 3.40–3.31 (m, 2H), 3.22–3.12 (m, 1H), 3.05–2.95 (m, 1H), 2.08–1.97 (m, 2H), 1.45 (s, 9H); ESI-MS *m*/*z* 516.52 (M+H^+^).

**14a** (7 mg, 0.01 mmol) was hydrolyzed using aqueous 0.6 M LiOH (67 μL, 0.04 mmol) as described for **7** to give 4 mg (82%) of **15a** as a colorless oil (*t*_R_ = 4.92 min, purity 96.0% by HPLC, method B); ^1^H NMR (600 MHz, CD_3_OD) δ 8.15 (d, *J* = 8.8 Hz, 2H), 8.07–8.03 (m, 1H), 8.01–7.98 (m, 2H), 7.09–7.05 (m, 2H), 5.01 (t, *J* = 4.9 Hz, 1H), 4.12 (t, *J* = 6.1 Hz, 2H), 3.29 (t, *J* = 6.8 Hz, 2H), 3.15 (m, 1H), 3.01 (m, 1H), 2.00 (p, *J* = 6.5 Hz, 2H), 1.47 (s, 9H); ^13^C NMR (151 Hz, CD_3_OD) δ 174.5, 173.9, 166.4, 161.9, 158.6, 157.3, 150.0, 139.6, 131.8, 129.4, 123.5, 120.7, 115.8, 80.0, 66.7, 50.0, 38.5, 37.0, 30.8, 28.8; HRMS (MALDI) calcd for C_24_H_29_N_3_O_8_ (M+H)^+^ 488.2027, found 488.2029.

### (6-(4-(4-((*tert*-Butoxycarbonyl)amino)butoxy)phenyl)picolinoyl)-l-aspartic acid (15b)

**13** (20 mg, 0.06 mmol) was reacted with K_2_CO_3_ (17 mg, 0.12 mmol) and 4-((*tert*-butoxycarbonyl)amino)butyl 4-methylbenzenesulfonate (47 mg, 0.14 mmol) in MeCN (0.50 mL) as described for **5c** but only with heating to 65 °C. Purification by flash column chromatography (SiO_2_, 0–5% MeOH in DCM) gave 15 mg (50%) of **14b** as a yellow oil: *R*_f_ = 0.31 (5% MeOH in DCM); ^1^H NMR (400 MHz, CDCl_3_) δ 9.09 (d, *J* = 8.5 Hz, 1H), 8.09–8.04 (m, 1H), 8.03–7.98 (m, 2H), 7.91–7.79 (m, 2H), 7.05–6.97 (m, 2H), 5.14–5.05 (m, 1H), 4.63 (br s, 1H), 4.05 (t, *J* = 6.2 Hz, 2H), 3.81 (s, 3H), 3.73 (s, 3H), 3.26–3.09 (m, 3H), 3.05–2.95 (m, 1H), 1.91–1.80 (m, 2H), 1.75–1.66 (m, 2H), 1.45 (s, 9H); ^13^C NMR (101 MHz, CDCl_3_) δ 171.4, 171.3, 164.5, 160.4, 156.2, 155.8, 148.9, 138.2, 130.7, 128.4, 122.4, 120.1, 114.9, 79.3, 67.8, 53.0, 52.2, 48.8, 40.4, 36.5, 28.6, 27.0, 26.7; ESI-MS *m*/*z* 530.3 (M+H^+^).

**14b** (15 mg, 0.03 mmol) was hydrolyzed using aqueous 0.6 M LiOH (160 μL, 0.07 mmol) as described for **7** to give 11 mg (79%) of **15b** as a colorless oil (*t*_R_ = 5.22 min, purity 96.8% by HPLC, method B); ^1^H NMR (600 MHz, CD_3_OD) δ 8.14–8.08 (m, 2H), 8.03–7.97 (m, 1H), 7.99–7.94 (m, 2H), 7.06–7.00 (m, 2H), 4.97 (t, *J* = 5.0 Hz, 1H), 4.06 (t, *J* = 6.3 Hz, 2H), 3.15–3.07 (m, 3H), 3.01–2.95 (m, 1H), 1.88–1.79 (m, 2H), 1.71–1.63 (m, 2H), 1.44 (s, 9H); ^13^C NMR (151 MHz, CD_3_OD) δ 174.5, 174.1, 166.4, 161.9, 158.6, 157.2, 150.0, 139.6, 131.7, 129.3, 123.5, 120.6, 115.8, 79.9, 68.8, 50.1, 41.1, 37.1, 28.8, 27.7; HRMS (MALDI) calcd for C_25_H_31_N_3_O_8_ (M+H)^+^ 502.2183, found 502.2180; [α]^25^_D_ +5.1° (*c* 0.16, MeOH).

### (6-(4-((5-((*tert*-Butoxycarbonyl)amino)pentyl)oxy)phenyl)picolinoyl)-l-aspartic acid (15c)

**13** (20 mg, 0.06 mmol) was reacted with K_2_CO_3_ (17 mg, 0.12 mmol) and 5-((*tert*-butoxycarbonyl)amino)pentyl 4-methylbenzenesulfonate (29 mg, 0.08 mmol) in MeCN (0.45 mL) as described for **5c**. Purification by flash column chromatography (SiO_2_, EtOAc:*n*-heptane, 1:1) gave 24 mg (79%) of **14c** as a colorless thick oil: *R*_f_ = 0.47 (EtOAc:*n*-heptane, 2:1); ^1^H NMR (400 MHz, CDCl_3_) δ 9.20 (d, *J* = 8.6 Hz, 1H), 8.07–8.04 (m, 1H), 8.03–7.98 (m, 2H), 7.93–7.81 (m, 2H), 7.05–6.97 (m, 2H), 5.15–5.06 (m, 1H), 4.04 (t, *J* = 6.3 Hz, 2H), 3.81 (s, 3H), 3.73 (s, 3H), 3.23–3.13 (m, 3H), 3.05–2.95 (m, 1H), 1.85 (p, *J* = 6.6 Hz, 2H), 1.64–1.40 (m, 13H); ^13^C NMR (101 MHz, CDCl_3_) δ 171.5, 171.2, 164.9, 160.6, 155.9, 148.6, 138.3, 130.6, 128.4, 122.6, 120.2, 115.0, 68.0, 53.1, 52.3, 48.9, 36.4, 29.0, 28.5, 23.5; ESI-MS *m*/*z* 544.53 (M+H^+^).

**14c** (10 mg, 0.02 mmol) was hydrolyzed using aqueous 0.6 M LiOH (0.10 mL, 0.06 mmol) as described for **7** to give 7 mg (73%) of **15c** as a white solid (*t*_R_ = 5.63 min, purity 95.9% by HPLC, method B); ^1^H NMR (600 MHz, DMSO-*d*_*6*_) δ 9.18 (d, *J* = 8.7 Hz, 1H), 8.23–8.16 (m, 2H), 8.16–8.11 (m, 1H), 8.03 (t, *J* = 7.8 Hz, 1H), 7.95–7.91 (m, 1H), 7.09–7.03 (m, 2H), 6.81–6.76 (m, 1H), 4.89–4.83 (m, 1H), 4.04 (t, *J* = 6.5 Hz, 2H), 2.97–2.86 (m, 4H), 1.74 (p, *J* = 6.7 Hz, 2H), 1.48–1.35 (m, 13H); ^13^C NMR (151 MHz, DMSO-*d*_*6*_) δ 172.4, 172.2, 163.5, 160.0, 155.6, 154.7, 148.9, 138.8, 129.7, 128.2, 122.2, 119.7, 114.7, 77.3, 67.6, 48.5, 35.9, 29.2, 28.3, 28.3, 22.8; HRMS (MALDI) calcd for C_26_H_33_N_3_O_8_ (M+H)^+^ 516.2340, found 516.2340; [α]^25^_D_ +7.8° (*c* 0.10, MeOH).

### (6-(4-((6-((*tert*-Butoxycarbonyl)amino)hexyl)oxy)phenyl)picolinoyl)-l-aspartic acid (15d)

**13** (19 mg, 0.05 mmol) was reacted with K_2_CO_3_ (16 mg, 0.12 mmol) and 6-((*tert*-butoxycarbonyl)amino)hexyl 4-methylbenzenesulfonate (20 μL, 0.08 mmol) in MeCN (0.40 mL) as described for **5c**. Purification by flash column chromatography (SiO_2_, EtOAc:heptane, 2:1) gave 18 mg (60%) of **14d** as a colorless oil: *R*_f_ = 0.56 (EtOAc:*n*-heptane, 2:1); ^1^H NMR (400 MHz, CDCl_3_) δ 9.10 (d, *J* = 8.5 Hz, 1H), 8.09–7.97 (m, 3H), 7.91–7.80 (m, 2H), 7.05–6.97 (m, 2H), 5.14–5.05 (m, 1H), 4.52 (br s, 1H), 4.03 (t, *J* = 6.5 Hz, 2H), 3.81 (s, 3H), 3.73 (s, 3H), 3.21–3.09 (m, 3H), 3.05–2.95 (m, 1H), 1.82 (p, *J* = 6.7 Hz, 2H), 1.58–1.33 (m, 15H); ^13^C NMR (101 MHz, CDCl_3_) δ 171.4, 171.3, 164.5, 160.6, 155.8, 148.9, 138.2, 130.6, 128.4, 122.4, 120.1, 115.0, 68.1, 53.0, 52.2, 48.8, 36.5, 30.2, 29.3, 28.6, 26.7, 25.9; ESI-MS *m*/*z* 558.60 (M+H^+^).

**14d** (18.4 mg, 0.03 mmol) was hydrolyzed using aqueous 0.6 M LiOH (170 μL, 0.10 mmol) as described for **7** to give 17 mg (97%) of **15d** as a white solid (*t*_R_ = 6.21 min, purity >99% by HPLC, method B); ^1^H NMR (600 MHz, CD_3_OD) δ 8.15–8.09 (m, 2H), 8.05–8.00 (m, 1H), 7.98–7.95 (m, 2H), 7.06–7.00 (m, 2H), 4.98 (t, *J* = 4.9 Hz, 1H), 4.05 (t, *J* = 6.4 Hz, 2H), 3.16–3.09 (m, 1H), 3.05 (t, *J* = 7.0 Hz, 2H), 3.02–2.95 (m, 1H), 1.86–1.78 (m, 2H), 1.56–1.48 (m, 4H), 1.45–1.36 (m, 11H); ^13^C NMR (151 MHz, CD_3_OD) δ 174.4, 173.9, 166.4, 162.0, 158.6, 157.3, 149.9, 139.6, 131.6, 129.3, 123.5, 120.6, 115.8, 79.8, 69.0, 50.0, 41.3, 36.9, 30.9, 30.3, 28.8, 27.6, 26.9; HRMS (MALDI) calcd for C_27_H_35_N_3_O_8_ (M+H)^+^ 530.2496, found 530.2491; [α]^25^_D_ +2.6° (*c* 0.1, MeOH)

### (6-(4-(2-(2-((*tert*-Butoxycarbonyl)amino)ethoxy)ethoxy)phenyl)picolinoyl)-l-aspartic acid (15e)

**13** (20 mg, 0.06 mmol) was reacted with K_2_CO_3_ (17 mg, 0.12 mmol) and 2-(2-((*tert*-butoxycarbonyl)amino)ethoxy)ethyl 4-methylbenzenesulfonate (25 μL, 0.08 mmol) in MeCN (0.25 mL) as described for **5c**. Purification by flash column chromatography (SiO_2_, 0–75% EtOAc in *n*-heptane) gave 19 mg (64%) of **14e** as a colorless oil: *R*_f_ = 0.34 (EtOAc:*n*-heptane, 2:1); ^1^H NMR (400 MHz, CDCl_3_) δ 9.08 (d, *J* = 8.5 Hz, 1H), 8.10–7.96 (m, 3H), 7.92–7.78 (m, 2H), 7.09–7.01 (m, 2H), 5.14–5.05 (m, 1H), 4.98 (br s, 1H), 4.23–4.16 (m, 2H), 3.89–3.83 (m, 2H), 3.80 (s, 3H), 3.72 (s, 3H), 3.67–3.60 (m, 2H), 3.40–3.31 (m, 2H), 3.21–3.11 (m, 1H), 3.05–2.95 (m, 1H), 1.44 (s, 9H); ^13^C NMR (101 MHz, CDCl_3_) δ 171.4, 171.3, 164.5, 160.2, 156.1, 155.7, 149.0, 138.2, 131.1, 128.4, 122.4, 120.2, 115.1, 79.4, 70.6, 69.5, 67.6, 53.0, 52.2, 48.8, 40.5, 36.5, 28.5; ESI-MS *m*/*z* 546.3 (M+H^+^).

**14e** (19 mg, 0.04 mmol) was hydrolyzed using aqueous 0.6 M LiOH (180 μL, 0.11 mmol) as described for **7** to give 14 mg (77%) of **15e** as a colorless oil (*t*_R_ = 4.63 min, purity >99% by HPLC, method B); ^1^H NMR (600 MHz, CD_3_OD) δ 8.13–8.09 (m, 2H), 8.01–7.98 (m, 1H), 7.98–7.94 (m, 2H), 7.08–7.03 (m, 2H), 4.98 (t, *J* = 4.9 Hz, 1H), 4.21–4.15 (m, 2H), 3.86–3.82 (m, 2H), 3.59 (t, *J* = 5.7 Hz, 2H), 3.26 (t, *J* = 5.7 Hz, 2H), 3.16–3.08 (m, 1H), 3.02–2.95 (m, 1H), 1.42 (s, 9H); ^13^C NMR (151 Hz, CD_3_OD) δ 174.4, 173.9, 166.3, 161.7, 158.5, 157.1, 149.9, 139.6, 132.0, 129.4, 123.5, 120.7, 115.9, 80.1, 71.3, 70.5, 68.7, 50.0, 41.3, 36.9, 28.7; HRMS (MALDI) calcd for C_25_H_31_N_3_O_9_ (M+H)^+^ 518.2133, found 518.2131; [α]^20^_D_ +9.3° (*c* 0.18, MeOH).

### (*S*)*-N*-(2-((*tert*-Butoxycarbonyl)amino)ethyl)-6-(4-(5-((1,2-dicarboxyethyl)carbamoyl)furan-2-yl)phenoxy)hexan-1-aminium 2,2,2-trifluoroacetate (17)

**4** (100 mg, 0.29 mmol) was reacted with K_2_CO_3_ (159 mg, 1.15 mmol) in MeCN (1.4 mL) for 30 min at rt before addition of 1,6-dibromohexane (0.11 mL, 0.72 mmol) and then the reaction mixture was heated to 80 °C and stirred for 3.5 h under an argon atmosphere as described for **5c**. Purification by flash column chromatography (SiO_2_, 0–50% EtOAc in *n*-heptane) gave 104 mg (71%) of **16** as a clear oil: *R*_f_ = 0.60 (EtOAc:*n*-heptane, 2:1); ^1^H NMR (600 MHz, CDCl_3_) δ 7.68–7.62 (m, 2H), 7.34 (d, *J* = 8.1 Hz, 1H), 7.20 (d, *J* = 3.6 Hz, 1H), 6.97–6.91 (m, 2H), 6.61 (d, *J* = 3.6 Hz, 1H), 5.09–5.03 (m, 1H), 4.01 (t, *J* = 6.4 Hz, 2H), 3.81 (s, 3H), 3.73 (s, 3H), 3.44 (t, *J* = 6.8 Hz, 2H), 3.18–3.12 (m, 1H), 3.02–2.95 (m, 1H), 1.95–1.87 (m, 2H), 1.87–1.79 (m, 2H), 1.56–1.49 (m, 4H); ^13^C NMR (151 MHz, CDCl_3_) δ 171.8, 171.3, 159.8, 158.2, 156.3, 145.8, 126.3, 122.5, 117.5, 115.0, 105.9, 68.0, 53.1, 52.3, 48.4, 36.4, 33.9, 32.8, 29.2, 28.1, 25.4; ESI-MS *m*/*z* 510.1 (M+H^+^); [α]^20^_D_ −5.3° (*c* 0.17, MeOH).

Dimethyl (5-(4-((6-((2-((*tert*-butoxycarbonyl)amino)ethyl)amino)hexyl)oxy)phenyl)furan-2-carbonyl)-l-aspartate: **16** (37 mg, 0.07 mmol) was reacted with K_2_CO_3_ (20 mg, 0.14 mmol), KI (12 mg, 0.07 mmol), and *tert*-butyl (2-aminoethyl)carbamate (23 μL, 0.15 mmol) in MeCN (0.30 mL) as described for **5c**. Purification by flash column chromatography (SiO_2_, 0–10% MeOH in DCM) gave 18 mg (41%) of the product as a clear oil: *R*_f_ = 0.21 (DCM:MeOH, 10:1); ^1^H NMR (400 MHz, CDCl_3_) δ 7.67–7.59 (m, 2H), 7.35 (d, *J* = 8.1 Hz, 1H), 7.19 (d, *J* = 3.5 Hz, 1H), 6.95–6.88 (m, 2H), 6.62–6.56 (m, 1H), 5.10–5.01 (m, 1H), 4.01–3.94 (m, 2H), 3.80 (s, 3H), 3.72 (s, 3H), 3.37–3.31 (m, 2H), 3.19–3.09 (m, 1H), 3.03–2.85 (m, 3H), 2.84–2.75 (m, 2H), 1.85–1.73 (m, 2H), 1.69–1.57 (m, 2H), 1.53–1.35 (m, 13H); ^13^C NMR (101 MHz, CDCl_3_) δ 171.7, 171.3, 159.8, 158.2, 156.3, 145.7, 126.3, 122.4, 117.5, 115.0, 105.9, 68.0, 53.1, 52.2, 48.4, 36.4, 29.2, 28.5, 26.9, 25.9; ESI-MS *m*/*z* 590.3 (M+H^+^).

Dimethyl (5-(4-((6-((2-((*tert*-butoxycarbonyl)amino)ethyl)amino)hexyl)oxy)phenyl)furan-2-carbonyl)-l-aspartate (13 mg, 0.02 mmol) was hydrolyzed using aqueous 0.6 M LiOH (0.11 mL, 0.07 mmol) as described for **7** and purified by preparative HPLC (0 to 100% mobile phase B (MeCN–H_2_O–TFA 90:10:0.1) in mobile phase A (H_2_O–TFA 100:0.1) over 20 min, flow rate 20 mL/min). The corresponding fractions were combined and concentrated *in vacuo* to give 11 mg (71%) of **17** as a white semisolid (*t*_R_ = 8.04 min, purity 97.9% by HPLC, method A) as a TFA salt; ^1^H NMR (600 MHz, DMSO-*d*_*6*_) δ 8.54–8.50 (m, 1H), 7.84–7.79 (m, 2H), 7.18 (d, *J* = 3.6 Hz, 1H), 7.04–7.01 (m, 2H), 7.00–6.97 (m, 1H), 6.94 (d, *J* = 3.6 Hz, 1H), 4.70–4.63 (m, 1H), 4.03 (t, *J* = 6.4 Hz, 2H), 3.25–3.19 (m, 2H), 2.98–2.89 (m, 4H), 2.87–2.80 (m, 1H), 2.70–2.63 (m, 1H), 1.73 (p, *J* = 6.6 Hz, 2H), 1.60 (p, *J* = 7.6 Hz, 2H), 1.39 (s, 13H); ^13^C NMR (151 MHz, DMSO-*d*_*6*_) δ 172.6, 172.0, 159.0, 157.9 (q, *J* = 30.7 Hz), 157.23, 157.16, 155.7, 155.6, 154.8, 146.0, 145.9, 125.9, 122.1, 117.3 (q, *J* = 301.3 Hz), 116.2, 114.8, 105.9, 78., 67.4, 48.5, 48.4, 46.7, 46.4, 36.5, 36.3, 30.7, 28.4, 28.2, 25.6, 25.3, 25.0; HRMS (MALDI) calcd for C_28_H_39_N_3_O_9_ (M+H)^+^ 562.2759, found 562.2760.

### (*S*)-6-(4-(5-((1,2-Dicarboxyethyl)carbamoyl)furan-2-yl)phenoxy)-*N*-methylhexan-1-aminium 2,2,2-trifluoroacetate (20)

**4** (200 mg, 0.58 mmol) was reacted with K_2_CO_3_ (167 mg, 1.21 mmol), 6-bromohexan-1-ol (113 μL, 0.86 mmol), and KI (15 mg, 0.09 mmol) in MeCN (2.3 mL) as described for **5c**. Purification by flash column chromatography (SiO_2_, 0–100% EtOAc in *n*-heptane) gave 110 mg (43%) of **18** as a colorless sticky solid: *R*_f_ = 0.24 (EtOAc:*n*-heptane, 2:1); ^1^H NMR (400 MHz, CDCl_3_) δ 7.68–7.60 (m, 2H), 7.35 (d, *J* = 8.1 Hz, 1H), 7.19 (d, *J* = 3.6 Hz, 1H), 6.97–6.88 (m, 2H), 6.60 (d, *J* = 3.6 Hz, 1H), 5.10–5.01 (m, 1H), 4.00 (t, *J* = 6.5 Hz, 2H), 3.81 (s, 3H), 3.72 (s, 3H), 3.70 (s, 2H), 3.19–3.10 (m, 1H), 3.03–2.93 (m, 1H), 1.88–1.76 (m, 2H), 1.66–1.39 (m, 6H); ^13^C NMR (101 MHz, CDCl_3_) δ 171.7, 171.3, 159.8, 158.2, 156.3, 145.7, 126.3, 122.4, 117.5, 115.0, 105.9, 68.1, 63.0, 53.1, 52.2, 48.3, 36.4, 32.8, 29.3, 26.0, 25.7; ESI-MS *m*/*z* 448.2 (M+H^+^).

To a flask charged with **18** (110 mg, 0.25 mmol) were added DCM (2.5 mL), Dess–Martin periodinane (126 mg, 0.30 mmol), and NaHCO_3_ (107 mg, 1.27 mmol) at 0 °C. The mixture was stirred for 2 h at 0 °C. After completion, the reaction was quenched with sat. aq. Na_2_S_2_O_3_ and extracted with DCM (×3). The organic phases were combined, washed with brine, dried over MgSO_4_, and concentrated *in vacuo*. The residue was purified by flash column chromatography (SiO_2_, 0–50% EtOAc in *n*-heptane) to give 90 mg (82%) of **19** as a light-yellow oil: *R*_f_ = 0.26 (EtOAc:*n*-heptane, 2:1); ^1^H NMR (400 MHz, CDCl_3_) δ 9.82–9.77 (m, 1H), 7.69–7.61 (m, 2H), 7.34 (d, *J* = 8.1 Hz, 1H), 7.20 (d, *J* = 3.6 Hz, 1H), 6.99–6.89 (m, 2H), 6.60 (d, *J* = 3.6 Hz, 1H), 5.10–5.02 (m, 1H), 4.01 (t, *J* = 6.3 Hz, 2H), 3.81 (s, 3H), 3.73 (s, 3H), 3.20–3.10 (m, 1H), 3.03–2.92 (m, 1H), 2.49 (td, *J* = 7.3, 1.7 Hz, 2H), 1.89–1.79 (m, 2H), 1.73 (p, *J* = 7.4 Hz, 2H), 1.60–1.48 (m, 2H); ^13^C NMR (101 MHz, CDCl_3_) δ 202.5, 171.8, 171.3, 159.7, 158.2, 156.3, 145.8, 126.3, 122.5, 117.5, 115.0, 105.9, 67.9, 53.1, 52.3, 48.4, 43.9, 36.4, 29.2, 25.9, 22.0; [α]^20^_D_ −1.3° (*c* 0.12, MeOH).

(*S*)-6-(4-(5-((1,4-Dimethoxy-1,4-dioxobutan-2-yl)carbamoyl)furan-2-yl)phenoxy)-*N*-methylhexan-1-aminium 2,2,2-trifluoroacetate: To a solution of **19** (25 mg, 0.06 mmol) in anhydrous dichloroethane (1.9 mL) under argon were added methanamine hydrochloride (71 mg, 1.05 mmol) and NEt_3_ (145 μL, 1.04 mmol). The mixture was stirred at rt for 1 h before NaBH(OAc)_3_ (46 mg, 0.22 mmol) was added, and the mixture was stirred at rt for 1 h. After completion, the reaction mixture was diluted with water, extracted with DCM (×3). The organic phases were combined, washed with sat. aq. NaHCO_3_, brine, dried over MgSO_4_, and concentrated *in vacuo*. The residue was purified by preparative HPLC (Gemini-NX C18 column, 0 to 100% mobile phase B (MeCN–H_2_O–TFA 90:10:0.1) in mobile phase A (H_2_O–TFA 100:0.1) over 20 min, flow rate 20 mL/min). The corresponding fractions were combined and lyophilized to give 3.4 mg (11%) of the product as a colorless sticky solid as a TFA salt; ^1^H NMR (600 MHz, CD_3_OD) δ 8.66–8.60 (m, 1H), 7.81–7.75 (m, 2H), 7.21 (d, *J* = 3.6 Hz, 1H), 7.01–6.96 (m, 2H), 6.78 (d, *J* = 3.6 Hz, 1H), 5.05–4.98 (m, 1H), 4.05 (t, *J* = 6.3 Hz, 2H), 3.76 (s, 3H), 3.71 (s, 3H), 3.10–2.92 (m, 4H), 2.70 (s, 3H), 1.88–1.80 (m, 2H), 1.76–1.68 (m, 2H), 1.62–1.54 (m, 2H), 1.53–1.45 (m, 2H); ^13^C NMR (151 MHz, CD_3_OD) δ 172.7, 172.6, 161.2, 158.2, 146.6, 127.3, 123.7, 118.3, 115.9, 106.6, 68.9, 53.2, 52.5, 50.4, 50.2, 36.7, 33.6, 30.0, 27.2, 27.1, 26.7; ESI-MS *m*/*z* 461.2 (M+H+).

(*S*)-6-(4-(5-((1,4-Dimethoxy-1,4-dioxobutan-2-yl)carbamoyl)furan-2-yl)phenoxy)-*N*-methylhexan-1-aminium 2,2,2-trifluoroacetate (3.4 mg, 6 μmol) was hydrolyzed using aqueous 0.6 M LiOH (30 μL, 18 μmol) as described for **7** and purified by preparative HPLC (0 to 100% mobile phase B (MeCN–H_2_O–TFA 90:10:0.1) in mobile phase A (H_2_O–TFA 100:0.1) over 20 min, flow rate 20 mL/min). The corresponding fractions were combined and concentrated *in vacuo* to give 1.4 mg (43%) of **20** as a colorless oil (*t*_R_ = 7.21 min, purity >99% by HPLC, method A) as a TFA salt; ^1^H NMR (600 MHz, CD_3_OD) δ 7.80–7.75 (m, 2H), 7.21 (d, *J* = 3.6 Hz, 1H), 7.01–6.96 (m, 2H), 6.77 (d, *J* = 3.6 Hz, 1H), 4.96 (t, *J* = 6.0 Hz, 1H), 4.05 (t, *J* = 6.3 Hz, 2H), 3.03–2.94 (m, 4H), 2.70 (s, 3H), 1.88–1.80 (m, 2H), 1.76–1.68 (m, 2H), 1.62–1.54 (m, 2H), 1.52–1.45 (m, 2H); ^13^C NMR (151 MHz, CD_3_OD) δ 174.4, 161.1, 160.5, 158.0, 146.8, 127.3, 123.8, 118.1, 115.9, 106.6, 68.8, 50.4, 36.9, 33.6, 30.0, 27.2, 27.1, 26.7; HRMS (MALDI) calcd for C_22_H_28_N_2_O_7_ (M+H)^+^ 433.1969, found 433.1969.

### (*S*)-6-(4-(5-((1,2-Dicarboxyethyl)carbamoyl)furan-2-yl)phenoxy)-*N,N*-dimethylhexan-1-aminium 2,2,2-trifluoroacetate (21)

(*S*)-6-(4-(5-((1,4-Dimethoxy-1,4-dioxobutan-2-yl)carbamoyl)furan-2-yl)phenoxy)-*N,N*-dimethylhexan-1-aminium 2,2,2-trifluoroacetate was synthesized from **19** (19 mg, 0.04 mmol) and dimethylamine hydrochloride (72 mg, 0.88 mmol) in anhydrous dichloroethane (1.9 mL) as described for (*S*)-6-(4-(5-((1,4-dimethoxy-1,4-dioxobutan-2-yl)carbamoyl)furan-2-yl)phenoxy)-*N*-methylhexan-1-aminium 2,2,2-trifluoroacetate. Purification by preparative HPLC (Gemini-NX C18 column, 0 to 100% mobile phase B (MeCN–H_2_O–TFA 90:10:0.1) in mobile phase A (H_2_O–TFA 100:0.1) over 20 min, flow rate 20 mL/min) gave 4.2 mg (17%) of the product as a pale yellow oil as a TFA salt; ^1^H NMR (600 MHz, CDCl_3_) δ 11.55 (s, 1H), 7.68–7.63 (m, 2H), 7.43 (d, *J* = 8.1 Hz, 1H), 7.23 (d, *J* = 3.7 Hz, 1H), 6.94–6.91 (m, 2H), 6.62 (d, *J* = 3.7 Hz, 1H), 5.09–5.03 (m, 1H), 4.00 (t, *J* = 6.2 Hz, 2H), 3.82 (s, 3H), 3.73 (s, 3H), 3.19–3.11 (m, 1H), 3.08–2.96 (m, 3H), 2.84 (d, *J* = 4.6 Hz, 6H), 1.92–1.79 (m, 4H), 1.60–1.43 (m, 4H); ^13^C NMR (151 MHz, CDCl_3_) δ 171.8, 171.2, 159.8, 158.6, 156.6, 145.3, 126.4, 122.5, 118.0, 115.0, 106.1, 67.7, 58.3, 53.2, 52.3, 48.5, 43.2, 36.3, 29.0, 26.4, 25.7, 24.4; ESI-MS *m*/*z* 475.2 (M+H^+^).

(*S*)-6-(4-(5-((1,4-Dimethoxy-1,4-dioxobutan-2-yl)carbamoyl)furan-2-yl)phenoxy)-*N,N*-dimethylhexan-1-aminium 2,2,2-trifluoroacetate (4.2 mg, 7 μmol) was hydrolyzed using aqueous 0.6 M LiOH (37 μL, 22 μmol) as described for **7** and purified by preparative HPLC (0 to 100% mobile phase B (MeCN–H_2_O–TFA 90:10:0.1) in mobile phase A (H_2_O–TFA 100:0.1) over 20 min, flow rate 20 mL/min). The corresponding fractions were combined and concentrated *in vacuo* to give 2.4 mg (60%) of **21** as a colorless oil (*t*_R_ = 7.41 min, 98.7% pure by HPLC, method A) as a TFA salt; ^1^H NMR (600 MHz, CD_3_OD) δ 7.80–7.75 (m, 2H), 7.21 (d, *J* = 3.6 Hz, 1H), 7.01–6.95 (m, 2H), 6.77 (d, *J* = 3.6 Hz, 1H), 4.98–4.93 (m, 1H), 4.05 (t, *J* = 6.3 Hz, 2H), 3.16–3.10 (m, 2H), 3.05–2.93 (m, 2H), 2.88 (s, 6H), 1.88–1.80 (m, 2H), 1.80–1.72 (m, 2H), 1.63–1.55 (m, 2H), 1.52–1.44 (m, 2H); ^13^C NMR (151 MHz, CD_3_OD) δ 161.1, 160.5, 158.0, 146.8, 127.3, 123.8, 118.1, 115.9, 106.6, 68.8, 59.0, 49.6, 43.4, 37.0, 30.0, 27.2, 26.7, 25.6; HRMS (MALDI) calcd for C_23_H_30_N_2_O_7_ (M+H)^+^ 447.2125, found 447.2125.

### (*S*)-6-(4-(5-((1,2-Dicarboxyethyl)carbamoyl)furan-2-yl)phenoxy)-*N*-(2-((7-nitrobenzo[*c*][1,2,5]oxadiazol-4-yl)amino)ethyl)hexan-1-aminium 2,2,2-trifluoroacetate (22)

*tert*-Butyl (2-((7-nitrobenzo[*c*][1,2,5]oxadiazol-4-yl)amino)ethyl)carbamate: To a stirred solution of *tert*-butyl (2-aminoethyl)carbamate (169 μL, 1.07 mmol) in DMF (2 mL) under argon was added Et_3_N (0.15 mL). Then, a solution of NBD-Cl (200 mg, 1.00 mmol) in DMF (1 mL) was added dropwise. The resulting reaction mixture was stirred at rt for 15 h in the dark. After completion, the reaction mixture was diluted with water and extracted with DCM (×4). The organic phases were combined, dried over MgSO_4_, and filtered. The residue was concentrated *in vacuo* and purified by flash column chromatography (SiO2, EtOAc:*n*-heptane, 2:1) to give 290 mg (90%) of the product as a brown solid: *R*_f_ = 0.28 (EtOAc:*n*-heptane, 2:1); ^1^H NMR (400 MHz, CDCl_3_) δ 8.47 (d, *J* = 8.6 Hz, 1H), 7.61 (s, 1H), 6.16 (d, *J* = 8.6 Hz, 1H), 5.07 (s, 1H), 3.66–3.54 (m, 4H), 1.46 (s, 9H); ^13^C NMR (101 MHz, CDCl_3_) δ 144.4, 144.0, 136.6, 81.1, 39.3, 28.4; ESI-MS (method B) *m*/*z* 324.1 (M+H^+^). Spectra in accordance with reported data.^43^

2-((7-Nitrobenzo[*c*][1,2,5]oxadiazol-4-yl)amino)ethan-1-aminium chloride: *tert*-Butyl (2-((7-nitrobenzo[*c*][1,2,5]oxadiazol-4-yl)amino)ethyl)carbamate (278 mg, 0.86 mmol) was Boc-deprotected using 4 M HCl in 1,4-dioxane (1.7 mL) as described for **9a**. The crude was dissolved in EtOAc (1 mL) and precipitated by addition of diethyl ether (9 mL) to give 178 mg (80%) of the product as a brown solid; ^1^H NMR (400 MHz, DMSO-*d*_*6*_) δ 9.36 (br s, 1H), 8.56 (d, *J* = 8.9 Hz, 1H), 8.21 (s, 3H), 6.55 (d, *J* = 8.9 Hz, 1H), 3.80 (s, 2H), 3.19–3.09 (m, 2H). The spectra are in accordance with reported data.^44^

Dimethyl (5-(4-((6-((2-((7-nitrobenzo[*c*][1,2,5]oxadiazol-4-yl)amino)ethyl)amino)hexyl)oxy)phenyl)furan-2-carbonyl)-l-aspartate 2,2,2-trifluoroacetate: To a solution of **19** (27 mg, 0.06 mmol) in THF (0.30 mL) under argon were added 2-((7-nitrobenzo[*c*][1,2,5]oxadiazol-4-yl)amino)ethan-1-aminium chloride (18 mg, 0.07 mmol) and sodium triacetoxyborohydride (19 mg, 0.09 mmol). The reaction mixture was stirred for 23 h at rt in the dark. After completion, the reaction mixture was concentrated and purified by preparative HPLC (0 to 100% mobile phase B (MeCN–H_2_O–TFA 90:10:0.1) in mobile phase A (H_2_O–TFA 100:0.1) over 25 min, flow rate 20 mL/min). The corresponding fractions were combined and lyophilized to give 4 mg (8%) of the product as an orange solid (*t*_R_ = 8.81 min, > 99% pure by HPLC (254 nm), method A) as a TFA salt; ^1^H NMR (600 MHz, CD_3_OD) δ 8.54 (d, *J* = 8.7 Hz, 1H), 7.78–7.72 (m, 2H), 7.21 (d, *J* = 3.6 Hz, 1H), 6.99–6.94 (m, 2H), 6.76 (d, *J* = 3.6 Hz, 1H), 6.44 (d, *J* = 8.7 Hz, 1H), 5.04–4.99 (m, 1H), 4.04 (t, *J* = 6.2 Hz, 2H), 3.93–3.88 (m, 2H), 3.76 (s, 3H), 3.71 (s, 3H), 3.41 (t, *J* = 6.2 Hz, 2H), 3.14–3.10 (m, 2H), 3.09–3.04 (m, 1H), 2.99–2.92 (m, 1H), 1.86–1.80 (m, 2H), 1.80–1.73 (m, 2H), 1.61–1.55 (m, 2H), 1.54–1.48 (m, 2H); ESI-MS *m*/*z* 653.50 (M+H^+^).

Dimethyl (5-(4-((6-((2-((7-nitrobenzo[*c*][1,2,5]oxadiazol-4-yl)amino)ethyl)amino)hexyl)oxy)phenyl)furan-2-carbonyl)-l-aspartate 2,2,2-trifluoroacetate (4 mg, 5 μmol) was dissolved in THF (30 μL), and aqueous 0.6 M LiOH (32 μL, 19 μmol) was added to the mixture. The reaction was stirred at rt for 2 h in the dark, whereupon THF was evaporated, and the mixture was diluted with Milli-Q water and MeCN (∼1 mL) and purified by preparative HPLC (0 to 100% mobile phase B (MeCN–H_2_O–TFA 90:10:0.1) in mobile phase A (H_2_O–TFA 100:0.1) over 25 min, flow rate 20 mL/min). The corresponding fractions were combined and lyophilized to give 3.4 mg (96%) of **22** as an orange solid (*t*_R_ = 8.01 min, purity 98.1% (254 nm) and 95.4% (450 nm) by HPLC, method A); ^1^H NMR (600 MHz, CD_3_OD) δ 8.55 (d, *J* = 8.7 Hz, 1H), 7.78–7.73 (m, 2H), 7.21 (d, *J* = 3.6 Hz, 1H), 7.00–6.93 (m, 2H), 6.76 (d, *J* = 3.6 Hz, 1H), 6.44 (d, *J* = 8.7 Hz, 1H), 4.99–4.94 (m, 1H), 4.04 (t, *J* = 6.2 Hz, 2H), 3.93–3.88 (m, 2H), 3.42 (t, *J* = 6.2 Hz, 2H), 3.15–3.10 (m, 2H), 3.05–2.93 (m, 2H), 1.86–1.80 (m, 2H), 1.80–1.73 (m, 2H), 1.62–1.55 (m, 2H), 1.55–1.47 (m, 2H); HRMS (MALDI) calcd for C_29_H_32_N_6_O_10_ (M+H)^+^ 625.2252, found 625.2254.

### 2-(2-(4′-Chloro-[1,1′-biphenyl]-3-carboxamido)phenyl)acetic acid (32)

Methyl 2-(2-nitrophenyl)acetate: Acetyl chloride (1.2 mL, 16.8 mL) was added dropwise to MeOH (11 mL) at 0 °C under an argon atmosphere. The mixture was stirred for 10 min before portion-wise addition of 2-(2-nitrophenyl)acetic acid (1001 mg, 5.53 mmol). The reaction was allowed to reach rt and stirred for 24 h. Sat. aq. NaHCO_3_ was added, and the mixture was extracted with Et_2_O (×3). The organic phases were combined, washed with brine, dried over MgSO_4_ and concentrated *in vacuo* to give 991 mg (92%) of the product as a brown oil: *R*_f_ = 0.44 (EtOAc:*n*-heptane, 1:1); ^1^H NMR (400 MHz, CDCl_3_) δ 8.12 (dd, *J* = 8.2, 1.4 Hz, 1H), 7.60 (td, *J* = 7.5, 1.4 Hz, 1H), 7.52–7.44 (m, 1H), 7.36 (dd, *J* = 7.6, 1.5 Hz, 1H), 4.03 (s, 2H), 3.71 (s, 3H); ^13^C NMR (101 MHz, CDCl_3_) δ 170.5, 148.9, 133.7, 133.4, 129.8, 128.7, 125.4, 52.4, 39.6. Spectra in accordance with reported data.^45^

Methyl 2-(2-nitrophenyl)acetate (317 mg, 1.62 mmol) was dissolved in MeOH (0.70 mL) under an argon atmosphere. 10% (w/w) Pd/C (17 mg) was added, and the flask was evacuated and backfilled with N_2_ (×3). Then, the flask was evacuated and backfilled with H_2_ (×3). The reaction mixture was stirred for 6 h at rt. After completion, the reaction mixture was filtered through a pad of Celite and concentrated *in vacuo* to give 264 mg (98%) of **23** as a brownish oil: *R*_f_ = 0.47 (EtOAc:*n*-heptane, 1:1); ^1^H NMR (400 MHz, CDCl_3_) δ 7.15–7.06 (m, 2H), 6.79–6.69 (m, 2H), 3.69 (s, 3H), 3.57 (s, 2H); ^13^C NMR (101 MHz, CDCl_3_) δ 172.4, 145.6, 131.3, 128.7, 119.5, 119.1, 116.7, 52.3, 38.4; ESI-MS *m*/*z* 166.1 (M+H^+^). Spectra in accordance with reported data.^45^

**24** (380 mg, 1.89 mmol) was dissolved in DMF (2.0 mL) in a dry flask. Then, *N,N*-diisopropylethylamine (0.69 mL, 3.96 mmol) was added, followed by HATU (716 mg, 1.88 mmol). The reaction mixture was stirred at rt for 30 min before **30** (260 mg, 1.57 mmol) dissolved in DMF (1.1 mL) was added. The reaction was stirred at rt for 40 h. After completion, reaction mixture was diluted with water, extracted with EtOAc (×3), washed with brine, dried over MgSO_4_, filtered, and concentrated *in vacuo*. The residue was purified by flash column chromatography (SiO_2_, 0–20% EtOAc in *n*-heptane) to give 530 mg (97%) of **28** as a pale yellow solid: *R*_f_ = 0.39 (EtOAc:*n*-heptane, 1:2); ^1^H NMR (400 MHz, CDCl_3_) δ 9.71 (s, 1H), 8.22–8.15 (m, 1H), 8.00 (d, *J* = 8.1 Hz, 1H), 7.97–7.92 (m, 1H), 7.71–7.64 (m, 1H), 7.42–7.31 (m, 2H), 7.28–7.21 (m, 1H), 7.21–7.09 (m, 1H), 3.76 (s, 3H), 3.69 (s, 2H); ^13^C NMR (101 MHz, CDCl_3_) δ 173.7, 164.2, 136.8, 136.7, 134.9, 131.04, 130.95, 130.4, 128.7, 125.8, 125.8, 125.7, 125.0, 123.1, 52.9, 39.0; ESI-MS *m*/*z* 348.18 (M+H^+^). Spectra in accordance with reported data.^46^

Methyl 2-(2-(4′-chloro-[1,1′-biphenyl]-3-carboxamido)phenyl)acetate: A two-neck round-bottom flask under an argon atmosphere was charged with (4-chlorophenyl)boronic acid (50 mg, 0.32 mmol) and PdCl_2_(PPh_3_)_2_ (10 mg, 5 mol%). The flask was evacuated and backfilled with argon (×3). Afterward, **28** (99 mg, 0.28 mmol) dissolved in toluene (0.60 mL), aqueous 1 M Na_2_CO_3_ (0.30 mL), EtOH (0.15 mL), and additional toluene (0.50 mL) were added to the flask. The mixture was heated to 80 °C overnight (17 h). The resulting reaction mixture was diluted with EtOAc and poured into water. The aqueous phase was extracted with EtOAc (×3), washed with brine, dried over Na_2_SO_4_, filtered, and concentrated *in vacuo*. The residue was purified by flash column chromatography (SiO_2_, 0–30% EtOAc in *n*-heptane) to give 73 mg (68%) of the product as a thick pale yellow oil: *R*_f_ = 0.25 (EtOAc:*n*-heptane, 1:2); ^1^H NMR (400 MHz, CDCl_3_) δ 9.84 (s, 1H), 8.28 (s, 1H), 8.07 (d, *J* = 8.2 Hz, 1H), 8.04–8.00 (m, 1H), 7.77–7.73 (m, 1H), 7.66–7.54 (m, 3H), 7.48–7.40 (m, 2H), 7.43–7.34 (m, 1H), 7.30–7.23 (m, 1H), 7.20–7.12 (m, 1H), 3.76 (s, 3H), 3.71 (s, 2H); ^13^C NMR (10 Hz, CDCl_3_) δ 173.8, 165.5, 140.6, 138.8, 137.1, 135.4, 134.1, 131.1, 130.3, 129.5, 129.2, 128.7, 128.6, 126.4, 126.3, 125.7, 125.5, 124.9, 52.9, 39.1; ESI-MS *m*/*z* 380.4 (M+H^+^).

Methyl 2-(2-(4′-chloro-[1,1′-biphenyl]-3-carboxamido)phenyl)acetate (70 mg, 0.18 mmol) was hydrolyzed using aqueous 0.6 M LiOH (0.90 mL) as described for **7** to give 60 mg (89%) of **32** as a pale yellow solid (*t*_R_ = 10.66 min, purity 97.5% by HPLC, method A); ^1^H NMR (400 MHz, DMSO-*d*_*6*_) δ 12.36 (br s, 1H), 10.16 (s, 1H), 8.24 (s, 2H), 7.94 (d, *J* = 7.7 Hz, 2H), 7.90 (d, *J* = 7.8 Hz, 2H), 7.81 (d, *J* = 8.3 Hz, 3H), 7.63 (t, *J* = 7.7 Hz, 2H), 7.57 (d, *J* = 8.4 Hz, 3H), 7.52–7.45 (m, 2H), 7.38–7.27 (m, 3H), 7.27–7.19 (m, 2H), 3.69 (s, 2H); ^13^C NMR (10 Hz, DMSO-*d*_*6*_) δ 172.7, 165.1, 138.9, 138.3, 136.6, 135.3, 132.8, 131.0, 130.9, 129.6, 129.2, 129.0, 128.7, 127.2, 127.1, 126.4, 125.9, 125.7, 37.7; HRMS (MALDI) calcd for C_21_H_16_ClNO_3_ (M+H)^+^ 366.0891, found 366.0889. Spectra in accordance with reported data.^27^

### 2-(2-(4′-Chloro-2-methyl-[1,1′-biphenyl]-3-carboxamido)phenyl)acetic acid (33)

**25** (82 mg, 0.38 mmol) was coupled to **23** (52 mg, 0.31 mmol) as described for **28**, and purification by flash column chromatography (SiO_2_, 0–17% EtOAc in *n*-heptane) gave 46 mg (40%) of **29** as a white solid: *R*_f_ = 0.48 (EtOAc:*n*-heptane, 1:1); ^1^H NMR (400 MHz, CDCl_3_) δ 9.03 (s, 1H), 8.03 (d, *J* = 8.0 Hz, 1H), 7.70–7.63 (m, 1H), 7.51 (d, *J* = 7.6 Hz, 1H), 7.43–7.34 (m, 1H), 7.28–7.22 (m, 1H), 7.21–7.11 (m, 2H), 3.68 (s, 3H), 3.66 (s, 2H), 2.60 (s, 3H); ^13^C NMR (101 MHz, CDCl_3_) δ 173.1, 167.8, 138.8, 136.6, 136.3, 134.5, 131.1, 128.8, 127.4, 127.1, 126.03, 125.98, 125.9, 124.9, 52.8, 38.9, 20.3; ESI-MS *m*/*z* 362.1 (M+H^+^).

Methyl 2-(2-(4′-chloro-2-methyl-[1,1′-biphenyl]-3-carboxamido)phenyl)acetate: **29** (45 mg, 0.12 mmol) was coupled to (4-chlorophenyl)boronic acid (21 mg, 0.14 mmol) as described for methyl 2-(2-(4′-chloro-[1,1′-biphenyl]-3-carboxamido)phenyl)acetate, and purification by flash column chromatography (SiO_2_, 0–20% EtOAc in *n*-heptane) gave 34 mg (70%) of methyl 2-(2-(4′-chloro-2-methyl-[1,1′-biphenyl]-3-carboxamido)phenyl)acetate as a white solid: *R*_f_ = 0.26 (EtOAc:*n*-heptane, 1:3); ^1^H NMR (400 MHz, CDCl_3_) δ 9.01 (s, 1H), 8.04 (d, *J* = 8.0 Hz, 1H), 7.60–7.54 (m, 1H), 7.42–7.22 (m, 8H), 7.19–7.14 (m, 1H), 3.71–3.63 (m, 5H), 2.38 (s, 3H); ^13^C NMR (101 MHz, CDCl_3_) δ 173.1, 168.8, 142.6, 140.0, 137.9, 136.8, 133.9, 133.4, 131.8, 131.1, 130.8, 128.7, 128.6, 126.3, 126.1, 126.0, 125.8, 125.0, 52.8, 38.8, 17.9; ^13^C NMR (101 MHz, CDCl_3_) δ 173.1, 168.8, 142.6, 140.0, 137.9, 136.8, 133.9, 133.4, 131.8, 131.1, 130.8, 128.7, 128.6, 126.3, 126.1, 126.0, 125.8, 125.0, 52.8, 38.8, 17.9; ESI-MS *m*/*z* 394.46 (M+H^+^).

Methyl 2-(2-(4′-chloro-2-methyl-[1,1′-biphenyl]-3-carboxamido)phenyl)acetate (17 mg, 0.04 mmol) was hydrolyzed using aqueous 0.6 M LiOH (0.22 mL, 0.13 mmol) as described for **7** to give 15 mg (92%) of **33** as a white solid (*t*_R_ = 10.56 min, purity 95.5% by HPLC, method A); ^1^H NMR (600 MHz, CD_3_OD) δ 7.60–7.53 (m, 2H), 7.47–7.43 (m, 2H), 7.38–7.29 (m, 6H), 7.29–7.24 (m, 1H), 3.75 (s, 2H), 2.35 (s, 3H); ^13^C NMR (151 MHz, CD_3_OD) δ 175.2, 171.9, 143.4, 141.4, 139.3, 137.2, 134.4, 134.1, 132.35, 132.33, 131.9, 131.7, 129.5, 128.9, 127.8, 127.61, 127.60, 127.5, 126.9, 38.8, 17.8; HRMS (MALDI) calcd for C_22_H_18_ClNO_3_ (M+H)^+^ 380.1048, found 380.1044.

### 2-(2-(4′-Chloro-6-methyl-[1,1′-biphenyl]-3-carboxamido)phenyl)acetic acid (34)

**26** (47 mg, 0.22 mmol) was coupled to **23** (30 mg, 0.18 mmol) as described for **28**, and purification by flash column chromatography (SiO_2_, 0–15% EtOAc in *n*-heptane) gave 34 mg (52%) of **30** as a white solid: *R*_f_ = 0.62 (EtOAc:*n*-heptane, 1:1); ^1^H NMR (400 MHz, CDCl_3_) δ 9.64 (s, 1H), 8.24–8.19 (m, 1H), 8.00 (d, *J* = 8.1 Hz, 1H), 7.89–7.82 (m, 1H), 7.41–7.32 (m, 2H), 7.28–7.22 (m, 1H), 7.19–7.11 (m, 1H), 3.77 (s, 3H), 3.69 (s, 2H), 2.47 (s, 3H); ^13^C NMR (101 MHz, CDCl_3_) δ 173.6, 164.2, 142.2, 136.9, 134.0, 131.8, 131.1, 131.0, 128.7, 126.0, 125.8, 125.6, 125.4, 125.0, 52.9, 39.0, 23.2; ESI-MS *m*/*z* 362.1 (M+H^+^).

**30** (34 mg, 0.09 mmol) was coupled to (4-chlorophenyl)boronic acid (16 mg, 0.10 mmol) as described for methyl 2-(2-(4′-chloro-[1,1′-biphenyl]-3-carboxamido)phenyl)acetate, and purification by flash column chromatography (SiO_2_, 0–13% EtOAc in *n*-heptane) gave 10 mg (27%) of methyl 2-(2-(4′-chloro-6-methyl-[1,1′-biphenyl]-3-carboxamido)phenyl)acetate as a colorless oil: *R*_f_ = 0.55 (EtOAc:*n*-heptane, 1:2); ^1^H NMR (600 MHz, CDCl_3_) δ 9.65 (s, 1H), 8.04 (d, *J* = 8.1 Hz, 1H), 7.94–7.87 (m, 2H), 7.44–7.40 (m, 3H), 7.38–7.34 (m, 1H), 7.33–7.29 (m, 2H), 7.26–7.23 (m, 1H), 7.16–7.12 (m, 1H), 3.72 (s, 3H), 3.69 (s, 2H), 2.34 (s, 3H); ^13^C NMR (151 MHz, CDCl_3_) δ 173.6, 165.5, 141.2, 139.8, 139.6, 137.2, 133.5, 132.6, 131.05, 131.02, 130.7, 129.1, 128.7, 128.6, 126.3, 125.7, 125.4, 125.0, 52.9, 39.1, 20.7; ESI-MS *m*/*z* 394.2 (M+H^+^).

Methyl 2-(2-(4′-chloro-6-methyl-[1,1′-biphenyl]-3-carboxamido)phenyl)acetate (10 mg, 0.03 mmol) was hydrolyzed using aqueous 0.6 M LiOH (0.48 mL, 0.29 mmol) as described for **7** to give 9 mg (95%) of **34** as a white solid (*t*_R_ = 10.81 min, purity 96.8% by HPLC, method A); ^1^H NMR (600 MHz, CD_3_OD) δ 7.91–7.86 (m, 1H), 7.85–7.82 (m, 1H), 7.61–7.57 (m, 1H), 7.49–7.42 (m, 3H), 7.40–7.31 (m, 4H), 7.26–7.20 (m, 1H), 3.70 (s, 2H), 2.33 (s, 3H); ^13^C NMR (151 MHz, CD_3_OD) δ 175.8, 168.4, 142.3, 141.1, 141.0, 137.7, 134.5, 133.3, 132.2, 131.91, 131.86, 131.3, 129.9, 129.5, 128.9, 127.9, 127.5, 127.3, 39.2, 20.6; HRMS (MALDI) calcd for C_22_H_18_ClNO_3_ (M+H)^+^ 380.1048, found 380.1046.

### 2-(2-(4′-Chloro-2′-methyl-[1,1′-biphenyl]-3-carboxamido)phenyl)acetic acid (35)

**28** (30 mg, 0.09 mmol) was coupled to (4-chloro-2-methylphenyl)boronic acid (17 mg, 0.10 mmol) as described for methyl 2-(2-(4′-chloro-[1,1′-biphenyl]-3-carboxamido)phenyl)acetate, and purification by flash column chromatography (SiO_2_, 0–10% EtOAc in *n*-heptane) gave 21 mg (61%) of methyl 2-(2-(4′-chloro-2′-methyl-[1,1′-biphenyl]-3-carboxamido)phenyl)acetate as a colorless oil: *R*_*f*_ = 0.49 (EtOAc:*n-*heptane, 1:2); ^1^H NMR (400 MHz, CDCl_3_) δ 9.75 (s, 1H), 8.08–7.96 (m, 3H), 7.60–7.54 (m, 1H), 7.50–7.46 (m, 1H), 7.40–7.35 (m, 1H), 7.31–7.12 (m, 5H), 3.73 (s, 3H), 3.70 (s, 2H), 2.29 (s, 3H); ^13^C NMR (101 MHz, CDCl_3_) δ 173.7, 165.6, 141.5, 139.6, 137.5, 137.1, 134.9, 133.5, 132.7, 131.2, 131.0, 130.4, 128.8, 128.7, 128.4, 126.2, 126.0, 125.8, 125.5, 125.1, 52.9, 39.1, 20.5; ESI-MS *m*/*z* 394.26 (M+H^+^).

Methyl 2-(2-(4′-chloro-2′-methyl-[1,1′-biphenyl]-3-carboxamido)phenyl)acetate (21 mg, 0.05 mmol) was hydrolyzed using aqueous 0.6 M LiOH (1.0 mL, 0.60 mmol) as described for **7** and purified by preparative HPLC (50 to 100% mobile phase B (MeCN–H_2_O–TFA 90:10:0.1) in mobile phase A (H_2_O–TFA 100:0.1) over 10 min, flow rate 20 mL/min). HPLC fractions were combined and lyophilized to give 16 mg (81%) of **35** as a white fluffy solid (*t*_R_ = 10.86 min, purity >99% by HPLC, method A); ^1^H NMR (400 MHz, CDCl_3_) δ 9.28 (s, 1H), 7.96–7.86 (m, 3H), 7.53–7.45 (m, 2H), 7.39–7.32 (m, 1H), 7.29–7.22 (m, 3H), 7.21–7.12 (m, 3H), 3.67 (s, 2H), 2.23 (s, 3H); ^13^C NMR (101 MHz, CDCl_3_) δ 176.6, 166.1, 141.6, 139.4, 137.4, 136.4, 134.4, 133.5, 132.9, 131.2, 131.1, 130.4, 128.92, 128.90, 128.2, 126.3, 126.20, 126.17, 126.1, 125.6, 38.6, 20.5; HRMS (MALDI) calcd for C_22_H_18_ClNO_3_ (M+H)^+^ 380.1048, found 380.1048.

### 2-(2-(4′-((6-((*tert*-Butoxycarbonyl)amino)hexyl)oxy)-[1,1′-biphenyl]-3-carboxamido)phenyl)acetic acid (38)

A Schlenk flask was charged with **28** (166 mg, 0.48 mmol), (4-hydroxyphenyl)boronic acid (80 mg, 0.58 mmol), and XPhos-Pd-G4 (9 mg, 2 mol%) under an argon atmosphere. The flask was evacuated and backfilled with argon (×3). Afterward, THF (2.4 mL) and degassed aqueous 0.5 M K_3_PO_4_ (1.9 mL, 0.95 mmol) were added. The mixture was stirred at 50 °C for 22 h. After completion, the reaction mixture was cooled to rt, diluted with water, and extracted with EtOAc (×3). The organic phases were combined, washed with brine, dried over MgSO_4_, filtered, and concentrated *in vacuo*. The residue was purified by flash column chromatography (SiO_2_, 0–30% EtOAc in *n*-heptane) to give 120 mg (70%) of **36** as a light brown solid: *R*_f_ = 0.38 (EtOAc:*n*-heptane, 1:1); ^1^H NMR (400 MHz, DMSO-*d*_*6*_) δ 10.05 (s, 1H), 9.61 (s, 1H), 8.15–8.10 (m, 1H), 7.85–7.76 (m, 2H), 7.63–7.52 (m, 3H), 7.45–7.39 (m, 1H), 7.39–7.29 (m, 2H), 7.25 (td, *J* = 7.4, 1.5 Hz, 1H), 6.93–6.84 (m, 2H), 3.77 (s, 2H), 3.50 (s, 3H); ^13^C NMR (151 MHz, DMSO-*d*_*6*_) δ 171.5, 165.5, 157.5, 140.3, 136.6, 135.0, 131.0, 130.8, 130.2, 129.0, 128.9, 127.9, 127.4, 126.8, 126.1, 125.7, 125.0, 115.8, 51.6, 40.1, 37.2; ESI-MS *m*/*z* 362.1 (M+H^+^).

Methyl 2-(2-(4′-((6-((*tert*-butoxycarbonyl)amino)hexyl)oxy)-[1,1′-biphenyl]-3-carboxamido)phenyl)acetate: **36** (25 mg, 0.07 mmol) was reacted with K_2_CO_3_ (20 mg, 0.14 mmol) and 6-((*tert*-butoxycarbonyl)amino)hexyl 4-methylbenzenesulfonate (38 mg, 0.10 mmol) in a mixture of MeCN (0.40 mL) and DMF (0.15 mL) as described for **5c**. Purification by flash column chromatography (SiO2, 0–30% EtOAc in *n*-heptane) gave 21 mg (54%) of methyl 2-(2-(4′-((6-((*tert*-butoxycarbonyl)amino)hexyl)oxy)-[1,1′-biphenyl]-3-carboxamido)phenyl)acetate as a light-pink semisolid: *R*_f_ = 0.46 (EtOAc:*n*-heptane, 1:1); ^1^H NMR (600 MHz, CDCl_3_) δ 9.75 (s, 1H), 8.28–8.24 (m, 1H), 8.07 (d, *J* = 8.1 Hz, 1H), 7.98–7.92 (m, 1H), 7.77–7.72 (m, 1H), 7.64–7.59 (m, 2H), 7.55 (t, *J* = 7.7 Hz, 1H), 7.41–7.35 (m, 1H), 7.28–7.24 (m, 1H), 7.18–7.13 (m, 1H), 7.01–6.96 (m, 2H), 4.51 (br s, 1H), 4.01 (t, *J* = 6.4 Hz, 2H), 3.76 (s, 3H), 3.73–3.69 (m, 2H), 3.16–3.10 (m, 2H), 1.85–1.78 (m, 2H), 1.55–1.37 (m, 15H); ^13^C NMR (151 MHz, CDCl_3_) δ 173.7, 165.8, 159.2, 156.2, 141.5, 137.2, 135.2, 132.7, 131.0, 130.1, 129.3, 128.7, 128.4, 125.9, 125.8, 125.4, 125.0, 115.0, 79.2, 68.1, 52.9, 40.7, 39.1, 30.2, 29.3, 28.6, 26.7, 25.9; ESI-MS *m*/*z* 561.2 (M+H+).

Methyl 2-(2-(4′-((6-((*tert*-butoxycarbonyl)amino)hexyl)oxy)-[1,1′-biphenyl]-3-carboxamido)phenyl)acetate (14 mg, 0.03 mmol) was hydrolyzed using aqueous 0.6 M LiOH (83 μL, 0.05 mmol) as described for **7**. Purification by flash column chromatography (SiO_2_, DCM→ EtOAc:*n*-heptane, 1:2 → 1:1 → EtOAc → EtOAc [1% AcOH]) gave 8 mg (56%) of **38** as a white solid (*t*_R_ = 11.37 min, purity 97.4% by HPLC, method A); ^1^H NMR (600 MHz, DMSO-*d*_6_) δ 12.34 (br s, 1H), 10.09 (s, 1H), 8.21–8.17 (m, 1H), 7.89–7.80 (m, 2H), 7.73–7.67 (m, 2H), 7.57 (t, *J* = 7.7 Hz, 1H), 7.48 (d, *J* = 7.9 Hz, 1H), 7.37–7.29 (m, 2H), 7.26–7.20 (m, 1H), 7.08–7.01 (m, 2H), 6.79–6.74 (m, 1H), 4.02 (t, *J* = 6.5 Hz, 2H), 3.70–3.65 (m, 2H), 2.92 (q, *J* = 6.6 Hz, 2H), 1.73 (p, *J* = 6.7 Hz, 2H), 1.49–1.28 (m, 15H); ^13^C NMR (151 MHz, DMSO-*d*_6_) δ 172.7, 165.3, 158.6, 155.6, 139.9, 136.7, 135.1, 131.6, 131.0, 130.8, 129.1, 129.0, 128.0, 127.2, 126.3, 126.0, 125.8, 125.2, 114.9, 77.3, 67.5, 40.1, 37.7, 29.4, 28.6, 28.3, 26.0, 25.2; HRMS (MALDI) calcd for C_32_H_38_N_2_O_6_ (M+H)^+^ 547.2802, found 547.2809.

### 6-((3′-((2-(Carboxymethyl)phenyl)carbamoyl)-[1,1′-biphenyl]-4-yl)oxy)hexan-1-aminium 2,2,2-trifluoroacetate (39)

Methyl 2-(2-(4′-((6-((*tert*-butoxycarbonyl)amino)hexyl)oxy)-[1,1′-biphenyl]-3-carboxamido)phenyl)acetate (6 mg, 0.01 mmol) was dissolved in THF (0.20 mL). Then, aqueous 4 M HCl (60 μL) was added. The reaction mixture was heated to 55 °C and stirred for 15 h. After completion, the reaction mixture was concentrated *in vacuo* and purified by preparative HPLC (0 to 100% mobile phase B (MeCN–H_2_O–TFA 90:10:0.1) in mobile phase A (H_2_O–TFA 100:0.1) over 20 min, flow rate 20 mL/min). The corresponding fractions were combined and concentrated *in vacuo* to give 4 mg (67%) of **39** as a white solid of the TFA salt (*t*_R_ = 8.28 min, purity >99% by HPLC, method A); ^1^H NMR (600 MHz, CD_3_OD) δ 8.21 (s, 1H), 7.91 (d, *J* = 7.7 Hz, 1H), 7.83–7.78 (m, 1H), 7.67–7.62 (m, 3H), 7.57 (t, *J* = 7.7 Hz, 1H), 7.40–7.33 (m, 2H), 7.28–7.22 (m, 1H), 7.05–6.99 (m, 2H), 4.06 (t, *J* = 6.3 Hz, 2H), 3.75–3.72 (m, 2H), 2.97–2.91 (m, 2H), 1.88–1.81 (m, 2H), 1.75–1.66 (m, 2H), 1.62–1.54 (m, 2H), 1.53–1.45 (m, 2H); ^13^C NMR (151 MHz, CD_3_OD) δ 176.0, 168.7, 160.5, 142.7, 137.7, 136.2, 133.8, 132.2, 131.3, 131.0, 130.2, 129.2, 128.9, 127.5, 127.2, 126.7, 116.0, 68.8, 40.7, 39.5, 30.1, 28.6, 27.2, 26.7; HRMS (MALDI) calcd for C_27_H_30_N_2_O_4_ (M+H)^+^ 447.2278, found 447.2278.

### 6-((3′-((2-(Carboxymethyl)phenyl)carbamoyl)-2-methyl-[1,1′-biphenyl]-4-yl)oxy)hexan-1-aminium chloride (40)

**37** was synthesized from (4-hydroxy-2-methylphenyl)boronic acid (84 mg, 0.55 mmol) and methyl 2-(2-(3-bromobenzamido)phenyl)acetate (160 mg, 0.46 mmol) as described for **36**. Purification by flash column chromatography (SiO_2_, 0–50% EtOAc in *n*-heptane) gave 130 mg (75%) of **37** as a light-brown solid: *R*_f_ = 0.16 (EtOAc:*n*-heptane, 1:2); ^1^H NMR (400 MHz, CDCl_3_) δ 9.75 (s, 1H), 8.06–7.92 (m, 3H), 7.58–7.46 (m, 2H), 7.42–7.34 (m, 1H), 7.29–7.23 (m, 1H), 7.21–7.13 (m, 1H), 7.09 (d, *J* = 8.2 Hz, 1H), 6.79–6.77 (m, 1H), 6.76–6.72 (m, 1H), 3.74 (s, 3H), 3.71 (s, 2H), 2.22 (s, 3H); ^13^C NMR (101 MHz, CDCl_3_) δ 173.7, 166.3, 155.6, 142.6, 137.0, 136.9, 134.4, 133.4, 133.2, 131.2, 131.0, 129.0, 128.7, 128.6, 126.1, 125.7, 125.3, 125.2, 117.3, 113.1, 52.9, 39.0, 20.6; ESI-MS *m*/*z* 376.2 (M+H^+^).

**37** (31 mg, 0.08 mmol) was reacted with K_2_CO_3_ (23 mg, 0.17 mmol) and 6-((*tert*-butoxycarbonyl)amino)hexyl 4-methylbenzenesulfonate (60 mg, 0.16 mmol) in DMF (0.32 mL) at 80 °C as described for **5c**. Purification by flash column chromatography (SiO_2_, EtOAc:*n*-heptane, 1:3) gave 20 mg (43%) of methyl 2-(2-(4′-((6-((*tert*-butoxycarbonyl)amino)hexyl)oxy)-2′-methyl-[1,1′-biphenyl]-3-carboxamido)phenyl)acetate as a dark red oil: *R*_f_ = 0.51 (EtOAc:*n*-heptane, 1:1); ^1^H NMR (400 MHz, CDCl_3_) δ 9.67 (s, 1H), 8.07–8.00 (m, 1H), 8.00–7.93 (m, 2H), 7.58–7.45 (m, 2H), 7.41–7.33 (m, 1H), 7.25–7.23 (m, 1H), 7.21–7.11 (m, 2H), 6.86–6.76 (m, 2H), 4.50 (br s, 1H), 3.99 (t, *J* = 6.4 Hz, 2H), 3.73 (s, 3H), 3.70 (s, 2H), 3.17–3.09 (m, 2H), 2.30 (s, 3H), 1.86–1.75 (m, 2H), 1.59–1.34 (m, 15H); ^13^C NMR (101 MHz, CDCl_3_) δ 173.5, 165.8, 158.7, 142.5, 137.1, 136.9, 134.6, 133.6, 133.1, 131.1, 131.0, 128.7, 128.7, 128.6, 125.9, 125.5, 125.4, 125.2, 116.6, 111.9, 67.9, 52.9, 39.0, 30.2, 29.4, 28.6, 26.7, 26.0, 20.9; ESI-MS *m*/*z* 575.3 (M+H^+^).

Methyl 2-(2-(4′-((6-((*tert*-butoxycarbonyl)amino)hexyl)oxy)-2′-methyl-[1,1′-biphenyl]-3-carboxamido)phenyl)acetate (20 mg, 0.03 mmol) was hydrolyzed using aqueous 0.6 M LiOH (0.17 mL, 0.10 mmol) as described for **7** and purified by preparative HPLC (50 to 100% mobile phase B (MeCN–H_2_O–TFA 90:10:0.1) in mobile phase A (H_2_O–TFA 100:0.1) over 20 min, flow rate 20 mL/min). The corresponding fractions were combined, concentrated *in vacuo*, and lyophilized to give 9 mg (45%) of 2-(2-(4′-((6-((*tert*-butoxycarbonyl)amino)hexyl)oxy)-2′-methyl-[1,1′-biphenyl]-3-carboxamido)phenyl)acetic acid as a white solid (*t*_R_ = 8.11 min, purity >99% by HPLC, method B); ^1^H NMR (400 MHz, CD_3_OD) δ 7.97–7.88 (m, 2H), 7.65–7.48 (m, 3H), 7.39–7.30 (m, 2H), 7.29–7.20 (m, 1H), 7.16 (d, *J* = 8.4 Hz, 1H), 6.87–6.77 (m, 2H), 4.00 (t, *J* = 6.4 Hz, 2H), 3.72 (s, 2H), 3.05 (t, *J* = 6.9 Hz, 2H), 1.86–1.74 (m, 2H), 1.57–1.37 (m, 15H); ^13^C NMR (151 MHz, CD_3_OD) δ 175.7, 168.7, 160.1, 158.6, 143.8, 137.8, 137.7, 135.5, 134.8, 134.1, 132.2, 131.8, 131.3, 129.54, 129.52, 129.0, 127.5, 127.4, 126.8, 117.4, 113.0, 79.8, 68.9, 41.3, 39.1, 30.9, 30.4, 28.8, 27.6, 26.9, 20.9; HRMS (MALDI) calcd for C_33_H_40_N_2_O_6_ (M+Na)^+^ 583.2778, found 583.2782.

2-(2-(4′-((6-((*tert*-Butoxycarbonyl)amino)hexyl)oxy)-2′-methyl-[1,1′-biphenyl]-3-carboxamido)phenyl)acetic acid (6 mg, 0.01 mmol) was Boc-deprotected using 4 M HCl in 1,4-dioxane (50 μL, 0.02 mmol) as described for **9a** to give 5.5 mg (quant.) of **40** as a colorless oil (*t*_R_ = 8.40 min, purity 97.4% by HPLC, method A); ^1^H NMR (600 MHz, CD_3_OD) δ 7.95 (d, *J* = 7.6 Hz, 1H), 7.90 (s, 1H), 7.63 (d, *J* = 7.9 Hz, 1H), 7.58–7.54 (m, 1H), 7.53–7.50 (m, 1H), 7.37–7.32 (m, 2H), 7.27–7.21 (m, 1H), 7.17 (d, *J* = 8.3 Hz, 1H), 6.86–6.84 (m, 1H), 6.83–6.79 (m, 1H), 4.03 (t, *J* = 6.3 Hz, 2H), 3.72 (br s, 2H), 2.97–2.92 (m, 2H), 2.26 (s, 3H), 1.87–1.79 (m, 2H), 1.75–1.67 (m, 2H), 1.62–1.54 (m, 2H), 1.54–1.45 (m, 2H); ^13^C NMR (151 MHz, CD_3_OD) δ 168.7, 160.0, 143.8, 137.8, 137.7, 135.5, 134.9, 134.1, 132.2, 131.9, 131.3, 129.6, 129.5, 128.9, 127.5, 127.3, 126.7, 117.4, 113.0, 68.7, 40.7, 39.4, 30.2, 28.6, 27.2, 26.7, 20.9; HRMS (MALDI) calcd for C_28_H_32_N_2_O_4_ (M+H)^+^ 461.2435, found 461.2436.

### 5-((3′-((2-(Carboxymethyl)phenyl)carbamoyl)-2-methyl-[1,1′-biphenyl]-4-yl)oxy)pentan-1-aminium chloride (41)

**37** (28 mg, 0.07 mmol) was reacted with K_2_CO_3_ (31 mg, 0.19 mmol) and 5-((*tert*-butoxycarbonyl)amino)pentyl 4-methylbenzenesulfonate (104 mg, 0.29 mmol) in DMF (0.30 mL) at 80 °C as described for **5c**. Purification by flash column chromatography (SiO_2_, 10–30% EtOAc in *n*-heptane) gave 17 mg of methyl 2-(2-(4′-((5-((*tert-*butoxycarbonyl)amino)pentyl)oxy)-2′-methyl-[1,1′-biphenyl]-3-carboxamido)phenyl)acetate as a pale-pink oil that was used directly in the next step: ESI-MS *m*/*z* 561.3 (M+H^+^).

2-(2-(4′-((5-((*tert*-Butoxycarbonyl)amino)pentyl)oxy)-2′-methyl-[1,1′-biphenyl]-3-carboxamido)phenyl)acetic acid: Methyl 2-(2-(4′-((5-((*tert*-butoxycarbonyl)amino)pentyl)oxy)-2′-methyl-[1,1′-biphenyl]-3-carboxamido)phenyl)acetate (17 mg, 0.03 mmol) was hydrolyzed using aqueous 0.6 M LiOH (0.15 mL, 0.09 mmol) as described for **7** and purified by preparative HPLC (50 to 100% mobile phase B (MeCN–H_2_O–TFA 90:10:0.1) in mobile phase A (H_2_O–TFA 100:0.1) over 20 min, flow rate 20 mL/min). The corresponding fractions were combined, concentrated *in vacuo*, and lyophilized to give 9 mg (23% over two steps) of the product as a colorless oil (*t*_R_ = 7.54 min, purity 97.7% by HPLC, method B); ^1^H NMR (600 MHz, CD_3_OD) δ 7.96–7.89 (m, 2H), 7.61 (d, *J* = 7.8 Hz, 1H), 7.57–7.49 (m, 2H), 7.38–7.32 (m, 2H), 7.27–7.22 (m, 1H), 7.16 (d, *J* = 8.4 Hz, 1H), 6.87–6.83 (m, 1H), 6.82–6.79 (m, 1H), 4.01 (t, *J* = 6.4 Hz, 2H), 3.72 (s, 2H), 3.07 (t, *J* = 6.6 Hz, 2H), 2.26 (s, 3H), 1.81 (p, *J* = 6.6 Hz, 2H), 1.60–1.47 (m, 4H), 1.44 (s, 9H); ^13^C NMR (151 MHz, CD_3_OD) δ 175.7, 168.7, 160.1, 158.6, 143.8, 137.8, 137.7, 135.5, 134.8, 134.1, 132.2, 131.8, 131.3, 129.54, 129.53, 129.0, 127.5, 127.4, 126.8, 117.4, 113.0, 79.8, 68.8, 41.3, 39.1, 30.8, 30.1, 28.8, 24.4, 20.9; HRMS (MALDI) calcd for C_32_H_38_N_2_O_6_ (M+Na)^+^ 569.2622, found 569.2629.

2-(2-(4′-((5-((*tert*-Butoxycarbonyl)amino)pentyl)oxy)-2′-methyl-[1,1′-biphenyl]-3-carboxamido)phenyl)acetic acid (6 mg, 0.01 mmol) was Boc-deprotected using 4 M HCl in 1,4-dioxane (50 μL, 0.02 mmol) as described for **9a** to give 5.3 mg (quant.) of **41** as a colorless oil (*t*_R_ = 8.20 min, purity 95.1% by HPLC, method A); ^1^H NMR (600 MHz, CD_3_OD) δ 7.97–7.92 (m, 1H), 7.91 (s, 1H), 7.63 (d, *J* = 7.9 Hz, 1H), 7.59–7.53 (m, 1H), 7.53–7.49 (m, 1H), 7.38–7.32 (m, 2H), 7.28–7.21 (m, 1H), 7.17 (d, *J* = 8.3 Hz, 1H), 6.87–6.84 (m, 1H), 6.84–6.80 (m, 1H), 4.05 (t, *J* = 6.1 Hz, 2H), 3.71 (s, 2H), 3.00–2.94 (m, 2H), 2.26 (s, 3H), 1.90–1.82 (m, 2H), 1.80–1.71 (m, 2H), 1.66–1.57 (m, 2H); ^13^C NMR (151 MHz, CD_3_OD) δ 176.1, 168.6, 160.0, 143.7, 137.8, 137.7, 135.6, 135.0, 134.1, 132.2, 131.9, 131.4, 129.6, 129.5, 128.9, 127.5, 127.2, 126.8, 117.4, 113.0, 68.5, 40.7, 39.5, 29.9, 28.4, 24.2, 20.9; HRMS (MALDI) calcd for C_27_H_30_N_2_O_4_ (M+H)^+^ 447.2278, found 447.2278.

### 3-((3′-((2-(Carboxymethyl)phenyl)carbamoyl)-2-methyl-[1,1′-biphenyl]-4-yl)oxy)propan-1-aminium 2,2,2-trifluoroacetate (42)

**37** (34 mg, 0.09 mmol) was reacted with K_2_CO_3_ (27 mg, 0.20 mmol) and *tert*-butyl (3-chloropropyl)carbamate (57 mg, 0.20 mmol) in DMF (0.50 mL) at 80 °C as described for **5c**. Purification by flash column chromatography (SiO_2_, EtOAc:*n*-heptane, 2:7) gave 28 mg (57%) of methyl 2-(2-(4′-(3-((*tert*-butoxycarbonyl)amino)propoxy)-2′-methyl-[1,1′-biphenyl]-3-carboxamido)phenyl)acetate as a dark red oil: *R*_f_ = 0.33 (EtOAc:*n*-heptane, 2:3); ^1^H NMR (400 MHz, CDCl_3_) δ 9.68 (s, 1H), 8.04 (d, *J* = 8.1 Hz, 1H), 8.00–7.93 (m, 2H), 7.60–7.46 (m, 2H), 7.41–7.33 (m, 1H), 7.27–7.08 (m, 4H), 6.86–6.76 (m, 2H), 4.78 (br s, 1H), 4.06 (t, *J* = 6.0 Hz, 2H), 3.73 (s, 3H), 3.70 (s, 2H), 3.41–3.28 (m, 2H), 2.30 (s, 3H), 2.00 (p, *J* = 6.3 Hz, 2H), 1.45 (s, 9H); ^13^C NMR (101 MHz, CDCl_3_) δ 173.6, 165.8, 158.4, 156.2, 142.4, 137.1, 137.0, 134.7, 133.9, 133.0, 131.1, 131.0, 128.68, 128.65, 128.6, 125.9, 125.5, 125.4, 125.2, 116.6, 112.0, 65.9, 52.8, 39.0, 29.2, 28.6, 20.9; ESI-MS *m*/*z* 533.3 (M+H^+^).

Methyl 2-(2-(4′-(3-((*tert*-butoxycarbonyl)amino)propoxy)-2′-methyl-[1,1′-biphenyl]-3-carboxamido)phenyl)acetate (27 mg, 0.05 mmol) was hydrolyzed using aqueous 0.6 M LiOH (0.25 mL, 0.15 mmol) as described for **7** to give 26 mg (quant.) of 2-(2-(4′-(3-((*tert*-butoxycarbonyl)amino)propoxy)-2′-methyl-[1,1′-biphenyl]-3-carboxamido)phenyl)acetic acid as a pale yellow oil (*t*_R_ = 6.84 min, purity 95.8% by HPLC, method B); ^1^H NMR (400 MHz, CD_3_OD) δ 7.97–7.88 (m, 2H), 7.65–7.47 (m, 3H), 7.38–7.30 (m, 2H), 7.28–7.13 (m, 2H), 6.89–6.78 (m, 2H), 4.04 (t, *J* = 6.2 Hz, 2H), 3.71 (s, 2H), 3.25 (t, *J* = 6.8 Hz, 2H), 2.26 (s, 3H), 1.95 (p, *J* = 6.5 Hz, 2H), 1.44 (s, 9H); ^13^C NMR (101 MHz, CD_3_OD) δ 175.8, 159.9, 143.7, 137.8, 137.7, 135.5, 134.9, 134.1, 132.2, 131.8, 131.2, 129.5, 128.9, 127.5, 127.3, 126.8, 117.5, 113.1, 80.0, 66.6, 39.2, 38.5, 30.8, 28.8, 20.9; HRMS (MALDI) calcd for C_30_H_34_N_2_O_6_ (M+Na)^+^ 541.2309, found 541.2320.

2-(2-(4′-(3-((*tert*-Butoxycarbonyl)amino)propoxy)-2′-methyl-[1,1′-biphenyl]-3-carboxamido)phenyl)acetic acid (11 mg, 0.02 mmol) was Boc-deprotected using 4 M HCl in 1,4-dioxane (50 μL, 0.02 mmol) as described for **9a** and purified by preparative HPLC (0 to 100% mobile phase B (MeCN–H_2_O–TFA 90:10:0.1) in mobile phase A (H_2_O–TFA 100:0.1) over 15 min, flow rate 20 mL/min). The corresponding fractions were combined, concentrated *in vacuo*, and lyophilized to give 7 mg (71%) of **42** as a white solid of the TFA salt (*t*_R_ = 7.84 min, purity >99% by HPLC, method A); ^1^H NMR (600 MHz, CD_3_OD) δ 7.95 (d, *J* = 7.7 Hz, 1H), 7.90 (s, 1H), 7.63–7.49 (m, 3H), 7.39–7.32 (m, 2H), 7.27–7.23 (m, 1H), 7.20 (d, *J* = 8.4 Hz, 1H), 6.92–6.90 (m, 1H), 6.88–6.85 (m, 1H), 4.16 (t, *J* = 5.8 Hz, 2H), 3.72 (s, 2H), 3.18 (t, *J* = 7.3 Hz, 2H), 2.27 (s, 3H), 2.20–2.13 (m, 2H); ^13^C NMR (151 MHz, CD_3_OD) δ 175.7, 168.7, 159.5, 143.6, 138.0, 137.6, 135.6, 135.5, 134.0, 132.2, 131.9, 131.3, 129.6, 129.6, 129.0, 127.6, 127.4, 126.8, 117.4, 113.0, 66.2, 39.1, 38.7, 28.4, 20.9; HRMS (MALDI) calcd for C_25_H_26_N_2_O_4_ (M+H)^+^ 419.1965, found 419.1965.

### 4-((3′-((2-(Carboxymethyl)phenyl)carbamoyl)-[1,1′-biphenyl]-4-yl)methyl)-1-methylpiperazin-1-ium 2,2,2-trifluoroacetate (1)

1-(4-Bromobenzyl)-4-methylpiperazine: 1-Bromo-4-(bromomethyl)benzene (150 mg, 0.60 mmol) was dissolved in DCM (0.70 mL), and 1-methylpiperazine (0.13 mL, 1.17 mmol) was added dropwise. The mixture was stirred at rt for 2.5 h under an argon atmosphere. Additional DCM was added (0.5 mL), and the mixture was stirred for an additional 1 h. The reaction mixture was diluted with water and extracted with DCM (×3). The organic phases were combined, dried over Na_2_SO_4_, filtered, and concentrated *in vacuo* to give 93 mg (58%) of the product as a yellow oil: *R*_f_ = 0.80 (10% MeOH in DCM); ^1^H NMR (400 MHz, CDCl_3_) δ 7.44–7.36 (m, 2H), 7.21–7.14 (m, 1H), 3.44–3.38 (m, 2H), 2.42 (s, 8H), 2.26 (d, *J* = 1.9 Hz, 3H); ^13^C NMR (10 Hz, CDCl_3_) δ 137.5, 131.4, 130.8, 120.9, 62.3, 55.2, 53.1, 46.1; ESI-MS *m*/*z* 269.2 (M+H^+^). Spectra in accordance with the reported data.^47^

**23** (154 mg, 0.93 mmol) was coupled to **27** (172 mg, 1.10 mmol) as described for **28**, and purification by flash column chromatography (SiO_2_, 0–15% EtOAc in *n*-heptane) gave 230 mg (81%) of **31** as a yellow solid: *R*_f_ = 0.44 (EtOAc:*n*-heptane, 1:2); ^1^H NMR (400 MHz, CDCl_3_) δ 9.73 (s, 1H), 8.06–8.03 (m, 1H), 8.01 (d, *J* = 8.1 Hz, 1H), 7.93–7.88 (m, 1H), 7.56–7.49 (m, 1H), 7.45 (t, *J* = 7.9 Hz, 1H), 7.40–7.34 (m, 1H), 7.27–7.23 (m, 1H), 7.16 (td, *J* = 7.5, 1.3 Hz, 1H), 3.77 (s, 3H), 3.69 (s, 2H); ^13^C NMR (101 MHz, CDCl_3_) δ 173.7, 164.3, 136.8, 136.5, 135.1, 132.0, 131.0, 130.2, 128.7, 128.1, 125.8, 125.7, 125.3, 125.0, 53.0, 39.1.

A Schlenk flask was charged with XPhos (3 mg, 4 mol%), XPhos-Pd-G2 (2.5 mg, 2 mol%), BBA (43 mg, 0.48 mmol), and KOAc (47 mg, 0.47 mmol) under an argon atmosphere. The flask was evacuated and backfilled with argon (×4). **31** (48 mg, 0.16 mmol) was dissolved in degassed ethanol (0.50 mL) and added to the mixture. Additional degassed ethanol (1 mL) was added to the flask. The mixture was evacuated and backfilled with argon (×4), then heated to 80 °C and stirred for 1 h until the color of the reaction mixture changed from colorless to yellow. Then degassed aqueous 1.8 M K_2_CO_3_ (0.25 mL, 0.45 mmol) was added to the mixture. 1-(4-bromobenzyl)-4-methylpiperazine (58 mg, 0.22 mmol) was dissolved in degassed ethanol (0.50 mL) and added to the reaction mixture. The flask was evacuated, backfilled with argon (×3), and heated to 80 °C for 16 h. The mixture was cooled to rt, diluted with water, extracted with EtOAc, and washed with brine. The organic phases were combined, dried over Na_2_SO_4_, filtered, and concentrated *in vacuo*. The residue was purified by flash column chromatography (SiO_2_, 10% MeOH in DCM, then 10% MeOH containing 0.1% NH_3_ aq. sol. in DCM) to give 18 mg (24%) of a transesterified product ethyl 2-(2-(4′-((4-methylpiperazin-1-yl)methyl)-[1,1′-biphenyl]-3-carboxamido)phenyl)acetate as a yellow oil: *R*_f_ = 0.35 (10% MeOH in DCM); ^1^H NMR (400 MHz, CDCl_3_) δ 9.85 (s, 1H), 8.33–8.27 (m, 1H), 8.08 (d, *J* = 8.1 Hz, 1H), 7.99 (d, *J* = 7.7 Hz, 1H), 7.82–7.75 (m, 1H), 7.64 (d, *J* = 8.0 Hz, 2H), 7.57 (t, *J* = 7.7 Hz, 1H), 7.45–7.34 (m, 3H), 7.28–7.25 (m, 1H), 7.19–7.11 (m, 1H), 4.21 (q, *J* = 7.1 Hz, 2H), 3.70 (s, 2H), 3.56 (s, 2H), 2.76–2.35 (m, 8H), 2.30 (s, 3H), 1.28 (t, *J* = 7.2 Hz, 3H); ^13^C NMR (10 Hz, CDCl_3_) δ 173.3, 165.7, 141.6, 139.1, 138.1, 137.2, 135.3, 131.0, 130.4, 129.8, 129.3, 128.6, 127.2, 126.3, 126.0, 125.9, 125.4, 125.0, 62.8, 62.0, 55.3, 53.3, 46.2, 39.4, 14.2; ESI-MS *m*/*z* 472.6 (M+H^+^).

Ethyl 2-(2-(4′-((4-methylpiperazin-1-yl)methyl)-[1,1′-biphenyl]-3-carboxamido)phenyl)acetate (17 mg, 0.04 mmol) was hydrolyzed using aqueous 0.6 M LiOH (0.20 mL, 0.12 mmol) as described for **7** and purified by preparative HPLC (0 to 100% mobile phase B (MeCN–H_2_O–TFA 90:10:0.1) in mobile phase A (H_2_O–TFA 100:0.1) over 10 min, flow rate 20 mL/min). The corresponding fractions were combined and concentrated *in vacuo* (*t*_R_ = 6.81 min, purity 98.4% by HPLC, method A) to give 15 mg (75%) of **1** as a white solid of a mono-TFA salt: ^1^H NMR (400 MHz, CD_3_OD) δ 8.28–8.23 (m, 1H), 8.01–7.95 (m, 1H), 7.91–7.84 (m, 1H), 7.81–7.75 (m, 2H), 7.66–7.53 (m, 4H), 7.42–7.32 (m, 2H), 7.31–7.22 (m, 1H), 4.14 (s, 2H), 3.75 (s, 2H), 3.50–3.43 (m, 4H), 3.27–3.23 (m, 4H), 2.92 (s, 3H); ^13^C NMR (101 MHz, CD_3_OD) δ 175.7, 168.6, 142.4, 142.0, 137.6, 136.4, 133.0, 132.3, 132.2, 131.52, 131.48, 130.5, 129.0, 128.7, 127.8, 127.7, 127.5, 127.3, 61.5, 53.0, 50.1, 43.5, 39.1; HRMS (MALDI) calcd for C_27_H_29_N_3_O_3_ (M+H)^+^ 444.2281, found 444.2279. Spectra were in accordance with the reported data.^27^

### 1-(2-((*tert*-Butoxycarbonyl)amino)ethyl)-4-((3′-((2-(carboxymethyl)phenyl)carbamoyl)-[1,1′-biphenyl]-4-yl)methyl)piperazin-1-ium 2,2,2-trifluoroacetate (43)

*tert-*Butyl (2-(4-(4-chlorobenzyl)piperazin-1-yl)ethyl)carbamate: 1-(4-Chlorobenzyl)piperazine (153 mg, 0.73 mmol) was reacted with K_2_CO_3_ (146 mg, 1.06 mmol), KI (124 mg, 0.75 mmol), and *tert*-butyl (2-chloroethyl)carbamate (151 mg, 0.84 mmol) in DMF (2.1 mL) at 80 °C as described for **5c**. Purification by flash column chromatography (SiO_2_, 0–50% EtOAc in *n*-heptane) gave 84 mg (34%) of the product as a white solid: *R*_*f*_ = 0.14 (EtOAc); ^1^H NMR (400 MHz, CDCl_3_) δ 7.31–7.23 (m, 4H), 3.49 (s, 2H), 3.26 (br s, 2H), 2.52 (br s, 10H), 1.44 (s, 9H); ESI-MS *m*/*z* 354.2 (M+H^+^).

A Schlenk flask was charged with **28** (57 mg, 0.16 mmol), XPhos-Pd-G2 (3 mg, 2 mol%), XPhos (4 mg, 5 mol%), BBA (44 mg, 0.49 mmol), and KOAc (48 mg, 0.49 mmol). The flask was evacuated and backfilled with argon (×3). Then, degassed EtOH was added (1.6 mL). The flask was evacuated and backfilled with argon again. The reaction mixture was stirred at 80 °C for 1.5 h until the solution turned brown. *tert-*Butyl (2-(4-(4-chlorobenzyl)piperazin-1-yl)ethyl)carbamate (74 mg, 0.21 mmol) was dissolved in THF (0.55 mL) and added to the reaction mixture, followed by aqueous 1.8 M K_2_CO_3_ (0.26 mL, 0.47 mmol). The reaction mixture was stirred at 80 °C for 17 h. After completion, the mixture was cooled until room temperature, diluted with water, extracted with EtOAc (×3), EtOAc (1% MeOH) (×5). The organic phases were combined, washed with brine, dried over MgSO_4_, filtered, and concentrated *in vacuo*. The residue was concentrated *in vacuo* and purified on preparative HPLC (0 to 100% mobile phase B (MeCN–H_2_O–TFA 90:10:0.1) in mobile phase A (H_2_O–TFA 100:0.1) over 25 min, flow rate 20 mL/min). HPLC fractions were combined and lyophilized to give 33 mg (36%) of **43** as an off-white solid of the TFA salt (*t*_R_ = 7.53 min, purity 98.5% by HPLC, method A); ^1^H NMR (600 MHz, CD_3_OD) δ 8.36–8.33 (m, 1H), 8.05 (d, *J* = 7.7 Hz, 1H), 7.91 (d, *J* = 8.0 Hz, 1H), 7.85–7.80 (m, 1H), 7.71–7.67 (m, 2H), 7.58 (t, *J* = 7.7 Hz, 1H), 7.46–7.42 (m, 2H), 7.32–7.24 (m, 2H), 7.15–7.10 (m, 1H), 3.67 (s, 2H), 3.60 (s, 2H), 3.23–3.18 (m, 2H), 2.79–2.50 (m, 11H), 1.42 (s, 9H); ^13^C NMR (151 MHz, CD_3_OD) δ 180.3, 167.9, 163.1 (q, *J* = 34.9 Hz), 158.4, 142.5, 141.0, 138.1, 136.8, 136.6, 131.8, 131.7, 131.5, 131.4, 130.4, 128.2, 128.0, 127.6, 127.2, 126.3, 125.5, 118.2 (q, *J* = 292.9 Hz), 80.2, 62.9, 58.4, 53.4, 53.0, 43.9, 38.0, 28.7; HRMS (MALDI) calcd for C_33_H_40_N_4_O_5_ (M+H)^+^ 573.3071, found 573.3068.

### 4-((3′-((2-(Carboxymethyl)phenyl)carbamoyl)-2-methyl-[1,1′-biphenyl]-4-yl)methyl)-1-methylpiperazin-1-ium 2,2,2-trifluoroacetate (44)

1-(4-Bromo-3-methylbenzyl)-4-methylpiperazine: A dry round-bottom flask was charged with DCM (1.4 mL), 4-bromo-3-methylbenzaldehyde (67 μL, 0.50 mmol), and 1-methylpiperazine (56 μL, 0.50 mmol) under an argon atmosphere. The reaction mixture was stirred for 10 min before sodium triacetoxyborohydride (148 mg, 0.70 mmol) was added, and the mixture was stirred for 17 h. After completion, sat. aq. NaHCO_3_ (6 mL) was added, and the mixture was stirred for 15 min. The reaction mixture was extracted with EtOAc (×3) and washed with brine. The organic phases were combined, dried over MgSO_4_, filtered, and concentrated *in vacuo* to give 117 mg (83%) of the product as a pale yellow oil: *R*_f_ = 0.11 (DCM:MeOH, 20:1); ^1^H NMR (400 MHz, CDCl_3_) δ 7.45 (d, *J* = 8.1 Hz, 1H), 7.18 (d, *J* = 2.2 Hz, 1H), 7.03–6.96 (m, 1H), 3.43 (s, 2H), 2.52 (s, 8H), 2.38 (s, 3H), 2.34 (br s, 3H); ^13^C NMR (101 MHz, CDCl_3_) δ 137.8, 137.4, 132.3, 131.7, 128.3, 123.5, 62.3, 55.1, 52.7, 45.8, 23.0; ESI-MS *m*/*z* 283.1 (M+H^+^).

A Schlenk flask was charged with **28** (15 mg, 0.04 mmol), XPhos-Pd-G2 (1.4 mg, 4 mol%), XPhos (1.6 mg, 8 mol%), BBA (13 mg, 0.15 mmol), and KOAc (14 mg, 0.14 mmol). The flask was evacuated and backfilled with argon (×3). Then, degassed EtOH was added (0.43 mL). The flask was evacuated and backfilled with argon again. The reaction mixture was stirred at 80 °C for 2.5 h until the solution turned brownish. 1-(4-Bromo-3-methylbenzyl)-4-methylpiperazine (21 mg, 0.07 mmol) was dissolved in THF (0.50 mL) and added to the reaction mixture followed by aqueous 1.8 M K_2_CO_3_ (0.10 mL, 0.18 mmol). The reaction mixture was stirred at 80 °C for 22 h. After completion, the mixture was cooled to rt, concentrated *in vacuo*, added 0.1% TFA in Milli-Q water, and filtered. The residue was purified on preparative HPLC (0 to 100% mobile phase B (MeCN–H_2_O–TFA 90:10:0.1) in mobile phase A (H_2_O–TFA 100:0.1) over 25 min, flow 20 mL/min). HPLC fractions were combined and lyophilized to give 15 mg (61%) of **44** as a white sticky solid as a hydrolyzed product (*t*_R_ = 6.87 min, purity 98.7% by HPLC, method A) as a mono-TFA salt; ^1^H NMR (600 MHz, CD_3_OD) δ 8.02–7.97 (m, 1H), 7.94–7.90 (m, 1H), 7.63–7.58 (m, 2H), 7.57–7.52 (m, 1H), 7.42–7.30 (m, 5H), 7.26 (td, *J* = 7.5, 1.3 Hz, 1H), 4.08 (s, 2H), 3.72 (s, 2H), 3.45 (br s, 4H), 3.23 (br s, 4H), 2.92 (s, 3H), 2.31 (s, 3H); ^13^C NMR (151 MHz, CD_3_OD) δ 175.7, 168.5, 161.8 (q, *J* = 37.1 Hz), 143.2, 143.1, 137.6, 137.5, 135.8, 133.7, 133.5, 133.0, 132.2, 131.43, 131.39, 129.8, 129.4, 129.03, 129.02, 127.7, 127.5, 127.4, 117.5 (q, *J* = 290.7 Hz), 61.7, 53.1, 50.2, 43.5, 39.1, 20.6; HRMS (MALDI) calcd for C_28_H_31_N_3_O_3_ (M+H)^+^ 458.2438, found 458.2435.

### 1-(2-((*tert-*Butoxycarbonyl)amino)ethyl)-4-((3′-((2-(carboxymethyl)phenyl)carbamoyl)-2-methyl-[1,1′-biphenyl]-4-yl)methyl)piperazin-1-ium 2,2,2-trifluoroacetate (45)

*tert*-Butyl 4-(4-bromo-3-methylbenzyl)piperazine-1-carboxylate: *tert*-Butyl piperazine-1-carboxylate (140 mg, 0.75 mmol) was reacted with 4-bromo-3-methylbenzaldehyde (101 μL, 0.76 mmol) as described for 1-(4-bromo-3-methylbenzyl)-4-methylpiperazine to give 272 mg (98%) of the product as a colorless oil: *R*_f_ = 0.46 (EtOAc:*n*-heptane, 1:1); ^1^H NMR (400 MHz, CDCl_3_) δ 7.47 (d, *J* = 8.1 Hz, 1H), 7.23 (s, 1H), 7.04 (d, *J* = 8.2 Hz, 1H), 3.48 (s, 6H), 2.39 (s, 7H), 1.45 (s, 9H); ^13^C NMR (101 MHz, CDCl_3_) δ 154.8, 132.4, 131.9, 128.4, 79.9, 62.3, 52.9, 28.6, 23.0; ESI-MS *m*/*z* 369.1 (M+H^+^).

4-(4-Bromo-3-methylbenzyl)piperazin-1-ium chloride: *tert*-Butyl 4-(4-bromo-3-methylbenzyl)piperazine-1-carboxylate (258 mg, 0.70 mmol) was Boc-deprotected using 4 M HCl in 1,4-dioxane (1.3 mL, 5.20 mmol) as described for **9a** to give 214 mg (quant.) of the product as a white solid that was used directly in the next step; ESI-MS *m*/*z* 269.1 (M+H+).

4-(4-Bromo-3-methylbenzyl)piperazin-1-ium chloride (125 mg, 0.41 mmol) was reacted with K_2_CO_3_ (142 mg, 1.02 mmol), KI (68 mg, 0.41 mmol) and *tert*-butyl (2-chloroethyl)carbamate (92 mg, 0.51 mmol) in DMF (1.2 mL) at 80 °C as described for **5c**. Purification by flash column chromatography (SiO_2_, EtOAc:*n*-heptane, 1:1 → 1:2 → 100% EtOAc) to give 96 mg (57%) of *tert*-butyl (2-(4-(4-bromo-3-methylbenzyl)piperazin-1-yl)ethyl)carbamate as a yellow oil: *R*_*f*_ = 0.15 (EtOAc); ^1^H NMR (400 MHz, CDCl_3_) δ 7.50–7.43 (m, 1H), 7.19 (s, 1H), 7.01 (d, *J* = 8.1 Hz, 1H), 5.14 (br s, 1H), 3.49–3.44 (m, 2H), 3.29 (br s, 2H), 2.58 (br s, 10H), 2.38 (s, 3H), 1.44 (s, 9H); ^13^C NMR (101 MHz, CDCl_3_) δ 156.1, 138.0, 132.4, 131.8, 128.4, 62.2, 57.4, 52.9, 28.6, 23.0; ESI-MS *m*/*z* 412.2 (M+H^+^).

**28** (39 mg, 0.11 mmol) was reacted with, XPhos-Pd-G2 (2 mg, 2 mol%), XPhos (2 mg, 4 mol%), BBA (30 mg, 0.33 mmol), and KOAc (32 mg, 0.33 mmol). The flask was evacuated and backfilled with argon (×3). Then, degassed EtOH was added (0.53 mL). The flask was evacuated and backfilled with argon again. The reaction mixture was stirred at 80 °C for 2 h until the solution turned brownish. *tert*-Butyl (2-(4-(4-bromo-3-methylbenzyl)piperazin-1-yl)ethyl)carbamate (59 mg, 0.14 mmol) was dissolved in THF (0.50 mL) and added to the reaction mixture followed by aqueous 1.8 M K_2_CO_3_ (0.18 mL, 0.33 mmol). The reaction mixture was stirred at 80 °C for 20 h. After completion, the mixture was cooled to rt, filtered through a pad of Celite, neutralized with 0.1% TFA in Milli-Q water, concentrated *in vacuo*, and filtered. The residue was purified on preparative HPLC (0 to 100% mobile phase B (MeCN–H_2_O–TFA 90:10:0.1) in mobile phase A (H_2_O–TFA 100:0.1) over 25 min, flow 20 mL/min). HPLC fractions were combined and lyophilized to give 58 mg (75%) of **45** as a colorless oil (*t*_R_ = 7.70 min, purity 96.9% by HPLC, method A) as a mono-TFA salt; ^1^H NMR (600 MHz, CD_3_OD) δ 8.02–7.97 (m, 1H), 7.95–7.91 (m, 1H), 7.64–7.58 (m, 2H), 7.57–7.52 (m, 1H), 7.40 (s, 1H), 7.38–7.31 (m, 4H), 7.28–7.23 (m, 1H), 4.08 (s, 2H), 3.72 (s, 2H), 3.35–3.32 (m, 2H), 3.16 (br s, 8H), 2.98–2.93 (m, 2H), 2.32 (s, 3H), 1.44 (s, 9H); ^13^C NMR (151 MHz, CD_3_OD) δ 175.7, 168.5, 158.7, 143.3, 143.1, 137.6, 137.5, 135.8, 133.7, 133.6, 132.7, 132.2, 131.41, 131.37, 129.8, 129.4, 129.1, 129.0, 127.7, 127.40, 127.38, 80.7, 61.6, 57.8, 51.8, 51.5, 39.1, 37.2, 28.7, 20.6; HRMS (MALDI) calcd for C_34_H_42_N_4_O_5_ (M+H)^+^ 587.3228, found 587.3232.

### 4-((3′-((2-(Carboxymethyl)phenyl)carbamoyl)-2-methyl-[1,1′-biphenyl]-4-yl)methyl)-1-(2-((7-nitrobenzo[*c*][1,2,5]oxadiazol-4-yl)amino)ethyl)piperazin-1-ium 2,2,2-trifluoroacetate (46)

2-(4-((3′-((2-(Carboxymethyl)phenyl)carbamoyl)-2-methyl-[1,1′-biphenyl]-4-yl)methyl)piperazin-1-yl)ethan-1-aminium 2,2,2-trifluoroacetate: **45** (58 mg, 0.08 mmol) was dissolved in DCM (1.2 mL). Then, TFA (60 μL, 0.78 mmol) was added dropwise. The reaction mixture was stirred at rt for 3 h. After completion, the mixture was co-evaporated with DCM (×5), Milli-Q water (1 mL) was added, and the mixture was lyophilized to give 64 mg (quant.) of the product as a white solid that was used directly in the next step; ESI-MS *m*/*z* 487.3 (M+H^+^).

A dry flask was charged with 2-(4-((3′-((2-(carboxymethyl)phenyl)carbamoyl)-2-methyl-[1,1′-biphenyl]-4-yl)methyl)piperazin-1-yl)ethan-1-aminium 2,2,2-trifluoroacetate (24 mg, 0.03 mmol), MeOH (0.80 mL), NEt_3_ (28 μL, 0.20 mmol), and NBD-Cl (8 mg, 0.04 mmol) under an argon atmosphere. The reaction mixture was stirred at rt in the dark for 29 h. After completion, the reaction mixture was cooled to rt, concentrated *in vacuo*. The residue was purified by preparative HPLC (0–100% mobile phase B (MeCN–H_2_O–TFA 90:10:0.1) in mobile phase A (H_2_O–TFA 100:0.1) over 10 min, flow rate 20 mL/min). The corresponding fractions were combined, concentrated *in vacuo*, and lyophilized to give 8 mg (30%) of **46** as an orange solid (*t*_R_ = 8.33 min, purity >99% (254 nm) and 98.8% (450 nm) by HPLC, method A); ^1^H NMR (400 MHz, CD_3_CN) δ 9.35 (s, 1H), 8.50 (d, *J* = 8.1 Hz, 1H), 7.98 (d, *J* = 7.5 Hz, 1H), 7.88 (s, 1H), 7.79–7.52 (m, 4H), 7.46–7.30 (m, 5H), 7.26–7.18 (m, 1H), 6.35 (d, *J* = 8.5 Hz, 1H), 4.17 (s, 2H), 3.87–3.65 (m, 4H), 3.38–3.03 (m, 10H), 2.28 (s, 3H); HRMS (MALDI) calcd for C_35_H_35_N_7_O_6_ (M+H)^+^ 650.2721, found 650.2727.

### cAMP Assays

cAMP assays were conducted using Flp-In T-REx 293 cells engineered to express human or mouse SUCNR1 in an inducible fashion in response to treatment with the antibiotic, doxycycline (Dox). Cells were treated with 100 ng/mL Dox and cultured overnight prior to the assay. Cells were non-enzymatically detached from the culture dish using Versene, before washing and resuspending in Hank’s Balanced Salt Solution (HBSS). Cells were then seeded in low volume 384-well plates at 2000 cells per well in HBSS containing 3-isobutyl-1-methylxanthine. Cells were treated with both test compounds and 1 μM forskolin for 30 min at 37 °C. After the 30 min treatment, cAMP was measured using a homogeneous time-resolved FRET cAMP kit (PerkinElmer) according to the manufacturer instructions with a PheraStar FS microplate reader (BMG Labtech).

### BRET Binding Assays

BRET binding assays were carried out using Flp-In T-REx 293 cells engineered to inducibly express human or mouse SUCNR1 tagged at their N terminal with Nluc. To help ensure proper membrane expression of the Nluc-SUCNR1 constructs, the signal peptide sequence for the mGlu5 glutamate receptor was added to the construct immediately before the Nluc sequence. For binding assays, cells were plated in poly-d-lysine-coated 96-well plates (white or black with clear bottom), and Nluc-SUCNR1 expression was induced with overnight Dox treatment (100 ng/mL). Prior to the assays, culture medium was removed, and cells were washed twice with HBSS. Cells were then incubated in HBSS at 37 °C for 30 min. The NanoGlo Nluc substrate (Promega, N1110) was then added to the cells, at a final 1:800 dilution, and incubated for 10 min. For equilibrium assays (saturation or competition), the indicated fluorescent or non-fluorescent compounds were added, and plates were incubated for a further 5 min at 37 °C before reading. Luminescent emissions at 545 and 460 nm were measured using a ClarioStar microplate reader (BMG Labtech). For kinetic assays, luminescent emissions at 535 and 475 nm were measured using a PheraStar plate reader (BMG Labtech) at the indicated time intervals. For association kinetics, baseline readings were taken before the addition of test compounds, followed by injection of the test compound by the plate reader. For dissociation kinetics, baseline measurements were taken for 2 min with cells incubated with the indicated concentration of tracer ligand, followed by injection of a 100 μM concentration of the competing ligand by the plate reader to measure dissociation of the tracer. BRET ratios were calculated as 545/460 (ClarioStar assays) or 535/475 (PheraStar assays).

### G Protein Activation BRET Assay

G protein activation assays were carried out using the open-source TRUPATH platform previously described.^36^ HEK-293T cells were transfected with Gαi1-Rluc8, Gγ8-GFP^2^, Gβ3 and either human, mouse, mouse-N18E/K269N, or mouse-N18E/G84W/K269N SUCNR1 in a 1:1:1:1 plasmid mass ratio using polyethyleneimine. After 24 h, cells were trypsinized and seeded into poly-d-lysine-coated white 96-well plates. After a further 24 h in culture, medium was removed, cells were washed twice with HBSS and incubated in HBSS at 37 °C for 30 min. Prolume purple substrate (NanoLight Technology, Cat. No. 369) was added to the cells at a final 5 μM concentration and incubated for 10 min at 37 °C. A background measurement was then taken by reading luminescent emission at 525 and 385 nm using a ClarioStar microplate reader (BMG Labtech). Succinate was added to the cells and incubated for a further 5 min at 37 °C before reading luminescence again at 525 and 385 nm again. For the antagonist assays, **1** was added to the cells and incubated for 5 min before addition of prolume purple and a total of 15 min before succinate was added. For analysis, the ratio of 525/385 emission was taken and expressed as a fold change before and after the addition of succinate.

### Computational Modeling

The X-ray structure of the humanized rSUCNR1 (PDB code 6RNK)^27^ was cleaned from excess water and other additives and prepared using Protein Preparation in Maestro (Schrödinger, LCC, version 12.7.161). Three mutations, E18N, N269K, and W84G, were introduced to make the murinized rSUCNR1 model. **9d** and **1** were prepared using ligprep (OPLS4 forcefield and standard settings) and docked in the humanized and murinized rSUCNR1 using InducedFit docking with Glide Extra Precision (XP) and default settings with the box centered around **1**.

## Supporting Information Available

Concentration–response curves for succinate and **1**, saturation binding for **7** at hmSUCNR1, spectroscopic characterization of tracers **7**, **22**, and **46**, HPLC chromatograms of **7**, **22**, and **46**, and ^1^H NMR spectra of **7**, **22**, and **46** (PDF)
Model of **1** in complex with hrSUCNR1 (PDB)
Model of **1** in complex with mrSUCNR1 (PDB)
Model of **9d** in complex with hrSUCNR1 (PDB)
Molecular formula strings for the final compounds (CSV)

## Supplementary Material

jm3c00552_si_002.pdf

jm3c00552_si_003.pdb

jm3c00552_si_004.pdb

jm3c00552_si_005.pdb

## Abbreviations Used

BBA: bis-boric acid
BRET: bioluminescence resonance energy transfer
BTFFH: fluoro-*N,N,N′,N′-*bis(tetramethylene)formamidinium hexafluorophosphate
DHB: 2,5-dihydroxybenzoic acid
DIPEA: diisopropylethylamine
DMP: Dess–Martin periodinane
Dox: doxycycline
FFA1: free fatty acid receptor 1
FFA2: free fatty acid receptor 2
HATU: 1-[bis(dimethylamino)methylene]-1*H*-1,2,3-triazolo[4,5-*b*]pyridinium 3-oxid hexafluorophosphate
HBSS: Hank’s balanced salt solution
NBD: 4-amino-7-nitrobenzoxadiazole
Nluc: nanoluciferase
RET: resonance energy transfer
SUCNR1: succinate receptor (GPR91)
XPhos-Pd-G2: chloro(2-dicyclohexylphosphino-2′,4′,6′-triisopropyl-1,1′-biphenyl)[2-(2′-amino-1,1′-biphenyl)]palladium(II)
XPhos-Pd-G4: methanesulfonato(2-dicyclohexylphosphino-2′,4′,6′-tri-isopropyl-1,1′-biphenyl)(2′-methylamino-1,1′-biphenyl-2-yl)palladium(II)
