# Supplementary material for: Development and Characterization of Potent Succinate Receptor Fluorescent Tracers
Source: J Med Chem. 2023 Jun 15;66(13):8951–74. doi: 10.1021/acs.jmedchem.3c00552 (PMC10350927; doi:10.1021/acs.jmedchem.3c00552)
Supplement: Supplementary file 1 — jm3c00552_si_002.pdf [file jm3c00552_si_002.pdf]

Supporting Information

Development and Characterization of Potent Succinate Receptor Fluorescent Tracers

Marija Ciba[†], Bethany Dibnah[§], Brian D. Hudson[§], Elisabeth Rexen Ulven[†]**

[†]Department of Drug Design and Pharmacology, University of Copenhagen, Universitetsparken 2, DK-2100 Copenhagen, Denmark

[§]Centre for Translational Pharmacology, School of Molecular Biosciences, College of Medical, Veterinary and Life Sciences, University of Glasgow, Glasgow G12 8QQ, Scotland, United Kingdom

*E-mails: Brian.Hudson@glasgow.ac.uk & eru@sund.ku.dk

TABLE OF CONTENTS

Saturation binding for **7**	S2
Saturation and dissociation binding kinetics of **22**	S3
Saturation and dissociation binding kinetics of **46**	S4
Concentration response curves for **1**	S5
Saturation binding at hmSUCNR1	S6
Spectroscopic characterization of fluorescent tracers **7**, **22**, **46**	S7
HPLC chromatograms of **7**, **22**, **46**	S10
^{1}H NMR spectra of **7**, **22**, **46**	S13

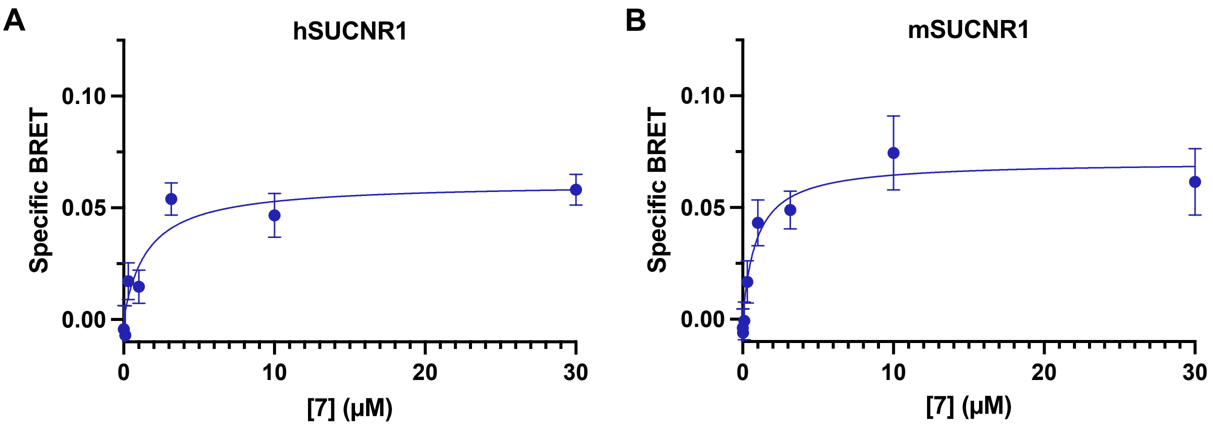

Figure S1. Saturation binding for **7** at hSUCNR1 and mSUCNR1. The saturation binding data for **7** that is shown in Figure 2 is presented as specific binding, by subtracting the non-specific BRET (obtained from cells treated with 100 μM **17**) from the total binding. Data are presented as mean ±SEM from three independent experiments. Data were fit to a one-site specific binding model.

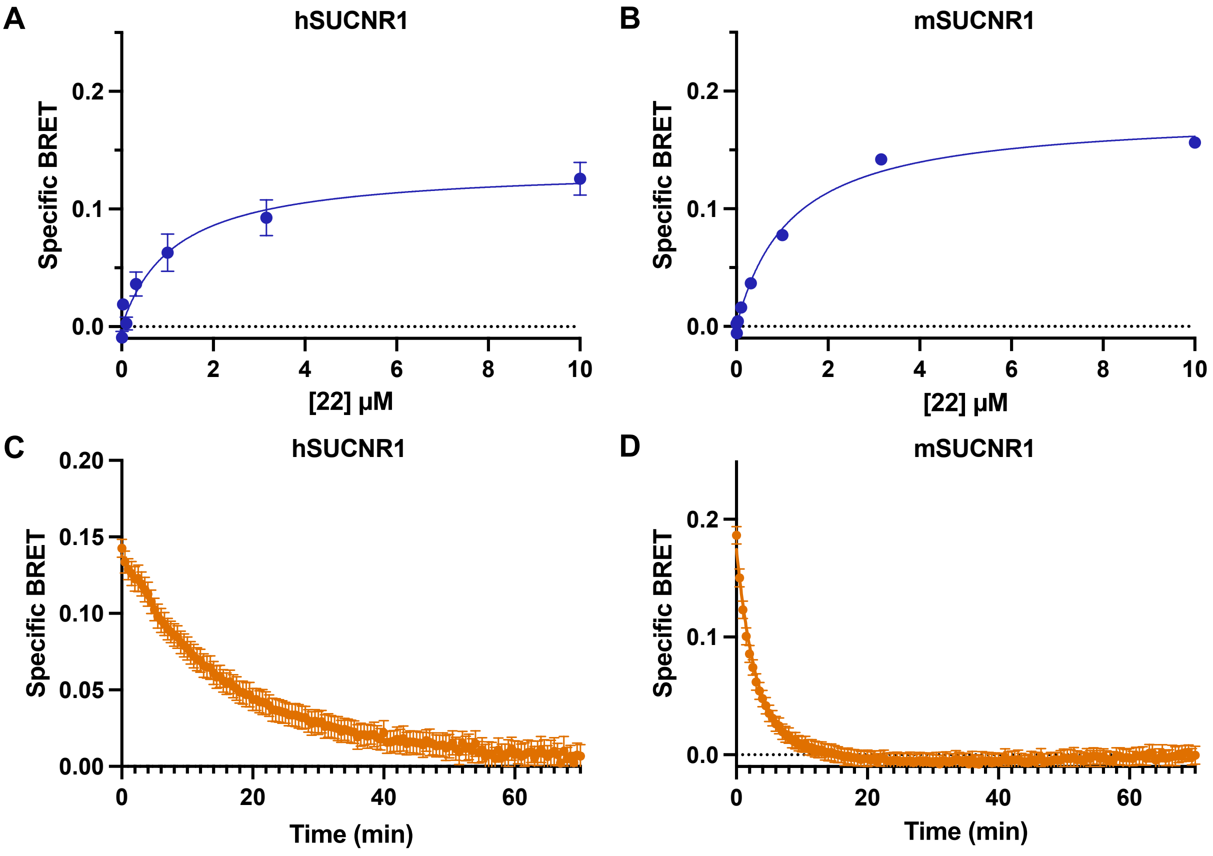

Figure S2. Saturation and dissociation binding kinetics of **22** at hSUCNR1 and mSUCNR1. The saturation binding data for **22** shown in Figure 4 are presented as specific binding at hSUCNR1 (A) and mSUCNR1 (B). Specific binding was obtained by subtracting the non-specific BRET (cells treated with 100 µM **17**) from the total binding. Saturation binding data are presented as mean ±SEM from three independent experiments. Data were fit to a one-site specific binding model. Dissociation rate experiments at hSUCNR1 (C) or mSUCNR1 (D) where cells were first incubated with **22** (3 µM), before 100 µM **17** was added at 0 min. Data are the mean ± SEM of three independent experiments and are fit to a one site exponential decay model.

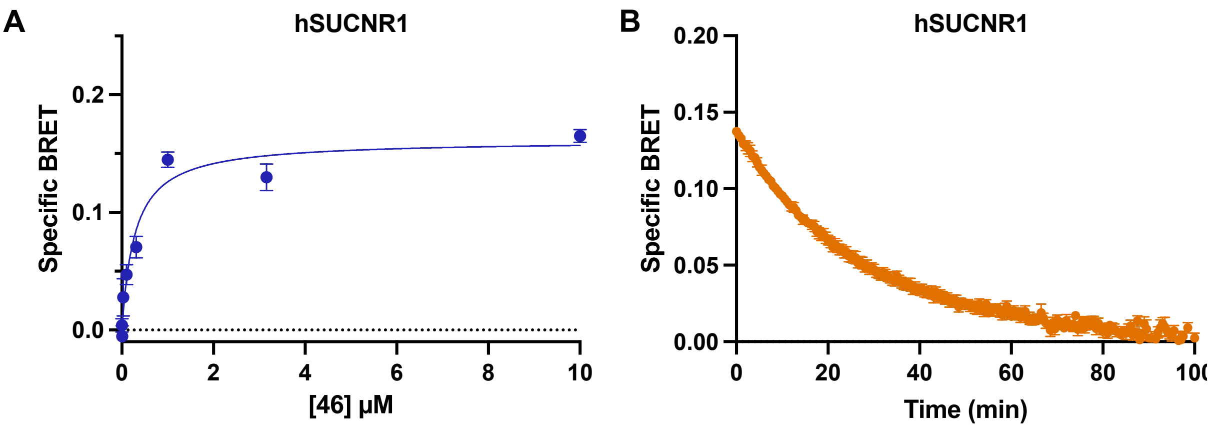

Figure S3. Saturation and dissociation binding kinetics of **46** at hSUCNR1. The saturation binding data for **46** shown in Figure 5B are presented as specific binding for human SUCNR1 (A). Specific binding was obtained by subtracting the non-specific BRET (cells treated with 100 µM **17**) from the total binding. Saturation binding data are presented as mean ±SEM from three independent experiments. Data were fit to a one-site specific binding model. In B, a dissociation rate experiment is presented for hSUCNR1 incubated with **46** (562 nM), before 100 µM **17** was added at 0 min. Data are the mean ± SEM of three independent experiments and are fit to a one site exponential decay model.

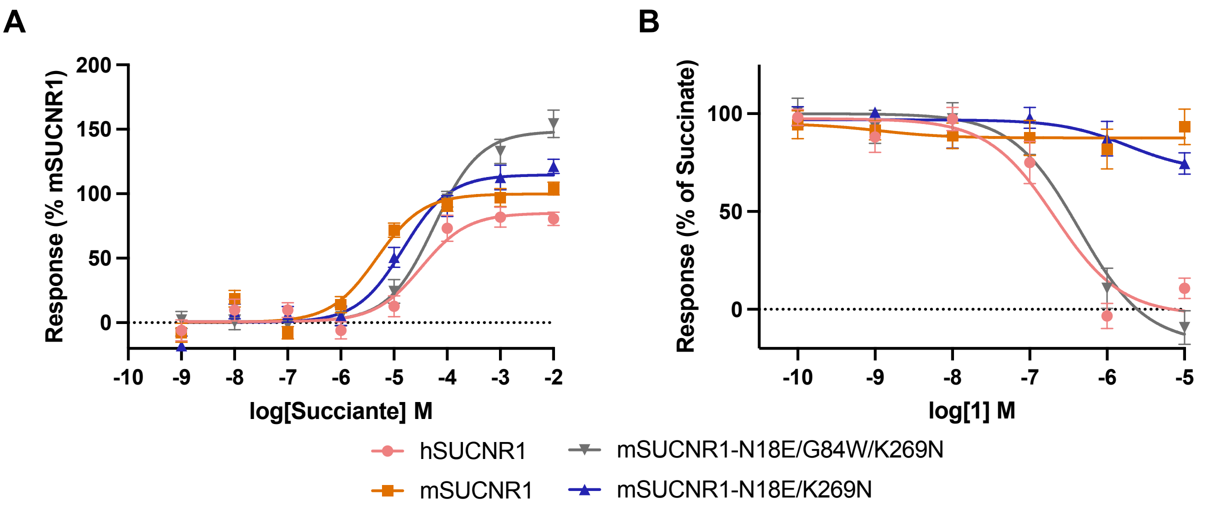

Figure S4. Humanizing N18E/G84W/K269N mutations of mSUCNR1 result in gain of antagonist function. hSUCNR1, mSUCNR1, mSUCNR1-N18E/K269N and mSUCNR1-N18E/G84W/K269N were transfected into HEK-293T cells with a BRET biosensor measuring activation of Gα_{i2}. Each SUCNR1 construct responded to succinate treatment with activation of the biosensor (A). Antagonism experiments were then conducted testing concentration responses to **1** to inhibit an EC_{80} concentration of succinate for each receptor construct: hSUCNR1 (300 μM), mSUCNR1 (10 μM), mSUCNR1-N18E/K269N (30 μM) and mSUCNR1-N18E/G84W/K269N (300 μM) (B). All data are shown as mean ± SEM from three independent experiments completed in triplicate.

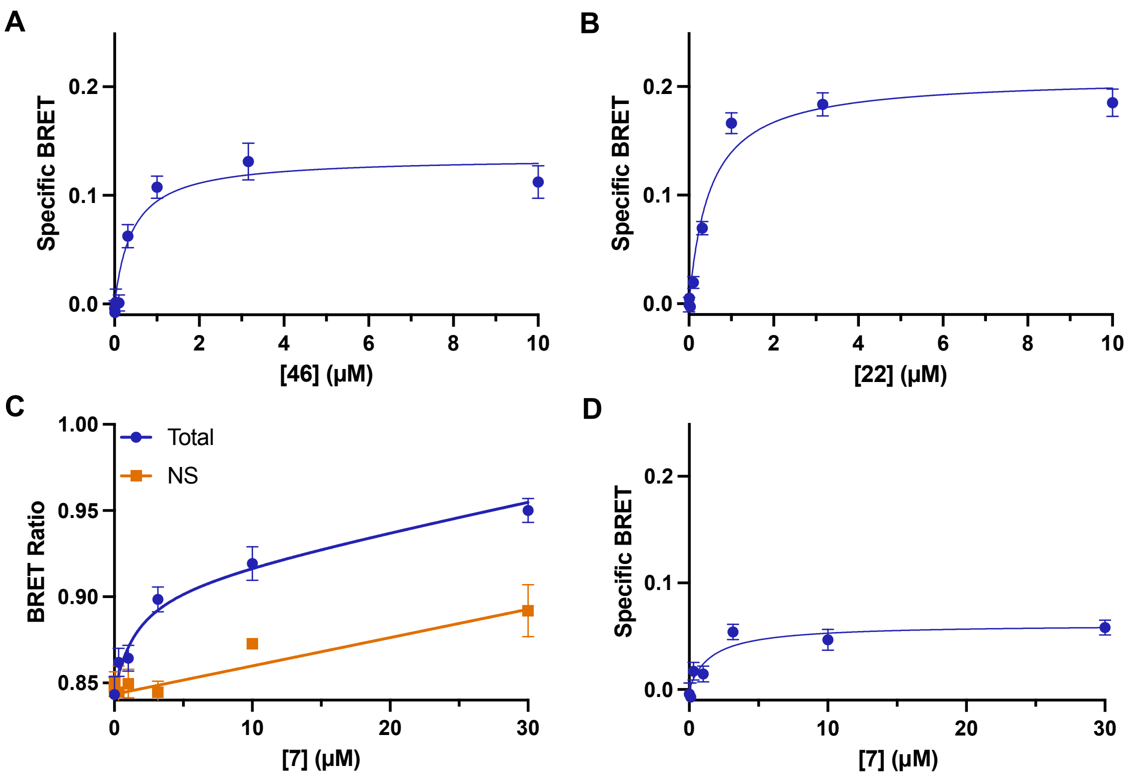

Figure S5. Saturation binding at hmSUCNR1. The saturation binding data for **46** (A) and **22** (B) shown in Figure 6C and 6D are presented as specific binding by subtracting the non-specific BRET (obtained from cells treated with 100 μM **17**) from the total binding and fitting to a one-site specific binding model. In C, saturation binding data are shown for **7** at hmSUCNR1. Non-specific binding (NS) was measured by addition of 100 μM **17**. Data were globally fit to a total and non-specific binding equation. D shows the same data as C, but as specific binding with non-specific subtracted from total binding and fit to a one-site specific binding model. All data presented are the mean ± SEM of three independent experiments.

SPECTROSCOPIC CHARACTERIZATION OF FLUORESCENT TRACERS

The absorption and emission spectra of **7**, **22**, **46** were recorded in *n*-octanol, which served as a presumed mimic of the lipophilic environment in the binding pocket, and phosphate saline buffer ($PBS_{7.4}$). All ligands exhibited acceptable Stokes shift (53-61 nm) and the molar extinction coefficients (ε) were within the expected range (ε = 8591-17166 M^{-1} cm^{-1}) compared to the previously reported NBD-based tracers for FFA1[1] and FFA2[2] (Figure S6A, Table S1). A typical bathochromic shift of NBD-amino derivates[3] in both absorption (data now shown) and emission spectra (Figure S6B) was observed for all tracers in a polar solvent, $PBS_{7.4}$, when compared to apolar *n*-octanol (Table S1). **7** showed the most red-shifted absorption spectrum (24 nm) and **22** showed only a slight 4 nm shift in $PBS_{7.4}$ compared to *n*-octanol.

Table S1. Absorption and fluorescence properties of NBD tracers.

	n-octanol					$PBS_{7.4}$	
	λ_{ex} [nm]	λ_{em} [nm]	SS[a] [nm]	ε[b] [$M^{-1}cm^{-1}$]	QY[c]	λ_{ex} [nm]	λ_{em} [nm]
**7**	467	520	53	17166	0.35	491	537
**22**	463	524	61	8519	n.d.	467	528
**46**	462	515	53	14541	0.05	478	534

[a]SS, Stokes shift; [b]ε, molar extinction coefficient at λ_{ex}; [c]QY, relative fluorescence quantum yield (fluorescein in 0.1 M NaOH (aq) used as an internal standard[4]).

In line with the previous observations for the NBD-based FFA1 and FFA2 tracers,[1,2] the emission spectra of **7**, **22**, and **46** showed almost a complete lack of fluorescence in the aqueous environment, however the lack of fluorescence was also observed for **22** in *n*-octanol (Figure S6B). Therefore, the emission spectra of **22** was additionally recorded in DMSO and EtOH, where the fluorescence intensity of **22** in DMSO was the highest (Figure S6B). **7** showed a 7-fold higher quantum yield (QY) compared to **46** in *n*-octanol, however the emission intensity

of **22** was too low to determine the QY in *n*-octanol (Table S1). Although the agonist tracer **7** demonstrated more favorable spectral characteristics, the optimized **22** outperformed **7** by showing excellent EC_{50} and K_d values in our *in vitro* assays.

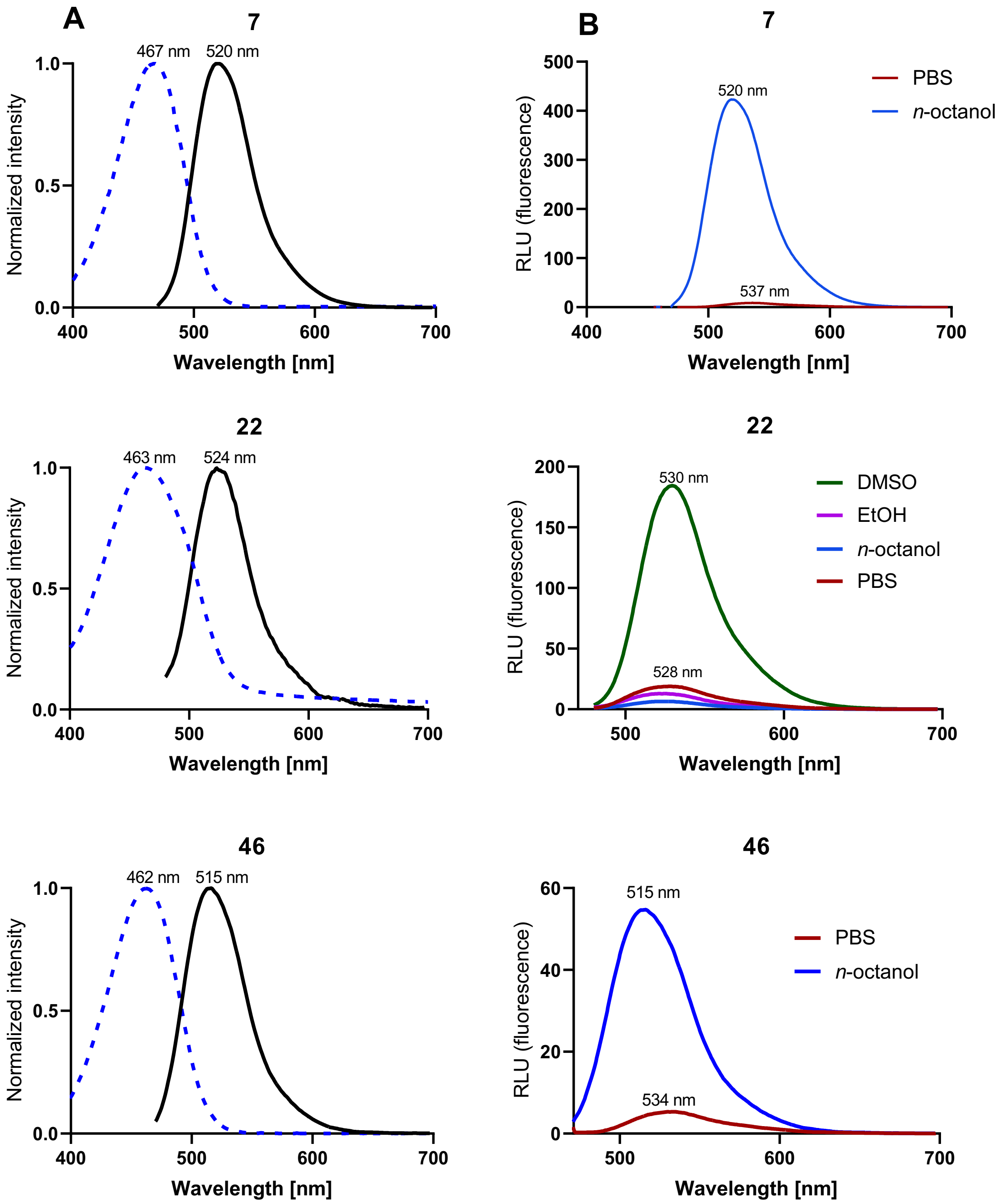

Figure S6. Absorption and emission characteristics of NBD tracers. A: Absorption and emission spectra of **7**, **22**, **46** recorded in *n*-octanol at 9.83 µM. B: Emission spectra of **7**, **22**, **46** recorded in different solvents using the same instrument settings.

Experimental details

The solutions of **7**, **22**, and **46** were prepared by diluting 10 mM DMSO stock solutions of the corresponding compounds in *n*-octanol, $PBS_{7.4}$, EtOH (for **22**). Single concentration emission spectra of all fluorescent compounds were recorded at 9.83 μM by exciting at corresponding λ_{ex} (462 nm, 463 nm, 467 nm) on PerkinElmer LS50B luminescence spectrometer in duplicate. For determination of extinction coefficients, absorption spectra at increasing concentrations of the **7**, **22**, **46** in *n*-octanol and $PBS_{7.4}$ were recorded in duplicate using a flat-bottom transparent 96-well plate (Greiner) on a microplate reader Safire 2 (Tecan) with XFLUOR4SAFIREII Version: V 4.62n software. For quantum yield determination, emission spectra at increasing concentrations of fluorescein in aqueous 0.1 M NaOH (internal standard, $\Phi = 0.93$[5]), and **7** and **46** in *n*-octanol were recorded by exciting the internal standard, **7** and **46** at a fixed wavelength of 450 nm. All experiments were performed at room temperature. Data analysis was performed using Graphpad Prism v9 and emission spectra were background-subtracted and averaged using Spectragryph version v1.2.16.1.[6]

HPLC CHROMATOGRAMS OF THE FLUORESCENT TRACERS

Compound 7 (254 and 450 nm)

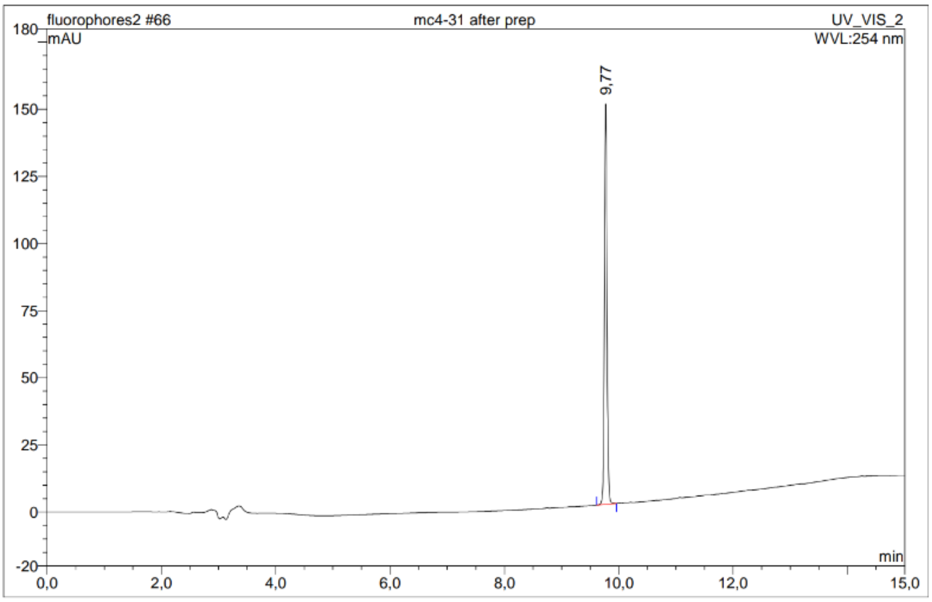

No.	Ret.Time min	Peak Name	Height mAU	Area mAU*min	Rel.Area %	Amount	Resolution(EP)
1	9,77	n.a.	149,088	7,637	100,00	n.a.	n.a.
Total:			149,088	7,637	100,00	0,000	

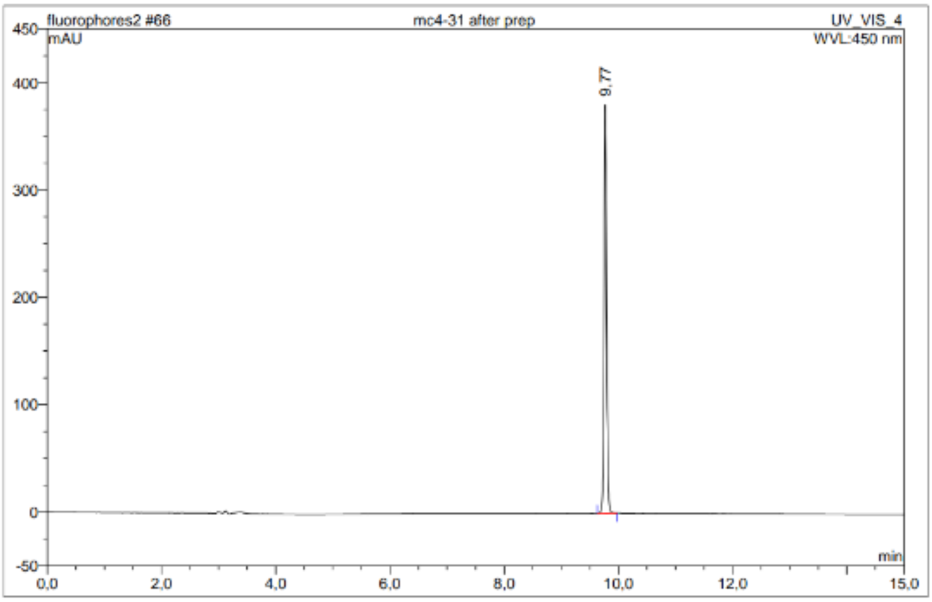

No.	Ret.Time min	Peak Name	Height mAU	Area mAU*min	Rel.Area %	Amount	Resolution(EP)
1	9,77	n.a.	380,609	19,536	100,00	n.a.	n.a.
Total:			380,609	19,536	100,00	0,000	

Compound 22 (254 and 450 nm)

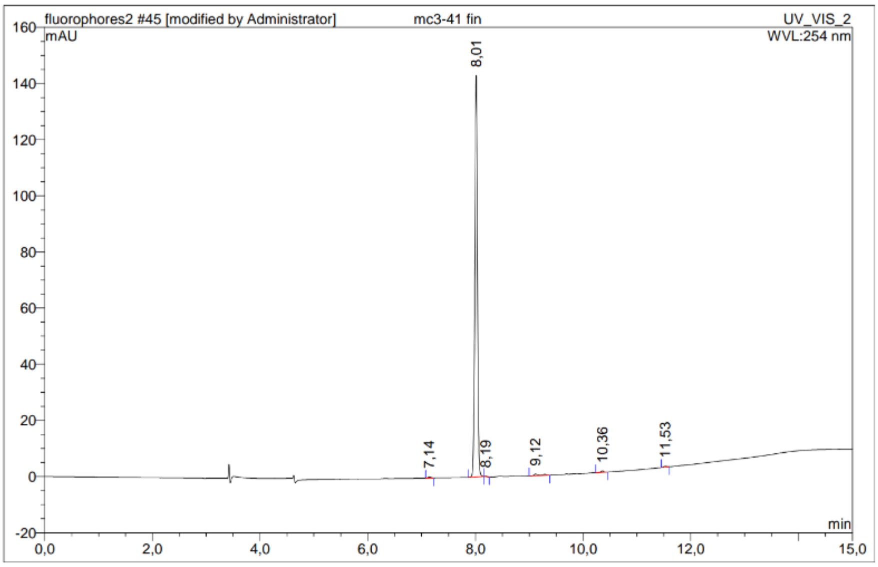

No.	Ret.Time min	Peak Name	Height mAU	Area mAU*min	Rel.Area %	Amount	Resolution(EP)
1	7,14	n.a.	0,417	0,026	0,33	n.a.	9,22
2	8,01	n.a.	142,892	7,830	98,13	n.a.	2,39
3	8,19	n.a.	0,202	0,007	0,09	n.a.	9,73
4	9,12	n.a.	0,544	0,070	0,87	n.a.	11,77
5	10,36	n.a.	0,482	0,024	0,31	n.a.	11,78
6	11,53	n.a.	0,306	0,022	0,27	n.a.	n.a.
Total:			144,843	7,979	100,00	0,000	

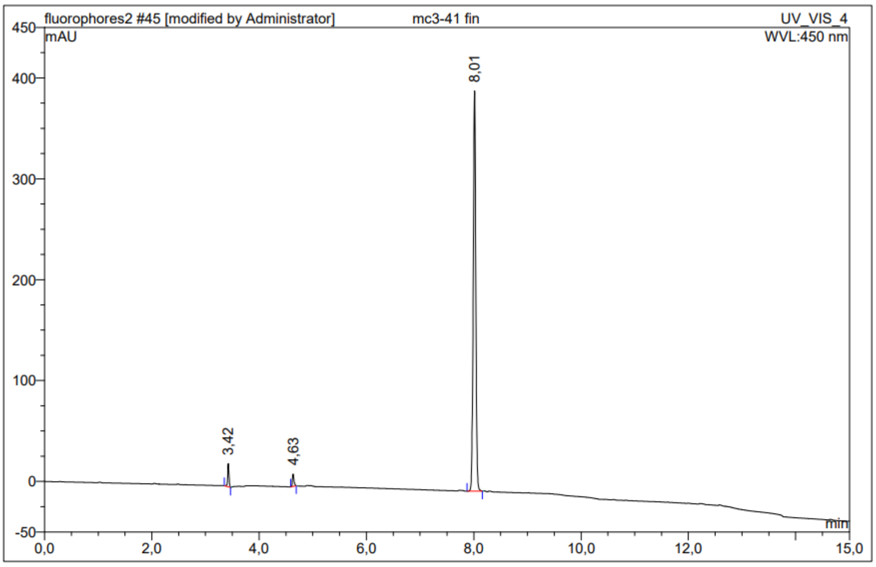

No.	Ret.Time min	Peak Name	Height mAU	Area mAU*min	Rel.Area %	Amount	Resolution(EP)
1	3,42	n.a.	22,967	0,670	2,92	n.a.	25,65
2	4,63	n.a.	12,368	0,394	1,72	n.a.	49,86
3	8,01	n.a.	396,759	21,889	95,36	n.a.	n.a.
Total:			432,095	22,953	100,00	0,000	

Compound 46 (254 and 450 nm)

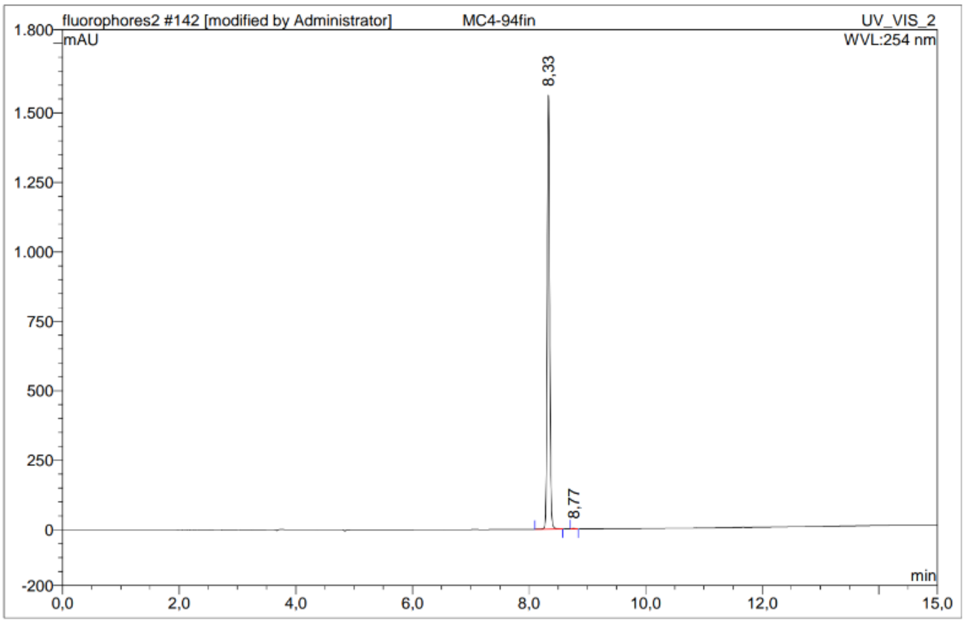

No.	Ret.Time min	Peak Name	Height mAU	Area mAU*min	Rel.Area %	Amount	Resolution(EP)
1	8,33	n.a.	1560,987	78,286	99,79	n.a.	4,94
2	8,77	n.a.	2,733	0,163	0,21	n.a.	n.a.
Total:			1563,720	78,449	100,00	0,000	

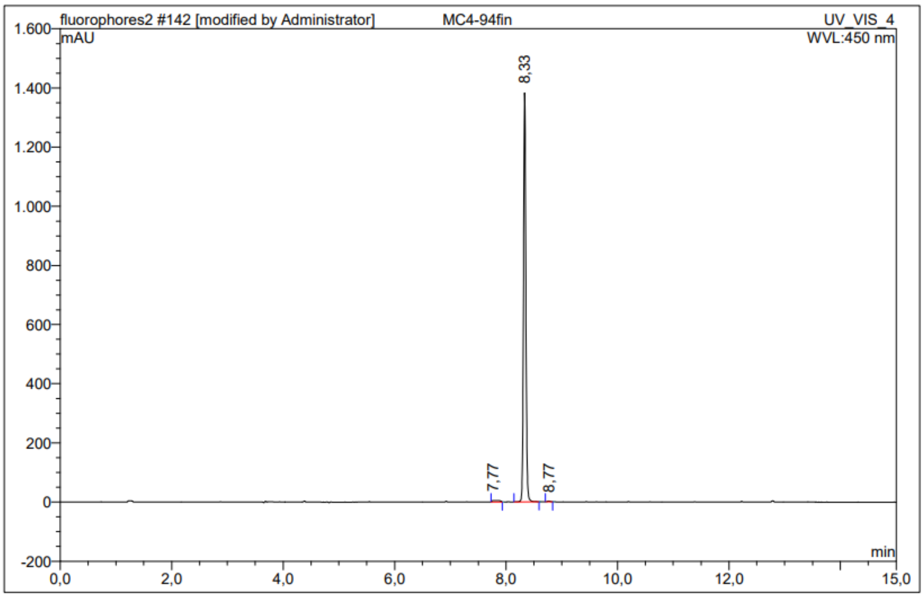

No.	Ret.Time min	Peak Name	Height mAU	Area mAU*min	Rel.Area %	Amount	Resolution(EP)
1	7,77	n.a.	4,400	0,710	1,00	n.a.	3,14
2	8,33	n.a.	1382,883	69,913	98,78	n.a.	4,89
3	8,77	n.a.	2,572	0,157	0,22	n.a.	n.a.
Total:			1389,855	70,780	100,00	0,000	

^{1}H NMR spectrum of **7**

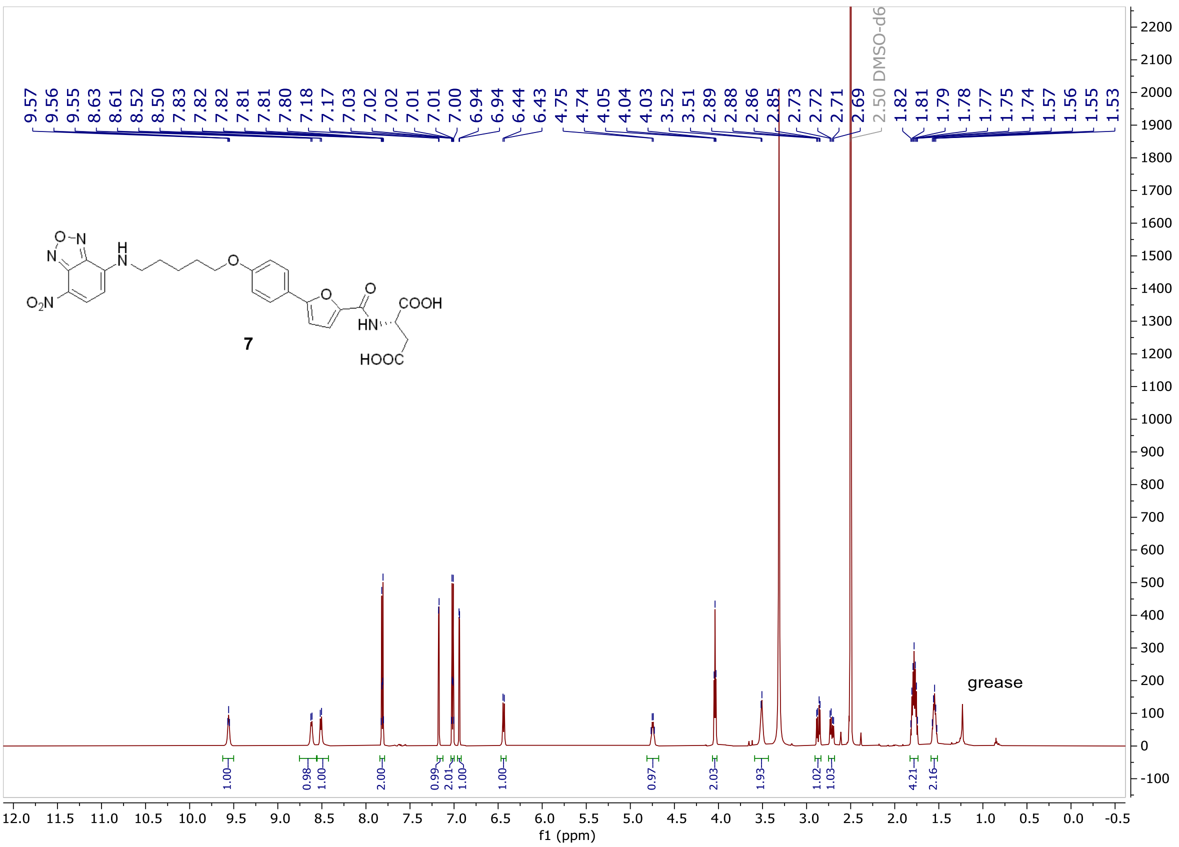

^{1}H NMR spectrum of **22**

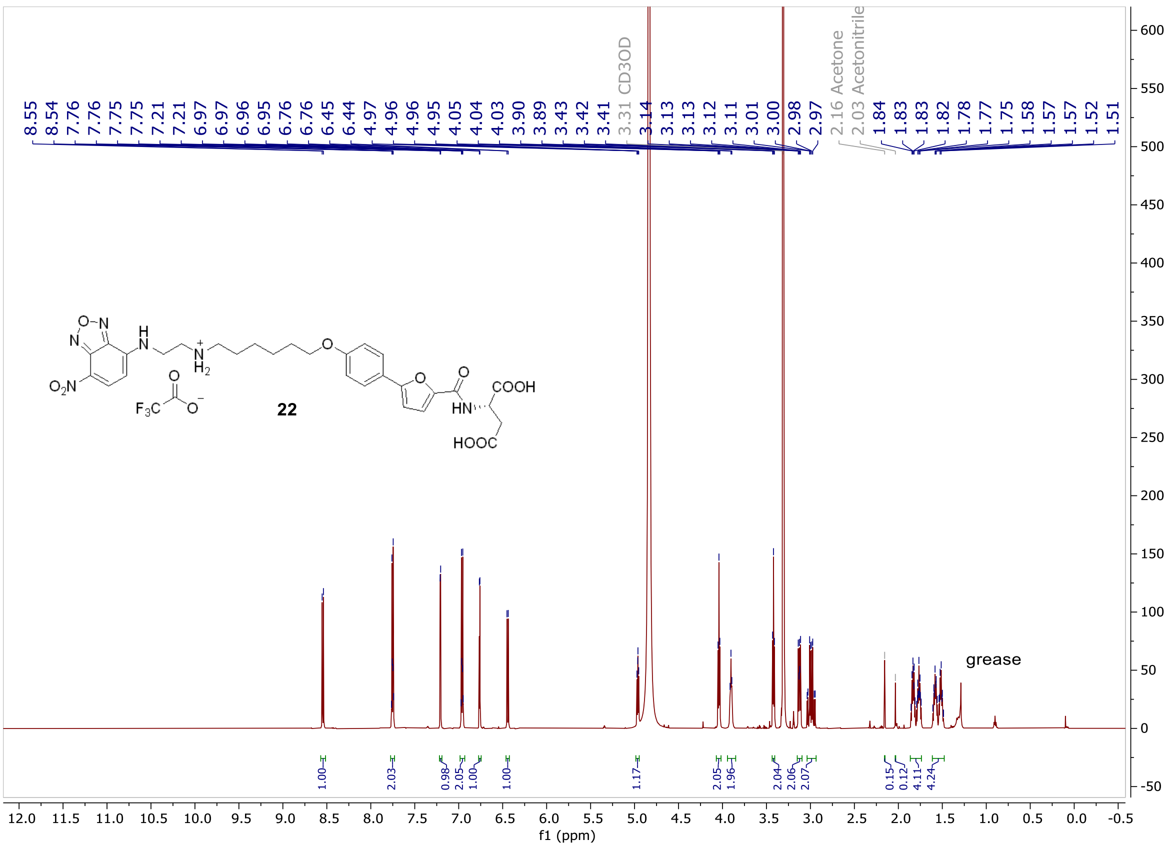

^{1}H NMR spectrum of **46**

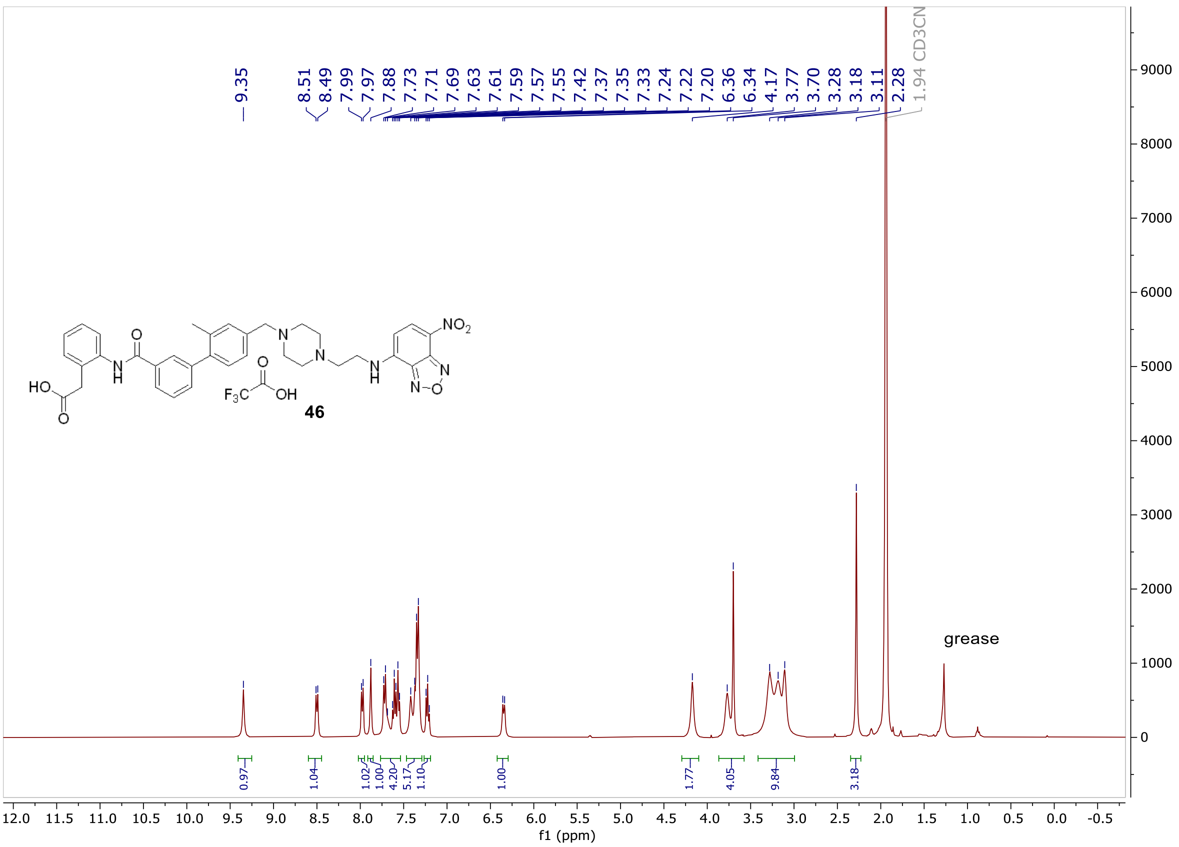

(1) Christiansen, E.; Hudson, B. D.; Hansen, A. H.; Milligan, G.; Ulven, T. Development and Characterization of a Potent Free Fatty Acid Receptor 1 (FFA1) Fluorescent Tracer. *J. Med. Chem.* **2016**, *59*, 4849-4858.
(2) Hansen, A. H.; Sergeev, E.; Pandey, S. K.; Hudson, B. D.; Christiansen, E.; Milligan, G.; Ulven, T. Development and Characterization of a Fluorescent Tracer for the Free Fatty Acid Receptor 2 (FFA2/GPR43). *J. Med. Chem.* **2017**, *60*, 5638-5645.
(3) Uchiyama, S.; Santa, T.; Okiyama, N.; Fukushima, T.; Imai, K. Fluorogenic and fluorescent labeling reagents with a benzofurazan skeleton. *Biomed. Chromatogr.* **2001**, *15*, 295-318.
(4) Rurack, K.; Spieles, M. Fluorescence quantum yields of a series of red and near-infrared dyes emitting at 600-1000 nm. *Anal. Chem.* **2011**, *83*, 1232-1242.
(5) Brouwer, A. M. Standards for photoluminescence quantum yield measurements in solution (IUPAC Technical Report). *Pure Appl. Chem.* **2011**, *83*, 2213-2228.
(6) Menges, F. Spectragryph-optical spectroscopy software version 1.2.16. **2022**.
